# Synthesis and antimicrobial activity of aminoalkyl resveratrol derivatives inspired by cationic peptides

**DOI:** 10.1080/14756366.2022.2146685

**Published:** 2023-01-04

**Authors:** Rubén Cebrián, Ricardo Lucas, María Victoria Fernández-Cantos, Koen Slot, Pablo Peñalver, Marta Martínez-García, Antonio Párraga-Leo, María Violante de Paz, Federico García, Oscar P. Kuipers, Juan Carlos Morales

**Affiliations:** a Department of Molecular Genetics, Groningen Biomolecular Sciences and Biotechnology Institute, University of Groningen, Groningen, The Netherlands; b Department of Clinical Microbiology, Instituto de Investigación Biosanitaria ibs. GRANADA, University Hospital Clínico San Cecilio, Granada, Spain; c Department of Organic and Pharmaceutical Chemistry, School of Pharmacy, University of Seville, Seville, Spain; d Department of Biochemistry and Molecular Pharmacology, Instituto de Parasitología y Biomedicina López Neyra, CSIC, PTS Granada, Armilla, Granada, Spain

**Keywords:** Resveratrol, aminoalkyl, membrane permeabilization, antimicrobial, anaerobic bacteria, synergism, toxicity, haemolysis

## Abstract

Antimicrobial resistance is a global concern, far from being resolved. The need of new drugs against new targets is imminent. In this work, we present a family of aminoalkyl resveratrol derivatives with antibacterial activity inspired by the properties of cationic amphipathic antimicrobial peptides. Surprisingly, the newly designed molecules display modest activity against aerobically growing bacteria but show surprisingly good antimicrobial activity against anaerobic bacteria (Gram-negative and Gram-positive) suggesting specificity towards this bacterial group. Preliminary studies into the action mechanism suggest that activity takes place at the membrane level, while no cross-resistance with traditional antibiotics is observed. Actually, some good synergistic relations with existing antibiotics were found against Gram-negative pathogens. However, some cytotoxicity was observed, despite their low haemolytic activity. Our results show the importance of the balance between positively charged moieties and hydrophobicity to improve antimicrobial activity, setting the stage for the design of new drugs based on these molecules.

## Graphical abstract

**Figure:**
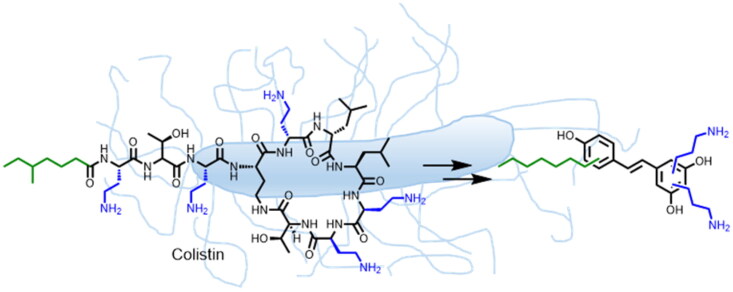

## Introduction

The increasing number of antimicrobial-resistant bacteria together with the scarce number of antimicrobial drugs approved during the last decades is alarming. The current therapeutical options to combat drug-resistant pathogen infections are limited, expensive, and associated with high mortality^1^. Despite the enormous efforts of the international community in antimicrobial research, only a few drugs have been approved recently for clinical use. In most cases, these approved antimicrobials belong to known families with small chemical modifications. These are only short-term solutions since the resistance mechanisms against them are already established in nature^2^^,^^3^. This problem is especially relevant in the case of Gram-negative bacteria. The presence of the outer membrane prevents them from the biocidal activity of many antibiotics, acting as a permeability barrier^4^. It is not surprising that Gram-negative pathogenic bacteria are heading the list of bacteria for which new antimicrobials are critically needed according to the World Health Organisation^5^. The prediction is that in 2050 drug-resistant pathogen infections will be the first cause of death. This means approximately 10 million deaths per year, 14 times more than the current 0.7 million^6^. New and safe antimicrobial drugs are necessary. Drugs with a new mechanism of action and/or target to overcome already established resistance mechanisms will be highly desirable.

Cationic antimicrobial peptides are among the most promising new drugs. They are characterised by their amphiphilic character in which hydrophobic and hydrophilic regions are oppositely distributed on the structure. This topology is especially useful in permeating or disrupting bacterial membranes eventually leading to cell death^7–9^. In fact, some membrane-targeting amphipathic antimicrobials, such as colistins (active against Gram-negative bacteria) and lipopeptides (e.g., daptomycin, active against Gram-positive bacteria), have become last resource drugs for the treatment of multi-drug resistant bacteria. The resistance levels against these drugs remain low^10^. Despite their similar structures, these two classes of antibiotics have distinct modes of action and clinical uses. Colistins target lipopolysaccharide (LPS) in Gram-negative bacteria, inducing its derangement by the displacement of divalent cations involved in its stability. Daptomycin is negatively charged and requires Ca^2+^ to interact with the anionic phosphatidylglycerol of the bacterial membrane^11^. Unlike colistin, it is only used to treat infections caused by drug-resistant Gram-positive bacteria^10^^,^^12^^,^^13^. The design of new molecules mimicking colistin/lipopeptides or other cationic amphipathic antimicrobials properties could render new drugs with similar antimicrobial activities. Over the past decade, several groups have focussed on the development of cationic antimicrobial mimetics and some of them (brilacidin or LTX109) have successfully entered Phase II clinical trials^14^.

Thus, antimicrobial phenolic compounds could be used as starting scaffolds. Through chemical modification, we have tried to mimic the characteristics of cationic antimicrobial peptides obtaining potential new drugs against bacteria. Cationic curcuminoids are 100 times more active against Gram-negative bacteria than the natural product curcumin^15^. Mangostin and kaempferol-based mimetics of cationic antimicrobials have also been developed with promising activity in a murine corneal infection study^14^^,^^16^^,^^17^. Recently, materials based on arginine-substituted poly(gallic acid) have been described and showed antimicrobial activity against both Gram-negative and Gram-positive bacteria^18^. Besides, cationic amino resveratrol (RES) derivatives, imine-RES, or aza-RES analogues have been prepared for different therapeutic uses, such as neurodegenerative diseases,^19^ cancer^20–25^^,^ or inflammation^16^^,^^17^. 3′-Amino methylated RES derivatives showed toxicity against *Leishmania infantum* and low toxicity when tested on normal haemopoietic cells^26^.

**Figure 1.**
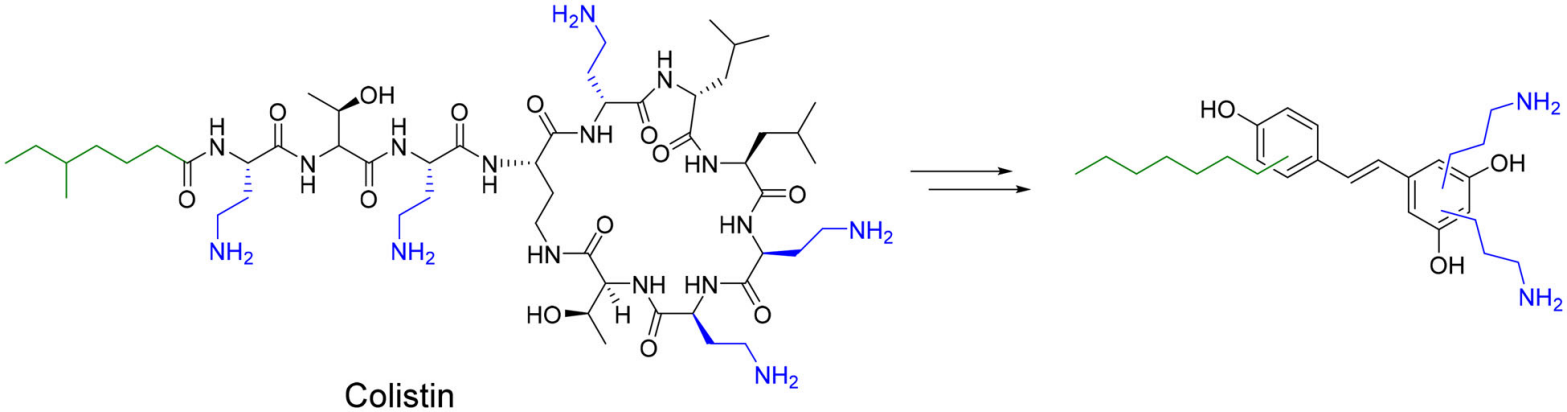
Colistin inspiration for aminoalkyl resveratrol derivatives (resveratrol, RES drawn in black colour.

**Figure 2.**
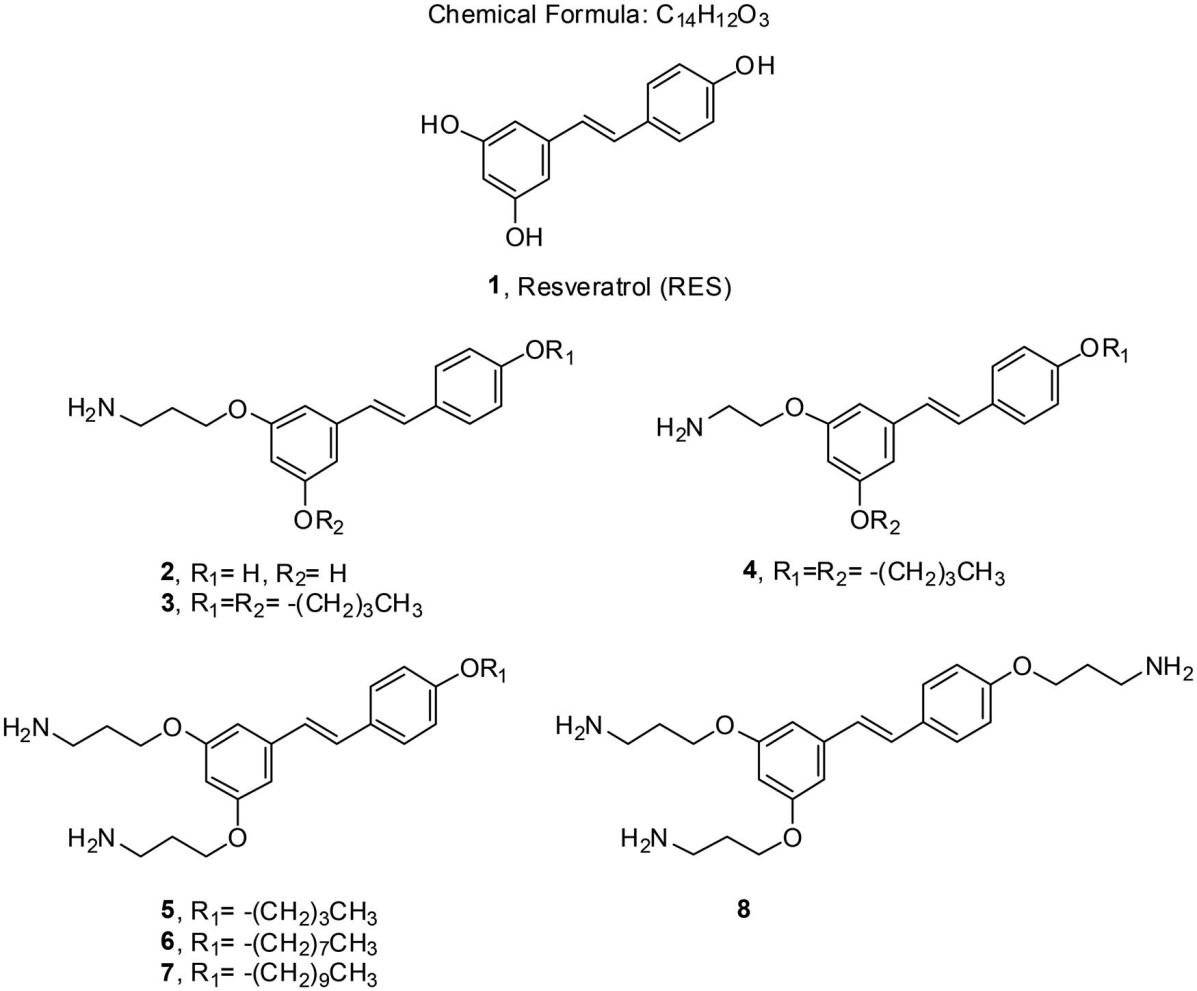
Resveratrol (RES) and amino RES derivatives prepared in this work.

Here, we describe the design and synthesis of aminoalkyl RES derivatives mimicking the amphipathic characteristics of cationic antimicrobials (Figure 1). We have prepared RES derivatives containing one, two, or three *O*-aminoethyl or *O*-aminopropyl groups attached to the stilbene scaffold together with a small lipophilic tail (Figure 2). We have examined their antimicrobial activity against a panel of Gram-negative and Gram-positive bacteria, both, aerobic and anaerobic growing bacteria, their haemolytic activity, and their toxicity against human cell lines. The mode of action of the best compound, i.e., compound number **5** was investigated together with its potential synergistic effect with other commonly used antibiotics.

## Results and discussion

### Synthesis of aminoalkyl RES derivatives

**Scheme 1.**
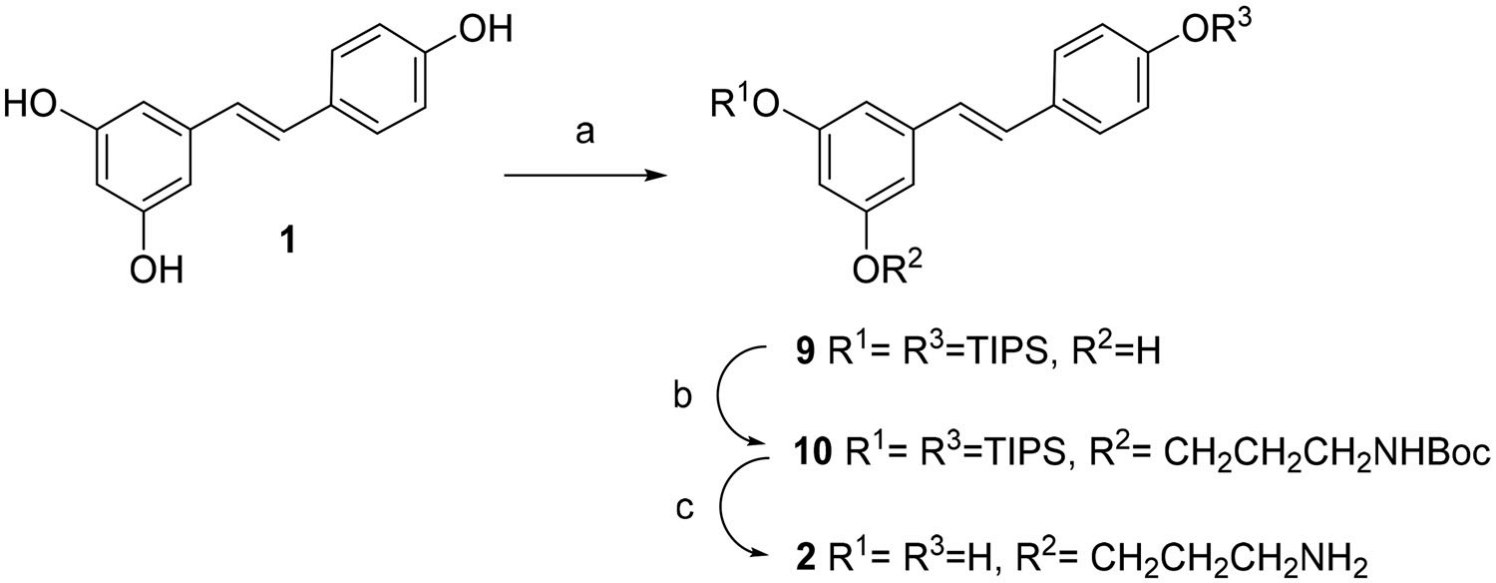
Synthesis of compound **2**. Reagents and conditions: (a) TIPS chloride (2.0 equiv), DIPEA (2.0 equiv), DMAP (2.0 equiv), DMF, -15° C for 10 min, then rt for 16 h; (b) ICH_2_CH_2_CH_2_NHBoc, K_2_CO_3_, DMF; (c)TFA, THF-H_2_O.

Resveratrol monoamine derivative **2** was synthesised from resveratrol by random silylation. Disilyl RES derivative **9** was prepared by random silylation from RES. We used 4-(dimethylamino)pyridine (DMAP) and N,N-diisopropylethylamine (DIPEA) as bases (Scheme 1) in order to improve the previously reported regioselectivity of the reaction of RES at positions 3 and 4′ (Scheme 1)^27^^,^^28^. Compound **9** was obtained at a higher yield than using other bases such as imidazole^29^. Disilyl RES **9** was then alkylated by reaction with tert-butyl (3-iodopropyl)carbamate and K_2_CO_3_ in dry N,N-dimethylformamide (DMF)^30^ to get protected resveratrol derivative **10**. Tert-Butyl (3-iodopropyl)carbamate is readily available from 3-aminopropan-1-ol after tert-butyloxycarbonyl (BOC) protection and iodination^30^. In order to remove BOC and silyl protecting groups at once, compound **10** was treated with trifluoroacetic acid (TFA) in tetrahydrofuran (THF) and the crude was purified by Sephadex G50 affording compound **2**.

**Scheme 2.**
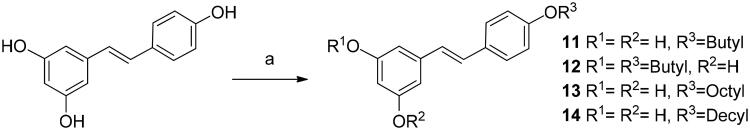
Synthesis of alkyl resveratrol derivatives **11–14**. Reagents and conditions: (a) 1-iodobutane, 1-bromooctane or 1-bromodecane, K_2_CO_3_, DMF.

The preparation of compounds **3**–**7** started with the synthesis of alkyl RES derivatives **11**–**14** (Scheme 2). They were synthesised by random alkylation of resveratrol with the corresponding iodo or cinetobact followed by purification by silica-gel column chromatography^31^.

**Scheme 3.**
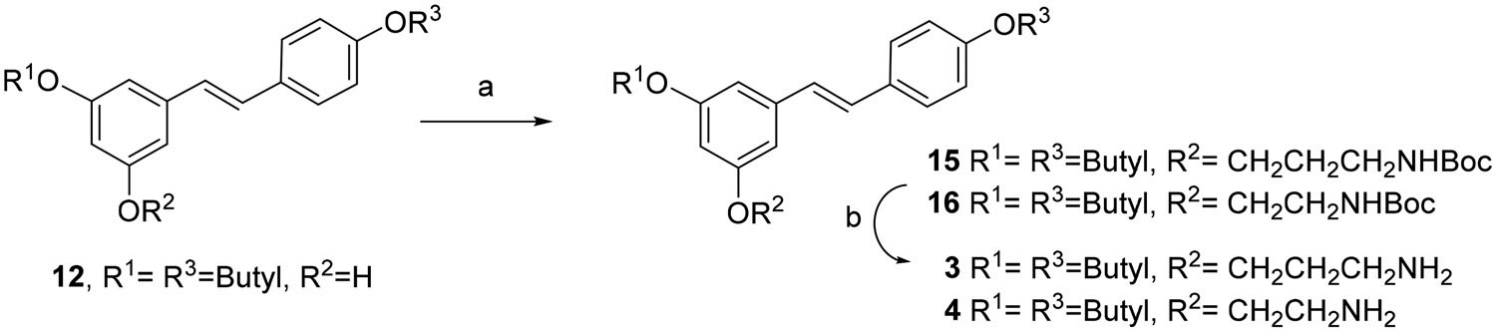
Synthesis of monoamino alkyl resveratrol derivatives **3–4**. Reagents and conditions: (a) ICH_2_CH_2_CH_2_NHBoc or BrCH_2_CH_2_NHBoc, K_2_CO_3_, DMF; (b) TFA, THF.

Reaction of 3,4′-dibutyl resveratrol **12** with tert-butyl (3-iodopropyl)carbamate under the alkylation conditions described above, yielded compound **15** (Scheme 3). Compound **16** was obtained by reaction of **12** with commercially available tert-butyl (2-bromoethyl)carbamate. Then, cleavage of BOC protecting groups with TFA afforded the amino alkyl RES derivatives **3** and **4** in good yields (Scheme 3).

**Scheme 4.**
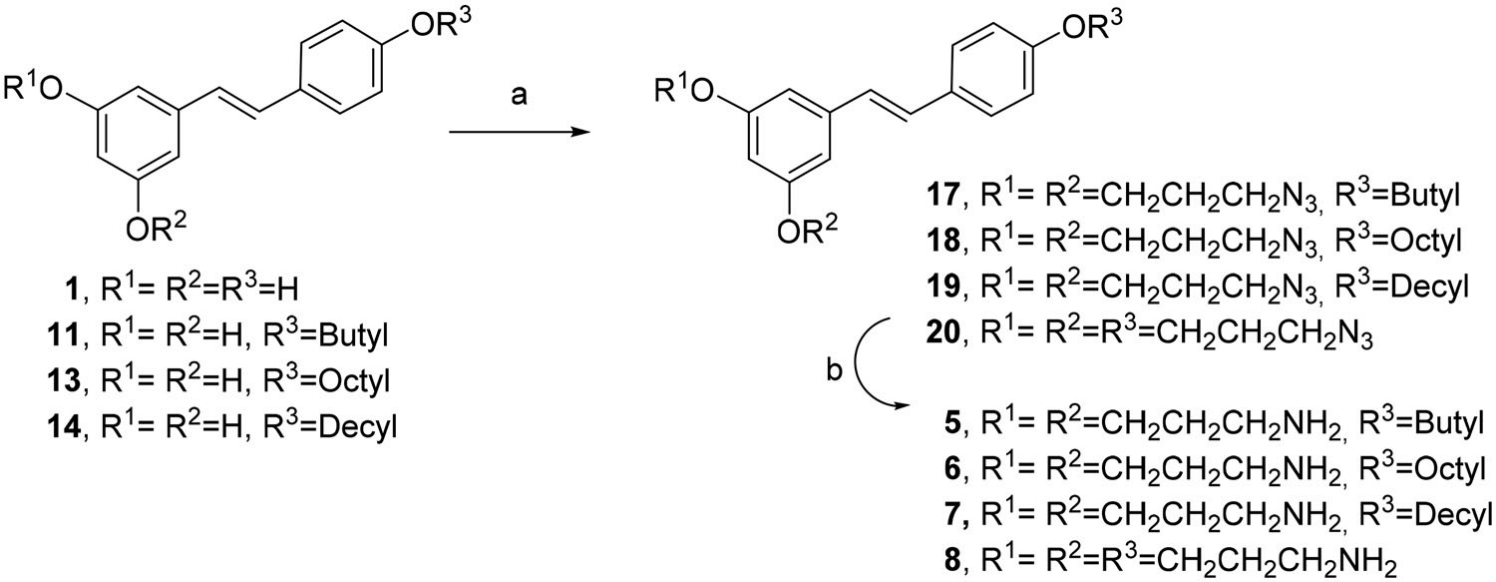
Synthesis of diamino and triamino alkyl resveratrol derivatives **5–8**. Reagents and conditions: (a) i. BrCH_2_CH_2_CH_2_Cl, K_2_CO_3_, DMF, 80 °C, 5 h. ii) NaN_3_, DMF, 50 °C, 16 h; (b) PPh_3_, THF, 16 h.

We tried an alternative methodology to prepare compounds **5**–**8**, avoiding the preparation of tert-butyl (3-iodopropyl)carbamate (Scheme 4). In this case, the reaction of RES **1** and mono alkyl RES derivatives **11**, **13** and **15** with commercially available 1-bromo-3-cloropropane and potassium carbonate in dry DMF followed by chloride displacement with sodium azide afforded compounds **17**–**20**. The azido group was readily transformed into the amino group by Staudinger reduction with PPh_3_ in THF yielding diamino and triamino alkyl RES derivatives **5**–**8**^32^.

### Antimicrobial activity of RES derivatives

Initially, we tested the new RES derivatives against a collection of Gram-negative and Gram-positive bacteria growing aerobically using polymyxin B and daptomycin as a control respectively. In general, Gram-positive bacteria were overall more sensitive to the new compounds than Gram-negative (Table 1). Only aminoalkyl RES derivative **5** displayed antimicrobial activity against all the bacteria examined, with minimal inhibitory concentration (MIC) ranging from 13.3 to 64 µM (Table 1). In the case of Gram-positive tested bacteria, compound **5** was also the most active of the series, with MIC values ranging from 3.3 to 36.7 µM. Other amino RES derivatives also displayed antimicrobial activity, although they were strain-specific and MIC values were higher than those observed for **5** (Table 1). Interestingly, the colistin-resistant *E. coli* NCTC 13846 was the most sensitive to amino RES **5,** and the highly-colistin-resistant *Enterobacter cloacae* LMG 02783 was also sensitive (Table 1), which suggests alternative action mechanisms/targets.

**Table 1.** Minimal inhibition concentration (MIC) observed for the different RES derivatives against a panel of Gram-negative and Gram-positive aerobically growing bacteria. -, these bacteria were resistant to the higher tested concentration (128 µM).

		Pol B	Dap	RES	2	3	4	5	6	7	8
**Gram-negative**	*Acinetobacter baumannii* LMG 01041	1.7 ± 0.3	nd	–	–	128 ± 0	128 ± 0	32 ± 0	–	–	–
	*Klebsiella aerogenes* LMG 02094	1.3 ± 0.3	nd	–	–	–	–	64 ± 0	–	–	–
	*Enterobacter cloacae* LMG 02783	>128	nd	–	–	128 ± 0	128 ± 0	32 ± 0	–	–	–
	*Escherichia coli* LMG 8224	0.7 ± 0.1	nd	–	–	128 ± 0	128 ± 0	16 ± 0	128 ± 0	–	128 ± 0
	*E. coli* NCTC 13846	8 ± 0	nd	–	–	–	–	13.3 ± 2.6	–	–	128 ± 0
	*K. pneumoniae* LMG 20218	4 ± 0	nd	–	–	–	–	64 ± 0	–	–	–
	*Pseudomonas aeruginosa* PAO1	1 ± 0	nd	–	–	–	–	64 ± 0	–	–	–
	*Salmonella. enterica* LMG 07233	4 ± 0	nd	–	–	–	–	32 ± 0	–	–	128 ± 0
**Gram-positive**	*Bacillus cereus* ATCC 10987	nd	0.625 ± 0	–	–	128 ± 0	85.3 ± 21.3	26.7 ± 5.3	85.3 ± 21.3	128 ± 0	–
	*Bacillus cereus* ATCC 14574	nd	0.625 ± 0	–	–	106.7 ± 21.3	64 ± 0	32 ± 0	32 ± 0	128 ± 0	128 ± 0
	*Enterococcus faecalis* V583	nd	1 ± 0	–	–	–	–	26.7 ± 5.3	–	–	–
	*E. faecalis* LMG 8222	nd	1 ± 0	–	–	53.3 ± 10.6	42.7 ± 10.6	16 ± 0	16 ± 0	–	128 ± 0
	*E. faecalis* LMG 16216	nd	1 ± 0	–	–	64 ± 0	64 ± 0	13.3 ± 2.6	16 ± 0	–	–
	*E. faecium* LMG 11423	nd	2 ± 0	–	–	64 ± 0	32 ± 0	16 ± 0	8 ± 0	–	128 ± 0
	*E. faecium* LMG 16003	nd	2 ± 0	–	–	128 ± 0	128 ± 0	16 ± 0	–	–	128 ± 0
	*Staphylococcus aureus* LMG 15975	nd	0.312 ± 0	–	128 ± 0	53.3 ± 10.6	32 ± 0	3.3 ± 0.6	16 ± 0	128 ± 0	128 ± 0
	*S. aureus* LMG 8224	nd	1 ± 0	–	128 ± 0	42.7 ± 18.4	42.7 ± 18.4	8 ± 0	8 ± 0	64 ± 0	128 ± 0
	*S. aureus* LMG 10147	nd	0.312 ± 0	–	–	–	–	13.3 ± 4.6	128 ± 0	–	128 ± 0

The concentrations are expressed in µM ± standard error. Pol B, polymyxin B. Dap, daptomycin. nd, no determined.

Although several anaerobic bacteria are related to serious health problems, anaerobes are in general underrepresented in studies determining the activity spectrum of novel antimicrobial compounds. This might be due to the more difficult and time-consuming methodologies required for culturing anaerobic bacteria. We examined the antibacterial activity of the new amino RES derivatives against a panel of clinically relevant Gram-negative and Gram-positive anaerobic bacteria, including *Bacteroides*, *Parabacteroides*, *Clostridium,* and *Clostridioides* strains. Interestingly, no clear differences were observed between the susceptibility of Gram-positive and Gram-negative anaerobic bacteria (Table 2). This is a remarkable fact considering that the novel classes of antibiotics that have reached the market in recent years are primarily effective against Gram-positive bacteria.^33^ Surprisingly, these bacteria were much more sensitive to the amino RES derivatives than the aerobic bacteria, except for *Parabacteroides merdae* (Table 2). Similar to the results found on aerobic bacteria, compound **5** was the only derivative with antimicrobial activity against all tested bacteria. However, compound **6** displayed higher activity than **5** (except for *P. merdae*) (Table 2). The MIC values ranged for amino RES **5** from 8 to 64 µM (most strains at just 16 µM) in the case of Gram-negative bacteria and, from 2 to 21.3 µM for Gram-positive bacteria (Table 2). For compound **6** these activities were even lower, from 8 to 18.6 µM for Gram-negative bacteria and from 1 to 4 µM for Gram-positive bacteria (Table 2). Other amino RES derivatives such as **3**, **4** or **7** that were inactive or scarcely active against Gram-negative aerobically growing bacteria, also displayed antimicrobial activity in the low micromolar range against the different *Bacteroides* strains tested (Table 2). The activity observed against Gram-negative anaerobic bacteria is remarkable considering that other cationic amphipathic antimicrobials such as colistin are not active against this bacterial group.^34^ In the case of Gram-positive anaerobes, the activity observed was close to that observed for daptomycin, particularly for compound **6.** To investigate if anaerobic conditions enhanced the antimicrobial activity of the amino RES derivatives, a MIC test was performed for *E. coli* LMG 8224, *K. pneumoniae* LMG 20218, and *P. aeruginosa* PAO1 under anaerobic conditions. No enhancement of their antimicrobial activity was observed, suggesting that amino RES derivatives present certain specificity for strict anaerobic bacteria.

**Table 2.** Minimal inhibition concentration (MIC) observed for the different RES derivatives against a panel of Gram-negative and Gram-positive anaerobic bacteria. -, these bacteria were resistant to the higher tested concentration (128 µM).

		Pol B	Dap	RES	2	3	4	5	6	7	8
**Gram-negative**	*Bacteroides ovatus* 3_8_47FAA	128 ± 0	nd	–	–	21.3 ± 5.3	32 ± 0	16 ± 0	8 ± 0	32 ± 0	–
	*B. fragilis* NCTC 9343	64 ± 0	nd	–	–	32 ± 0	32 ± 0	16 ± 0	18.6 ± 7	64 ± 0	–
	*B. salyersiae* DSM 18765	>128	nd	–	–	32 ± 0	32 ± 0	16 ± 0	13.3 ± 2.6	53.3 ± 10.6	–
	*B. xylanisolvens* DSM 18836	32 ± 0	nd	–	128 ± 0	16 ± 0	16 ± 0	8 ± 0	8 ± 0	32 ± 0	128 ± 0
	*Parabacteroides merdae* CL03T12C32	64 ± 0	nd	–	–	–	–	64 ± 0	–	–	–
**Gram-positive**	*Clostridium botulinum* CECT 551	nd	4 ± 0	128 ± 0	128 ± 0	8 ± 0	16 ± 0	2 ± 0	1 ± 0	8 ± 0	128 ± 0
	*C. perfringens* CECT 376	nd	2 ± 0	–	128 ± 0	16 ± 0	16 ± 0	21.3 ± 9.2	2 ± 0	–	–
	*C. tetani* CECT 426	nd	2 ± 0	–	–	13.3 ± 4.6	16 ± 0	8 ± 0	1 ± 0	21.3 ± 9.2	–
	*Clostridioides difficile* CECT 531	nd	2 ± 0	–	128 ± 0	16 ± 0	16 ± 0	4 ± 0	4 ± 0	16 ± 0	–

Pol B, polymyxin B. Dap, daptomycin. nd, no determined. The concentrations are expressed in µM ± standard error.

**Figure 3.**
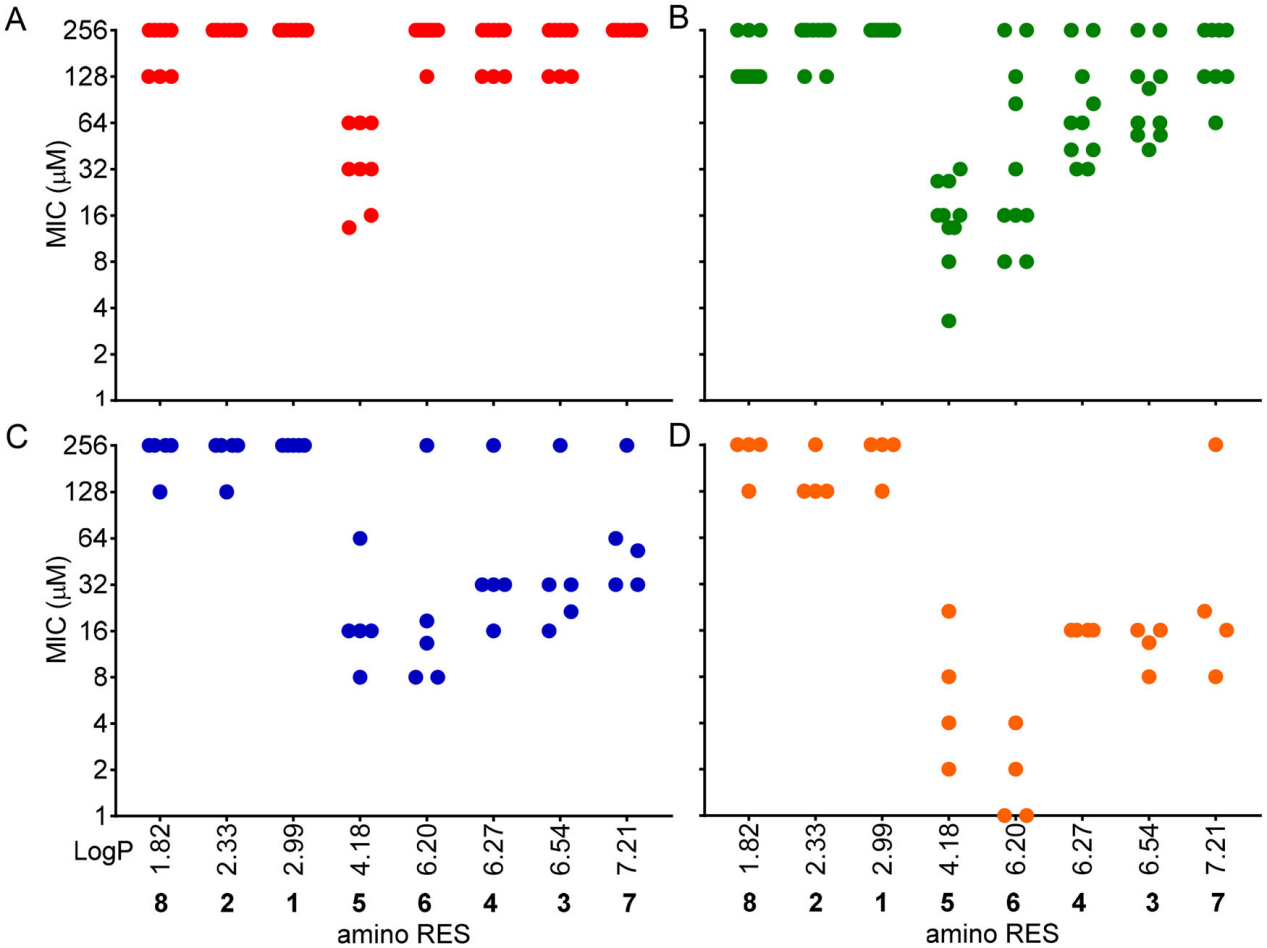
MICs distribution according to the hydrophobicity (LogP) of the amino RES derivatives. A) Gram-negative aerobic bacteria, B) Gram-positive aerobic bacteria, C) Gram-negative anaerobic bacteria, D) Gram-positive anaerobic bacteria. Each dot is representing the MIC for a tested bacteria against each compound. For those bacteria for which MIC was not reached, 256 µM was considered for this graph.

The hydrophobicity of the molecules plays a key role in antimicrobial activity and it is well known that an increase in the hydrophobicity usually improves the antimicrobial activity of the drugs, but with limitations^35^^,^^36^. Figure 3 is representing the observed MICs of each compound *versus* their hydrophobicity (LogP) calculated using the Molinspiration tool^37^ (https://molinspiration.com/). Interestingly, molecules with low hydrophobicity, such as **8**, **2**, and **1** (LogP 1.82, 2.33, and 2.99, respectively), were inactive or scarcely active (Figure 3). The most active amino RES (**5**) displayed a medium hydrophobicity (LogP 4.18) in this series and it was the most active. The activity observed for the rest of the designed amino RES **6**, **4**, **3,** and **7** (LogP 6.20, 6.27, 6.54, and 7.21, respectively) gradually decreased based on the LogP value (Figure 3) except for **6** in the case of Gram-positive anaerobes which was more active than **5** (Figure 3(D)).

### Activity of amino RES derivatives at the bacterial membranes

**Figure 4.**
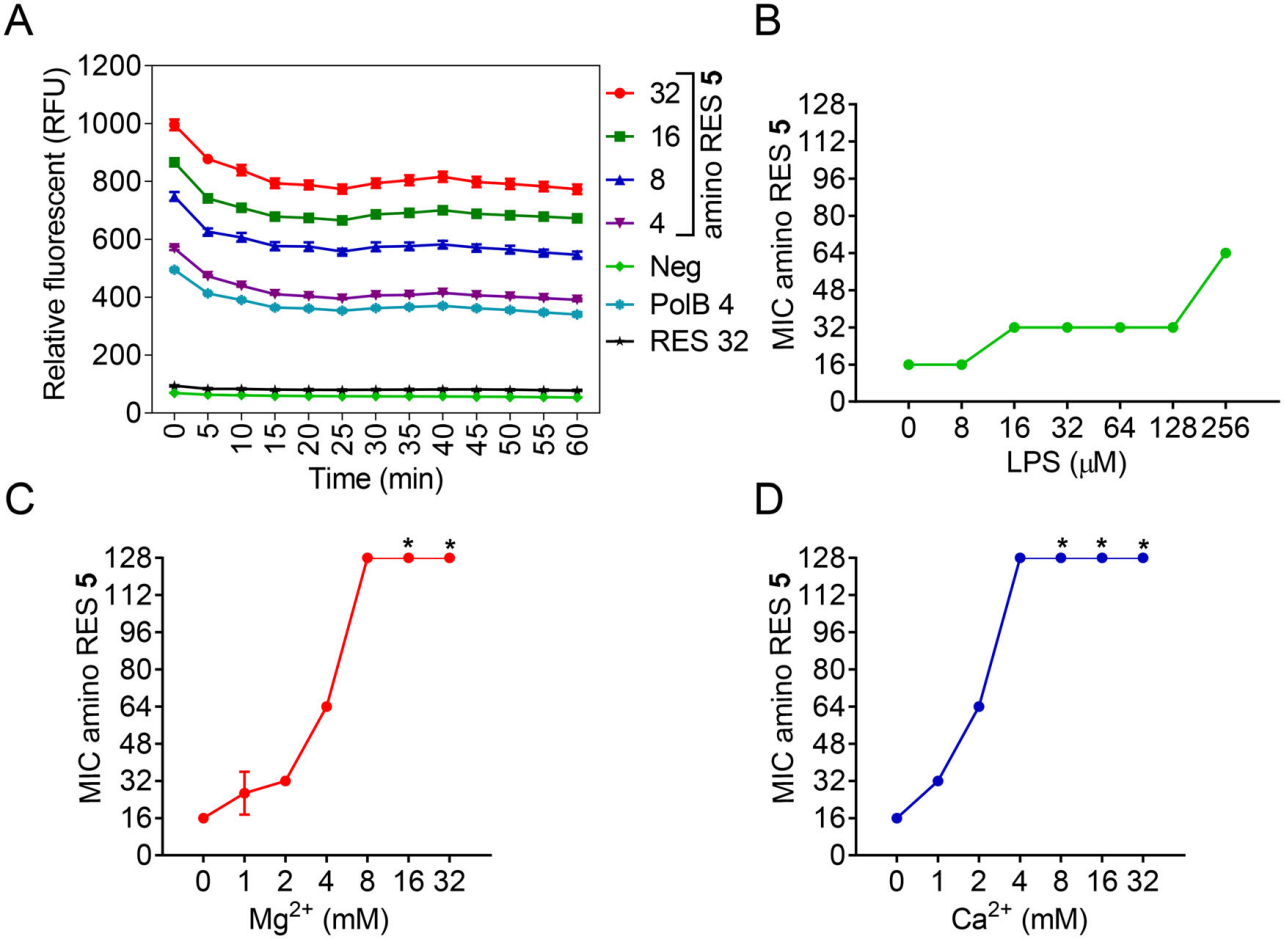
Activity of amino RES derivatives on the outer membrane of *E. coli* LMG8224. A) Outer-membrane permeabilization by different amino RES **5** concentrations (µM). RES 32, resveratrol control at 34 µM, PolB polymyxin B positive control at 4 µM. Neg, negative control. RFU, random fluorescence units. Effect of the outer membrane main component LPS (B) and the outer membrane stabilising agents Mg^2+^ (C) and Ca^2+^ (D) on the antimicrobial activity of amino RES **5**. For the points labelled with *, the MIC was higher than 128 µM.

Cationic antimicrobials are well known for their ability to bind to negatively charged bacterial membranes. Thus, we decided to analyse in depth the effect of the best candidate (**5**) in both Gram-negative and Gram-positive bacterial membranes. The outer membrane in Gram-negative bacteria acts as a permeability barrier that impairs antibiotics to reach their target inside the cell.^4^ Several cationic antimicrobial drugs are characterised to be amphipathic, with a positively charged hydrophilic domain and a hydrophobic domain. As a consequence, these drugs can bind through electrostatic interactions with the negatively charged bacterial surface. Subsequently, the hydrophobic domain can be inserted into the membrane, forming stable and disruptive pores that produce the leaking of the intracellular content and, therefore, cell death by lysis^38^^,^^39^. Alternatively, they can disrupt the membranes inducing an alteration in their permeability^40^. To understand the effect of amino RES **5** against Gram-negative bacteria, we explored its activity in the outer membrane using *E. coli* LMG 8224 as a model. We measured the outer membrane permeability under different concentrations of amino RES **5** using the hydrophobic fluorescent probe 1-N-phenylnaphthylamine (NPN) and polymyxin B as a positive control^41^. A dose-related response was observed for amino RES **5**, indicating a perturbation/disruption in the membrane while no effect was observed for RES (Figure 4(A)).

**Figure 5.**
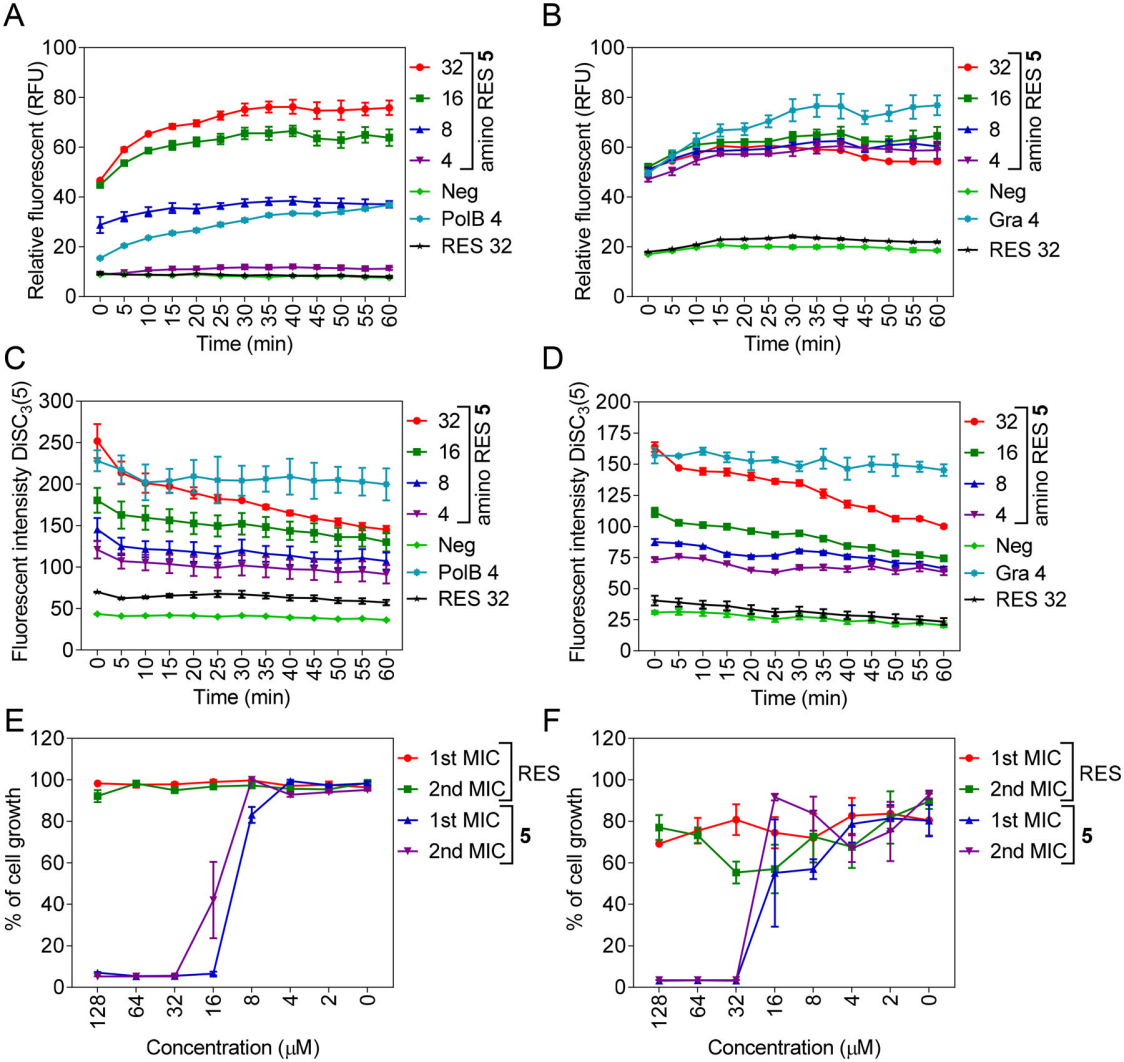
Membrane permeabilization by amino RES **5** in *E. coli* LMG 8224 (A) and Gram-positive bacteria *B. cereus* ATCC 10987 (B). RFU, random fluorescence units. C, membrane depolarisation (DiSC_3_(5)) for *E. coli* LMG 8224 and *B. cereus* ATCC 10987 (D). RES, resveratrol control, Pol B positive control and Gra, gramicidin S positive control. The concentrations are expressed in µM. E and F, bactericidal mode of action for *E. coli* LMG 8224 and *B. cereus* ATCC 10987 respectively. The data are expressed as % of the cell growth respect the negative control.

To get a deeper insight into the potential outer membrane target, we tested the antibacterial activity of compound **5** in the presence of the main outer membrane component, the lipopolysaccharide (LPS). We took into account the presence of two divalent cations, Mg^2+^ and Ca^2+^, which help to stabilise and maintain the integrity of the outer membrane by binding in between adjacent LPS molecules. The effect of LPS on the antimicrobial activity was quite low, and only at the highest concentration tested an 8-fold MIC increment was observed (Figure 4(B)). Nevertheless, the addition of divalent cations strongly antagonised the antimicrobial activity of amino RES **5** (Figures 5(C,D)), especially Ca^2+^. Similar results were observed for the cyclic cationic antibiotic polymyxin B, for which Ca^2+^ is also more active in decreasing the antimicrobial activity^42^.

To explore if a different effect on the outer membrane can explain the different antimicrobial effects observed for amino RES **5** in Gram-negative bacteria, we analysed the outer membrane permeabilization for the rest of amino RES derivatives in *E. coli* LMG 8224. We found that all the compounds were able to permeabilize the outer membrane (Supplementary Figure 1), but none of them was able to kill Gram-negative aerobic bacteria at low concentrations as in the case of amino RES **5**. This result suggests that the other derivatives were not able to induce stable pores capable of disrupting the membrane. Permeabilization without disruption and/or any toxical effect has been described for other drugs such as antimicrobial peptides, polymyxin derivatives, cationic polymers, cationic detergents, or chelators among others^40^^,^^42^. In the case of amino RES **2**, the permeabilization levels were low and only observed at the highest concentration used suggesting the important role of the alkyl chains in the membrane insertion. In fact, the second lowest outer membrane permeabilization was observed for amino RES **8**, another derivative without alkyl groups but strongly positively charged.

It is known that some antimicrobials present the ability to be translocated across biological membranes in a non-disruptive way, overcoming the impermeable nature of the membranes.^43^^,^^44^ These types of drugs interact with the membranes inducing transient and unstable perturbations that do not kill the cells, as described in the case of the highly cationic cell-penetrating peptides and other families of polyphenols^41^^,^^45^^,^^46^. The activity of amino RES **5** in the inner membrane permeability of *E. coli* LMG 8224 and in the membrane of the Gram-positive *B. cereus* ATCC 10987 was explored using the DNA-binding dye propidium iodide and polymyxin B and bacitracin as positive controls respectively^41^. As expected, no activity for RES was observed (Figure 5(A,B)). In contrast, amino RES **5** derivative was able to permeate the inner membrane of *E. coli* in a dose-related manner unlike in the Gram-positive *B. cereus* in which all tested concentrations permeabilize the membrane to PI in a similar range. These results suggest a more potent activity of amino RES **5** which could be related to its highest activity in Gram-positive bacteria.

In the case of *E. coli* LMG 8224, the rest of amino RES derivatives were also tested to analyse the effect of the alkyl and cationic group distribution on the interaction with the membrane (as above for the outer membrane). The highest permeabilization ratios were reached for derivatives **3**, **4**, **5**, **6**, and **7**, although, as previously, only compound **5** induced high permeabilization at low concentrations (Supplementary Figure 2). The absence of alkyl groups (amino RES **2** and **8**) causes lower levels of inner membrane permeabilization (Supplementary Figure 2).

The increase in membrane permeability usually causes the dissipation of the membrane potential. So, we investigated this parameter for *E. coli* and *B. cereus* treated with amino RES **5** using the potential-sensitive membrane dye DiSC_3_(5).^47^ An amino RES **5** dose-related increment of the fluorescent signal was observed for both bacteria indicating the membrane depolarisation and the dissipation of the membrane potential (Figure 5(C,D)).

Finally, considering that membrane-active antimicrobials usually show a bactericidal effect,^10^^,^^48–50^ we examined the nature of the antimicrobial activity. To do so, after the first MIC in a 96-well plate, the remaining cells were used to inoculate at 10% fresh MHB medium that was stored at 37 °C for 24 h. The nature of the antimicrobial activity was mainly bacterial as can be observed in Figure 5(C,G)).

Altogether, these results suggest an effect at the membrane level although other targets cannot be discarded. The highest sensibility observed for Gram-negative anaerobes could be related to structural differences in the outer membrane with respect to the aerobic Gram-negative bacteria^46^^,^^47^. The difference in sensitivity observed between Gram-negative and Gram-positive bacteria could be related to the presence of two protective layers in Gram-negative that could slow down the entrance of the drugs to the inner one, avoiding high accumulation at time. Thus, the higher effect in Gram-positive bacteria could be due to the disruption of the membrane produced by the drugs inside the cells. This system has been previously proposed for other polyphenols although no clear relation between the Gram-staining and susceptibility to polyphenols has been established, which suggests the important role of putative intracellular targets^46^.

### Combined activity of amino RES 5 and traditional antibiotics

The absence of cross-resistance with antibiotics in use is a desirable quality for newly designed drugs. For this reason, we tested the combined activity of the most active amino RES (**5**) with 24 antibiotics from the main antibiotic families against a selection of Gram-negative and Gram-positive bacteria. As expected for outer membrane-active compounds, no synergistic or antagonistic activity was observed in the case of Gram-positive bacteria,^40^^,^^51–53^ while some of the tested antibiotics were synergistically active with amino RES **5** (Table 3). The observed synergy was strain-specific (no synergy was observed in the case of *P. aeruginosa* PAO1). Overall, most of the synergistic antibiotics were hydrophobic compounds, highlighting rifampicin, which showed a synergistic effect in all tested bacteria. Other antibiotics, such as erythromycin, nalidixic acid, or novobiocin, were also broadly active in synergy with **5** (Table 3). Synergism of rifamycins, macrolides, or quinolones with polymyxin B nonapeptide inactive-derivatives has been previously reported, but not for combinations with amino coumarins.^53–56^ In fact, synergism of membrane-active molecules and hydrophobic antibiotics against Gram-negative bacteria is broadly reported, since the perturbation in the outer membrane produces the lack of the barrier function and such antibiotics can enter reaching their targets.^41^^,^^57–59^ In the case of the *Klebsiella* strain tested, also synergy with minocycline was observed. Finally, an astonishing synergism was also detected with the cationic drug polymyxin B in the case of *S. enterica* LMG 07233 (Table 3).

**Table 3.** Combined activity of amino RES **5** and traditional antibiotics against selected Gram-negative bacteria. AB, *A. baumannii* LMG 01041, ECl, *E. cloacae* LMG 02783. ECo, *E. coli* LMG 8224, KE, *K. aerogenes* LMG 02094, KP, *K. pneumoniae* LMG 20218, SE, *S. enterica* LMG 07233.

		Amino RES 5 (µM)				FICI
		0	2	4	8	
AB	Erythromycin	8	2	0.5	0.25	0.132
	Novobiocin	2	0.25	0.031	0.008	0.14
	Rifampicin	0.5	0.062	0.004	0.001	0.133

ECl	Erythromycin	32	32	16	16	0.625
	Nalidixic acid	10.66 ± 2.67	2	1.33 ± 0.33	1.33 ± 0.33	0.25
	Novobiocin	26.67 ± 5.33	6.67 ± 1.33	2.67 ± 0.67	1.33 ± 0.33	0.225
	Rifampicin	>32	21.33 ± 5.33	8	2	0.250

ECo	Erythromycin	32	16	2.33 ± 0.88	0.1 ± 0.02	0.286
	Nalidixic acid	8	4	1	0.080.02	0.375
	Novobiocin	42.66 ± 10.66	16	4	0.031	0.343
	Rifampicin	4	1	0.08 ± 0.02	0.05 ± 0.01	0.27

KA	Erythromycin	>32	32	13.33 ± 2.66	2	0.156
	Minocyclin	4	1	0.5	0.5	0.187
	Nalidixic acid	>32	13.33 ± 2.66	2	2	0.093
	Novobiocin	13.33 ± 2.66	4.67 ± 1.76	2.67 ± 0.66	2	0.262
	Rifampicin	21.33 ± 5.33	8	0.5	0.125	0.085

KP	Minocyclin	>32	32	10.66 ± 2.66	1.33 ± 0.33	0.145
	Nalidixic acid	>32	>32	>32	5.33 ± 1.33	0.208
	Rifampicin	32	32	16	0.42 ± 0.08	0.138

SE	Erythromycin	>32	>32	32	16	0.5
	Nalidixic acid	8	6.66 ± 1.33	4	2	0.5
	Polymyxin B	0.25	0.1 ± 0.04	<0.004	<0.004	0.133
	Rifampicin	8	8	2	0.125	0.265

For the FICI calculations, twice the highest concentration tested was used in the cases where the MIC was not reached. The FICI value for the best combination is represented. The concentrations are expressed in µM ± SE.

### Haemolytic activity and toxicity

Haemolytic activity was evaluated by monitoring haemoglobin leakage from human red blood cells as a consequence of membrane damage. HC_10_ and HC_50_ were defined as the peptide concentrations causing 10% and 50% haemolysis on erythrocytes. Amino RES derivatives were tested in human erythrocytes, at concentrations ranging from 128 to 1 µM (Supplementary Figure 3). We observed that, overall, the designed compounds showed low haemolytic activity (HC_10_, except compounds **6** and **7**) and that both, antimicrobial activity and haemolytic toxicity increased with the increase of hydrophobicity. However, a very hydrophobic structure led to decreased activity and increased haemolysis, as we found for **6** and **7**, and as previously reported for other compounds.^36^ A balance between amphipathicity and hydrophobicity has been proposed as desirable to keep the antimicrobial activity low in haemolytic activity.^36^^,^^60^^,^^61^ In this sense, amino RES **5** is the best-tested compound, the most active and low haemolytic (Supplementary Figure 3, Table 4). No haemolytic activity was observed for RES or the positive charged derivatives **2** and **8** (Table 4) and only compounds **6** and **7** reached an HC_50_ at concentrations close to the higher tested (124.43 ± 2.88 and 79.89 ± 8.42 μM respectively).^62^ Based on the data obtained, the selectivity index HC_50_/MIC was calculated (Supplementary Table 1). In the case of the more active amino RES (**5**), this index ranged between 4 to 19.2, 8 to 76.8, 4 to 32, and 12 to 128 for Gram-negative and Gram-positive aerobic bacteria, and Gram-negative and Gram-positive anaerobic bacteria, respectively (Supplementary Table 1).

**Table 4.** Haemolityc activity against human red cells and cytotoxicity of the amino RES derivatives. The concentrations are expressed in μM ± SE.

		Haemolysis		EC_50_ HTC-166
		HC_10_	HC_50_	
RES		>128	>128	59.59 ± 0.93
amino RES	**2**	>128	>128	106.5 ± 0
	**3**	107.37 ± 2.73	>128	6.90 ± 0.04
	**4**	64.10 ± 0.81	>128	8.59 ± 0.39
	**5**	92.60 ± 1.50	>128	3.90 ± 3.67
	**6**	53 ± 2.32	124.43 ± 2.88	3.29 ± 1.13
	**7**	38.30 ± 4.77	79.90 ± 8.42	3.83 ± 0.15
	**8**	>128	>128	9.48 ± 1.43

Considering the antimicrobial activity against anaerobic bacteria, a cytotoxicity assay was performed against the colon cell line HTC-166. Despite the promising data obtained in the haemolytic assay, the cytotoxicity assay showed that the most active antibacterial compounds were also toxic against this cell line (Table 4). Only compound **2** showed low toxicity, even lower than RES (Table 4).

## Conclusions

Resveratrol is a relevant natural product with a stilbene scaffold that is being used for the design of new drugs. In this study, we have designed and prepared RES derivatives trying to emulate cationic amphipathic antimicrobials. To do so, we have added amine and alkyl groups at different positions of RES, reducing the core size of cationic peptides and generating new active compounds. Although RES is poorly- or non-active as an antimicrobial agent, the new RES derivatives showed notable activity, especially against strictly anaerobic growing Gram-positive and Gram-negative bacteria. In the case of aerobic bacteria, Gram-negative bacteria showed higher resistance, while Gram-positive bacteria were most sensitive. This antimicrobial activity was somehow related to the hydrophobicity of the molecules. As expected for cationic amphipathic molecules, the targets were the bacterial membranes, where the different compounds induced a dose-related permeabilization in both the outer membrane (in Gram-negative bacteria) and the inner membrane. The different resistance observed between bacterial groups, especially between Gram-negative aerobic and anaerobic bacteria, could be related to structural differences in the outer membrane or higher sensibility to the membrane permeabilization in the case of anaerobic bacteria. This membrane-permeabilization activity is related also to the synergistic effect observed for **5** and some hydrophobic antimicrobials such as rifampicin, nalidixic acid or erythromycin. Unexpected remarkable synergisms with other hydrophilic drugs were observed in a strain-specific way. No cross-resistance was observed for the combination of **5** with other antibiotics. Finally, a haemolytic test showed the importance of the hydrophobic groups in the membrane interaction, since the presence of large alkyl groups causes a more erythrolytic effect of the derivatives (amino RES derivatives **6** and **7**) than shorter alkyl groups, or with no alkyl groups, such as for amino RES **8**. Overall, the most active compound **5** was poorly haemolytic. However, the different RES derivatives displayed certain levels of cytotoxicity against HTC-166 colonic cell lines. Our data support that RES could be used as a scaffold for the design of new active RES derivatives emulating cationic and amphipathic antimicrobial drugs but with a reduced core size in comparison to cationic peptides. The proper relation between the charge of the molecule and the hydrophobic part is essential to provide antimicrobial activity and reduced haemolytic activity. The absence of cross-resistance with traditional antibiotics and the activity observed, especially against strict anaerobic bacteria, encourages future design and preparation of new amino RES derivatives in which cytotoxicity should be reduced while keeping the antimicrobial activity.

## Experimental section

### Chemistry

All solvents and chemicals were used as purchased without further purification. All reactions were monitored by TLC on precoated silica gel 60 plates F_254_ (Merck) and detected by heating after staining with H_2_SO_4_:EtOH (1:9, *v/v*), anisaldehyde (450 ml ethanol, 25 ml anisaldehyde, 25 ml H_2_SO_4_ and 1 ml AcOH) or Mostain (500 ml of 10% H_2_SO_4_, 25 g of (NH_4_)_6_Mo_7_O_24_•4H_2_O, 1 g Ce(SO_4_)_2_•4H_2_O). Products were purified by flash chromatography with silica gel 60 (200–400 mesh). Eluents are indicated for each particular case. NMR spectra were recorded on Bruker Advance 300, 400, or 500 MHz [300, 400, or 500 MHz (^1^H), 75, 101, or 126 (^13^C)] NMR spectrometers, at room temperature for solutions in CDCl_3_, or CD_3_OD. Chemical shifts are referred to the solvent signal. 2 D experiments (COSY, TOCSY, and HMQC) were done when necessary to assign the new compounds. Chemical shifts are in ppm. Low-resolution mass spectra were obtained on an ESI/ion trap mass spectrometer. High-resolution mass spectra (HRMS) were obtained on an ESI/quadrupole mass spectrometer (WATERS, ACQUITY H CLASS). If necessary, the purity was determined by high-performance liquid chromatography (HPLC). The purity of all final compounds was 95% or higher. The instrument used for chromatographic separation was a Waters Acquity UPLC^TM^ H-class system (Waters, Manchester, UK). The column was an Acquity UPLC^R^ BEH C18 (2.1 × 100 mm, 1.7 µM). A QDA single quadrupole mass spectrometer (Waters) equipped with an orthogonal Z-spray^TM^ electrospray ionisation (ESI) source was used for metabolites detection. Empower 3 software was used for instrument control, peak detection, and integration.

### General procedure for alkylation a with bromoalkanes

Resveratrol (1 eq.) and potassium carbonate (2 eq.) were added to DMF (2.85 ml/mmol of resveratrol) under agitation in a round-bottomed flask. 1-bromoalkane (1–1.5 eq) was added dropwise and the reaction was stirred for 6 h at room temperature. The reaction mixture was filtered, diluted with water and extracted with ethyl acetate (3 × 30 ml). The combined organic layers were dried with MgSO_4_ and the mixture was filtered, concentrated. The crude was purified by flash column chromatography using different hexane/ethyl acetate mixtures.

### General procedure for alkylation B with bromoalkanes followed by azide formation

Resveratrol derivatives (1 eq.) and potassium carbonate (3 eq.) were dissolved in dry DMF (8–10 ml). 1-Bromo-3-cloropropane (3–6 eq.) was then added and the reaction was then stirred at 80 °C for 6 h. The reaction mixture was diluted with water and extracted with ethyl acetate (2 × 40 ml). The combined organic layers were dried with MgSO_4_ and the mixture was filtered and concentrated. The crude was used for the next step without any further purification. The latter was dissolved in dry DMF and NaN_3_ (7 eq.) and the mixture was stirred at 70 °C for 8 h. The reaction mixture was diluted with water and extracted with ethyl acetate (2 × 40 ml). The combined organic layers were dried with MgSO_4_ and the mixture was filtered and concentrated, and the crude was purified by flash column chromatography using different hexane/ethyl acetate mixtures from 20:1 to 5:1.

### General procedure C for reduction with a phosphine

Resveratrol derivatives with azido groups (1 eq.) and PPh_3_ (3–6 eq.) were dissolved in dry THF (8–10 ml). The reaction mixture was stirred for 16 h and the solvent was then removed. The latter crude was then purified either by Sephadex LH 20 eluting with MeOH or CH_2_Cl_2_:MeOH (1:2). Fractions containing the desired product were concentrated affording the amino resveratrol derivative.

#### Tert-but©(E)-(3–(3-((triisopropylsilyl)oxy)-5–(4-triisopropylsilyl) oxy)styryl)phenoxy)propyl) carbamate (10)

Resveratrol derivative **9** (500 mg, 0.92 mmol) and the linker amino tert-butyl (2-iodopropyl)carbamate^30^ (790 mg, 2.77 mmol, 3.0 eq) were dissolved in dry DMF (8 ml). K_2_CO_3_ previously activated was then added to the solution. The reaction mixture was stirred at room temperature for 3 h and a saturated solution of NH_4_Cl was then added to neutralise (50 ml). The organic phase was extracted with ethyl acetate (2 × 50 ml) and washed with H_2_O (50 ml). The resulting residue was purified by flash column chromatography (hexane: ethyl acetate from 10:1 to 9:1) to afford **10** (266 mg, 42%) as a yellow glassy solid; δ_H_ (400 MHz, CDCl_3_) 7.40 (m, 2 H, Harom), 7.00 (d, 1 H, J = 16.2 Hz, =CH), 6.90–6.86 (m, 3 H, 2 Harom, 1x = CH), 6.66–6.63 (m, 2 H, Harom), 6.36 (t, 1 H, J = 2.1 Hz, Harom), 4.97–4.04 (t, 2 H, CH2O), 3.40–3.34 (m, 2H, CH_2_NH), 2.04–1.97 (m, 2H, CH2), 1.48 (s, 9H,C(CH_3_)_3_), 1.34–1.25 (m, 6 H, CH(CH_3_)_2_), 1.16, 1.15, 1.14, 1.13 (4 s, 36 H, CH(CH_3_)_2_); δ_C_ (100 MHz, CDCl_3_) 159.9 (C = O), 157.2, 156.0, 155.9, 139.5, 130.2, 128.6, 127.7, 126.6, 120.1, 110.9, 105.7, 105.0, 65.9, 38.1, 29.5, 28.4, 18.0, 17.9, 12.7); (HRMS (ES+) Calcd. for C_40_H_67_NO_5_Si_2_Na (M+) 720.4455, found: 720.4458.

#### (E)-3–(3-aminopropoxy)-5–(4-hydroxystyryl)phenol (2)

Trifluoroacetic acid (8 ml) was added to a solution of compound **10** (200 mg, 0.288 mmol) in a mixture of THF:H_2_O (1:1, 2 ml). The reaction mixture was stirred at room temperature for 24 h. Then, the solvents were removed and the mixture was co-evaporated with toluene. The residue was purified by Sephadex G50 (MeOH:H_2_O 4:1) to afford compound **2** as a yellow oil (75 mg, 92%). Overall yield for **2** from RES (**1**) was 10%. δ_H_ (400 MHz, CD_3_OD) 7.40 (d, 2 H, Harom), 6.92 (d, 1H, =CH), 6.82–6.72 (m, 4 H, 3 Harom, 1x = CH), 6.50 (br.s, 1 H, Harom), 6,27 (br.s, 1 H, Harom), 4.04 (t, 2H, CH2O), 2.68 (t, 2H, CH_2_NH), 1.98 (t, 2H, CH_2_); δ_C_ (100 MHz, CDCl_3_) 159.9 (C = O), 158.4, 157.1, 155.7, 140.1, 130.2, 128.6, 127.7, 126.6, 120.1, 110.9, 105.7, 105.0, 64.7, 37.4, 27.2; (HRMS (ES+) Calcd. for C_17_H_20_NO_3_ (M+) 286.1443, found: 286.1442.

#### (E)-1–(3-butoxy-5-hydroxyphenyl)-2–(4’-butoxyphenyl)ethene (12) compound characterisation in accordance to literatu©^31^

##### (E)-5–(4-(octyloxy)styryl)benzene-1,3-diol (13)

Following the general procedure A and starting from resveratrol (2 g, 8.76 mmol, 1 eq.) and 1-bromooctane (2.3 ml, 1.5 eq) the reaction yielded a mixture of octyl resveratrol derivatives. After purification with flash chromatography whilst eluting with a gradient concentration of hexane:ethyl acetate (10:1 to 1:1), the desired compound **13** (17%) was isolated as an orange oil and used to complete the synthesis. ^1^H NMR (300 MHz, CD_3_OD) d 7.38 (d, J 8.4 Hz, 2H), 7.00 (d, J 16.3 Hz, 1H), 6.85 (d, J 16.3 Hz, 1H), 6.80 (d, J 8.4 Hz, 2H), 6.58 (s, 1H), 6.56 (s, 1H), 6.28 (s, 1H), 3.88 (t, J 6.4 Hz, 2H), 1.80–1.60 (m, 2H), 1.47–1.22 (m, 10 H), 0.88 (t, J 6.9 Hz, 3H). ^13^C NMR (75 MHz, CD_3_OD) d 158.9, 158.2, 139.8, 129.9, 127.8, 127.3, 126.3, 114.3, 104.5, 101.4, 67.731.7, 29.42, 29.40, 29.36, 29.16, 29.12, 29.04, 25.79, 22.36, 13.1. ESI-HRMS calcd for C_22_H_29_O_3_ 341.2117, found 341.2106.

##### (E)-5–(4-(decyloxy)styryl)benzene-1,3-diol (14)

Following the general procedure A and starting from resveratrol (2 g, 8,76 mmol, 1 eq.) and 1-bromodecane (3,5 ml, 1.5 eq) the reaction yielded a mixture of decyl resveratrol derivatives. After purification with flash chromatography whilst eluting with a gradient concentration of hexane:ethyl acetate (10:1 to 1:1), the desired compound **14** (19%) was isolated as an orange oil and used to complete the synthesis. ^1^H NMR (300 MHz, CD_3_OD) d 7.41 (d, J 8.4 Hz, 2H), 6.98 (d, J 16.2 Hz, 1H), 6.88–6.81 (m, 3H), 6.48 (d, J 2.1 Hz, 1H), 6.20 (t, J 2.1 Hz, 1H), 3.94 (t, J 6.4 Hz, 2H), 1.79–1.70 (m, 2H), 1.48–1.29 (m, 14 H), 0.90 (t, J 6.9 Hz, 3H). ^13^C NMR (75 MHz, CD_3_OD) 158.9, 158.3, 139.8, 129.9, 127.8, 127.3, 126.3, 114.3, 104.5, 101.4, 67.7, 31.7, 29.42, 29.40, 29.36, 29.16, 29.12, 29.04, 25.79, 22.36, 13.1. ESI-HRMS calcd for C_24_H_32_O_3_ 368.2351, found 368.2364.

##### te©Butyl (E)-(3–(3-butoxy-5–(4-butoxystyryl)phenoxy)propyl)carbamate (15)

Resveratrol derivative **12** (250 mg, 0.734 mmol) and the linker amino tert-butyl (3-iodopropyl)carbamate (314 mg, 1.1 mmol, 1.5 eq) were dissolved in dry DMF (5 ml). K_2_CO_3_ (152 mg, 1.1 mmol) previously activated was then added to the solution. The reaction mixture was stirred at room temperature for 3 h and a saturated solution of NH_4_Cl was then added to neutralise (50 ml). The organic phase was extracted with ethyl acetate (2 × 50 ml) and washed with H_2_O (50 ml). The resulting residue was purified by flash column chromatography (hexane: ethyl acetate from 10:1 to 9:1) to afford **15** (248 mg, 68%) as a yellow glassy solid; δ_H_ (400 MHz, CDCl_3_) ^1^H NMR (400 MHz, CD_3_OD) δ = 7.42 (d, J = 6.5 Hz, 2H), 7.01 (d, J = 12.2 Hz, 1H), 6.89–6,85 (m, 3H), 6.64–6.62 (m, 2H), 6.36 (t, J = 1,6 Hz, 1H), 4.04 (t, J = 6.4 Hz, 2H), 3.97 (t, 4H, CH_2_O), 3.35–3.29 (m, 2H, CH_2_N), 2.03–1.97 (m– 6H), 1.81 − 1.74 (m, 2H), 1.42 (s, 9H), 1.03–0.97 (m, 6H). ^13^C NMR (101 MHz, CD_3_OD) δ = 160.5, 160.1, 156.0, 155.9, 139.6, 129.7, 128.7, 127.7, 126.4, 114.7, 105.1, 104.7, 100.5, 67.7, 33.4, 31.3, 28.4, 19.2, 13.9. ESI-HRMS [M + H] calcd for C_30_H_43_NaNO_5_ 520.3039, found 520.3118.©*rt-Butyl (E)-(2–(3-butoxy-5–(4-butoxystyryl)phenoxy)ethyl)carbamate* (**16**).

Resveratrol derivative **12** (250 mg, 0.734 mmol) and the linker amino tert-butyl (2-bromoethyl)carbamate (247 mg, 1.1 mmol, 1.5 eq) were dissolved in dry DMF (5 ml). K_2_CO_3_ (152 mg, 1.1 mmol) previously activated was then added to the solution. The reaction mixture was stirred at room temperature for 3 h and a saturated solution of NH_4_Cl was then added to neutralise (50 ml). The organic phase was extracted with ethyl acetate (2 × 50 ml) and washed with H_2_O (50 ml). The resulting residue was purified by flash column chromatography (hexane: ethyl acetate from 10:1 to 9:1) to afford **16** (280 mg, 79%) as a yellow glassy solid; δ_H_ (400 MHz, CDCl_3_) ^1^H NMR (400 MHz, CD_3_OD) δ = 7.45 (d, J = 6.5 Hz, 2H), 7.06 (d, J = 12.2 Hz, 1H), 6.92–6,87 (m, 3H), 6.68–6.65 (m, 2H), 6.38 (t, J = 1,6 Hz, 1H), 4.06 (t, J = 6.4 Hz, 2H), 4.00 (t, 2H, CH_2_O), 3.49–3.45 (m, 2–, CH_2_N), 1.82 − 1.78 (m, 4H), 1.55–1.48 (m, 13H), 1.03–0.98 (m, 6H). ^13^C NMR (101 MHz, CD_3_OD) δ = 160.5, 159.9, 159.0, 139.8, 129.7, 128.8, 127.7, 126.3, 114.7, 105.4, 104.6, 100.5, 79.5, 67.8, 67.2, 31.3, 28.4, 19.2, 13.9. ESI-HRMS [M + H] calcd for C_29_H_41_NaNO_5_ 506.2877, f©d 506.2862.

##### (E)-2–(3-butoxy-5–(4-butoxystyryl)phenoxy)propane-1-amine (3)

A solution of compound **15** (280 mg, 0.60 mmol) was dissolved in THF (5 ml) and trifluoroacetic acid (2 ml) was then added. The reaction mixture was stirred at room temperature for 24 h. After this time, solvents were removed and the mixture was co evaporated with toluene. The resulting residue was purified by flash column chromatography (ethyl acetate: methanol from 1:0 to 5:1) to afford **3** (218 mg, 98%) %) as a brown oil. Overall yield for **3** from RES (**1**) was 8.6%. δ_H_ (300 MHz, CD_3_OD) 7.40 (d, 2 H, Harom), 7.04 (d, 1H, =CH), 6.90–6.84 (m, 4 H, 3 Harom, 1x = CH), 6.68–6.55 (m, 1 H, Harom), 6.40 (br.s, 1 H, Harom), 3.95–3.90 (m, 4H), 3.27 (t, 2H), 3.03 (t, 2H), 2.19–2.13 (m, 4H), 2.41 (t, 2H), 1.73–1.68 (m, 4H), 1.51–1.46 (m, 4H), 1.23 (t, 2H), 1.00–0.95 (m, 6H); δ_C_ (75 MHz, CD_3_OD) 160.5, 159.8, 158.9, 139.8, 129.7, 128.4, 127.5, 125.9, 118.3, 115.5, 114.3, 105.1, 104.3, 100.2, 67.4, 67.3, 40.0, 37.2, 31.2, 31.1, 30.8, 27.1, 18.9, 12.9; (HRMS (ES^+^) Calcd. for C_25_H_36_NO_3_ (M^+^) 398.2690,©und: 398.2677.

##### (E)-3–(3-butoxy-5–(4-butoxystyryl)phenoxy)ethane-1-amine (4)

A solution of compound **16** (280 mg, 0.734 mmol) was dissolved in THF (5 ml) and trifluoroacetic acid (2 ml) was then added. The reaction mixture was stirred at room temperature for 24 h. After this time, solvents were removed and the mixture was co-evaporated with toluene. The residue was purified by Sephadex LH20 eluting with methanol and fractions containing the desired product were concentrated to afford the compound **4** (280 mg, 98%) %) as a yellow oil. Overall yield for **4** from RES (**1**) was 10%. δ_H_ (300 MHz, CD_3_OD) 7.43 (d, 2 H, Harom), 7.07 (d, 1H, =CH), 6.92–6.85 (m, 4 H, 3 Harom, 1x = CH), 6.74–6.70 (m, 1 H, Harom), 6.45 (br.s, 1 H, Harom), 4.24–4.21 (t, 2H, CH_2_O), 3.98–3.93 (m, 4H), 3.67 (t, 2H), 3.42–3.33 (m, 4H), 1.76–1.70 (m, 2H), 1.53–1.45 (m, 4H), 1.00–0.95 (m, 6H); δ_C_ (75 MHz, CD_3_OD) 160.5, 159.4, 159.0, 139.9, 129.7, 128.6, 127.5, 125.8, 114.3, 105.6, 104.2, 100.4, 67.4, 67.3, 63.9, 41.0, 38.9, 31.1, 26.9, 19.6, 13.1. (HRMS (ES+) Calcd. for C_24_H_34_NO_3_ (M^+^) 384.2533, found: 384.2521.

##### (E)-1,3-bis(3-azidopropoxy)-5–(4-butoxystyryl)benzene (17)

Following the general procedure **B** and starting from resveratrol derivative **11** (329 mg, 1.15 mmol) and 1-bromo-3-chloropropane (0.34 ml, 3.45 mmol), the reaction yielded compound **17** (450 mg, 87%) after purification with flash chromatography whilst eluting with a gradient concentration of hexane:ethyl acetate (20:1 to 5:1)%) as a yellow glassy solid. ^1^H NMR (300 MHz, CDCl_3_) d 7.42 (d, J 8.07 Hz, 2H), 7.00 (d, J 16.2 Hz, 1H), 6.90–6.84 (m, 3H), 6.64–6.2 (d, 2H), 6.36 (t, J 2.04 Hz, 1H), 4.07–4.02 (t, 4H), 3.97 (t, J 6.5 Hz, 2H), 3.52–3.48 (m, 4H), 2.08–1.99 (m, 4H), 1.79–1.72 (m, 2H), 1.53–1.46 (m, 2 H), 0.98 (t, J 7.32 Hz, 3H). ^13^C NMR (75 MHz, CDCl_3_) δ 160.5, 160.0, 158.5, 139.6, 130.2, 128.6, 127.8, 126.7, 114.7, 105.3, 104.8, 100.6, 67.8, 64.6, 48.3, 48.2, 31.4, 28.9, 28.8, 19.3, 13.9. ESI-HRMS calcd for C_24_H_30_N_6_NaO_3_ 473.2777, found 473.2768.

##### (E)-1,3-bis(3-azidopropoxy)-5–(4-(octyloxy)styryl)benzene (18)

Following the general procedure **B** and starting from compound **13** (490 mg, 1.44 mmol) and 1-bromo-3-chloropropane (0.42 ml, 4.32 mmol), the reaction yielded compound **18** (154 mg, 21%) as a yellow solid, after purification with flash chromatography whilst eluting with a gradient concentration of hexane:ethyl acetate (15:1 to 5:1). ^1^H NMR (300 MHz, CDCl_3_) d 7.41 (d, J 8.7 Hz, 2H), 7.00 (d, J 16.2 Hz, 1H), 6.89–6.84 (m, 3H), 6.64 (d, J 2.1 Hz, 2H), 6.35 (t, J 2.4 Hz, 1H), 4.06 (t, J 6.0 Hz, 4H), 3.96 (t, J 6.6 Hz, 2H), 3.52 (t, J 6.6 Hz, 4H), 2.05 (q, J 6.4 Hz, 4H), 1.82–1.73 (m, 2H), 1.47–1.29 (m, 10 H), 0.90 (t, J 6.6 Hz, 3H). ^13^C NMR (75 MHz, CDCl_3_) δ 160.0, 159.0, 139.9, 129.6, 129.0, 127.8, 126.2, 114.7, 105.1, 100.6, 68.1, 64.6, 48.3, 31.8, 29.38, 29.28, 29.26, 28.82, 26.06, 22.68, 14.1. ESI-HRMS calcd for C_28_H_38_N_6_NaO_3_ 529.2903, found 529.2898.

##### (E)-1,3-bis(3-azidopropoxy)-5–(4-(decyloxy)styryl)benzene (19)

Following the general procedure **B** and starting from compound **14** (500 mg, 1.35 mmol) and 1-bromo-3-chloropropane (0.80 ml, 8.14 mmol), the reaction yielded compound **19** (180 mg, 25%) as a yellow solid, after purification with flash chromatography whilst eluting with a gradient concentration of hexane:ethyl acetate (15:1 to 5:1).mmol) was then added and the reaction was then stirred at 80 °C for 6 h. The reaction mixture was diluted with water and extracted with ethylacetate (2 × 50 ml). ^1^H NMR (300 MHz, CDCl_3_) d 7.42 (d, J 8.7 Hz, 2H), 6.99 (d, J 16.2 Hz, 1H), 6.89–6.84 (m, 3H), 6.64 (d, 2H, J = 2.1 Hz), 6.35 (t, 1H, J = 6.6 Hz), 4.06 (t, J 6.0 Hz, 4H), 3.96 (t, J 6.6 Hz, 2H), 2.96–2.82 (m, 4H), 2.05 (q, J 6.4 Hz, 4H), 1.82–1.173 (m, 2H), 1.47–1.29 (m, 12 H), 0.89 (t, J 6.6 Hz, 3H). ^13^C NMR (75 MHz, CDCl_3_): δ 160.0, 159.1, 139.9, 129.6, 129.0, 127.8, 126.2, 114.7, 105.1, 100.6, 68.1, 64.6, 48.3, 31.8, 29.38, 29.29, 29.26, 28.82, 26.06, 22.68, 14.1. ESI-HRMS calcd for C_30_H_42_N_6_NaO_3_ 557.3216, found 529.2898.

##### (E)-1,3-bis(3-azidopropoxy)-5–(4-(3-azidopropoxy)styryl)benzene (20)

Following the general procedure **B** and starting from resveratrol **1** (300 mg, 1.3 mmol) and 1-bromo-3-chloropropane (0.78 ml, 7.8 mmol), the reaction yielded compound **20** (480 mg, 78%) as a yellow oil, after purification with flash chromatography whilst eluting with a gradient concentration of hexane:ethyl acetate (15:1 to 5:1). ^1^H NMR (300 MHz, CDCl_3_); δ 7.43 (d, 2 H, J = 7.0 Hz, Harom), 7.03 (d, 1 H, J = 16.2 Hz, =CH), 6.90–6.85 (m, 3 H, 2 Harom, 1x = CH), 6.64–6.63 (m, 2 H, Harom), 6.36 (t, 1 H, J = 2.1 Hz, Harom), 4.07 (t, 6H, J = 6.0 Hz, CH_2_O), 3.52 (t, 6H, J = 6.0 Hz, CH_2_N_3_), 2.1–2.02 (t, 6H, J = 6.0 Hz, -CH_2_-); δ_C_ (75 MHz, CDCl_3_) 158.5, 139.8, 130.1, 128.8, 127.8, 126.5, 114.7, 105.1, 100.6, 64.6, 48.2, 28.8; (HRMS (ES+) calc. for C_23_H_28_N_3_O_3_ (M^+^) 478.2315, fo’nd 478.2309.

##### *(E)-3,3'-((5–(4-butoxystyryl)-1,3-phenylene)bis(oxy))bis(propan-1-amine)* (5)

Following the general procedure C and starting from compound **17** (192 mg, 0.426 mmol) and PPh_3_ (180 mg, 1.27 mmol), the reaction yielded compound **5** (142 mg, 83%), as a yellow oil. Overall yield for **5** from RES (**1**) was 25%. ^1^H NMR (300 MHz, CDCl_3_): δ 7.55–7.49 (m, 4H), 7.18 (d, J 8.07 Hz, 2H), 7.02–6.92 (m, 3 H), 6.74–6.71 (m, 2H), 6.38 (t, J 2.04 Hz, 1H), 4.06–4.02 (t, 4H), 3.99–3.95 (t, 2H), 2.90–2.78 (m, 4H), 2.72–2.69 (m, 4H), 1.83–1.77 (m, 2H), 1.48–1.40 (m, 2 H), 0.98 (t, J 7.32 Hz, 3H). ^13^C NMR (75 MHz, CDCl_3_) δ 160.5, 158.9, 139.8, 131.7, 130.2, 130.1, 129.9, 129.0, 128.9, 128.8, 128.2, 126.6, 115.1, 105.1, 100.7, 67.6, 66.0, 33.1, 31.3, 19.2, 18.6, 17.7, 14.2. ESI-HRMS calcd for C_24_H_35_N_3_O_3_ 399.2642, fo’nd 399.2643.

##### (E)-3,3'-((5–(4-(octyloxy)styryl)-1,3-phenylene)bis(oxy))bis(propan-1-amine) (6)

Following the general procedure C, and starting from compound **18** (120 mg, 0.24 mmol) and PPh_3_ (310 mg, 1.18 mmol), after purification by Sephadex, fractions containing the amino derivate were concentrated to afford compound **6** (88 mg, 83%) as a yellow oil. Overall yield for **6** from RES (**1**) was 2.9%. ^1^H NMR (300 MHz, MeOD and some drops of CDCl_3_) d 7.42 (d, J 8.7 Hz, 2H), 7.04 (d, J 16.2 Hz, 1H), 6.92–6.85 (m, 3H), 6.66 (s, 2H), 6.38 (s, 1H), 4.06 (t, J 6.0 Hz, 4H), 3.95 (t, J 6.6 Hz, 2H), 3.40–3.30 (m, 4H), 1.95 (q, J 6.4 Hz, 4H), 1.81–1.70 (m, 2H), 1.50–1.26 (m, 10 H), 0.90 (t, J 6.6 Hz, 3H). ^13^C NMR (75 MHz, CDCl_3_) δ 160.3, 159.0, 139.9, 131.8, 131.6, 129.8, 128.7, 128.5, 128.4, 127.5, 126.0, 114.4, 104.6, 100.3, 67.7, 65.7, 38.4, 31.6, 29.14, 29.1, 25.8, 22.3, 13.1. ESI-HRMS calcd for C_28_H_43_N_2_O_3_ 455.3274, fo’nd 455.3257.

##### (E)-3,3'-((5–(4-(decyloxy)styryl)-1,3-phenylene)bis(oxy))bis(propan-1-amine) (7)

Following the general procedure C, and starting from compound **19** (180 mg, 0.355 mmol) and PPh_3_ (467 mg, 1.77 mmol), after purification by Sephadex, fractions containing the amino derivate were concentrated to afford compound **7** (147 mg, 91%) as a yellow oil. Overall yield for **7** from RES (**1**) was 4.3%. ^1^H NMR (300 MHz, MeOD and some drops of CDCl_3_): δ 7.43 (d, J 8.7 Hz, 2H), 7.06 (d, J 16.2 Hz, 1H), 6.93–6.86 (m, 3H), 6.67 (d, J 2.1 Hz, 2H), 6.38 (t, J 2.4 Hz, 1H), 4.06 (t, J 6.0 Hz, 4H), 3.96 (t, J 6.6 Hz, 2H), 2.95–2.82 (m, 4H), 1.96 (q, J 6.4 Hz, 4H), 1.77–1.70 (m, 2H), 1.45–1.26 (m, 14 H), 0.91 (t, J 6.6 Hz, 3H). ^13^C NMR (75 MHz, CDCl_3_) δ 160.2, 159.0, 139.8, 129.7, 128.5, 127.5, 126.1, 114.4, 104.7, 100.3, 67.7, 65.7, 38.3, 31.7, 31.3, 29.44, 29.42, 29.38, 29.18, 29.14, 29.07, 25.8, 22.4, 13.3. ESI-HRMS calcd for C_30_H_47_N_2_O_3_ 483.3587, fo’nd 483.3604.

##### (E)-3,3'-((5–(4-(3-aminopropoxy)styryl)-1,3-phenylene)bis(oxy))bis(propan-1-amine) (8)

Following the general procedure C, and starting from compound **20** (306 mg, 0.64 mmol) and PPh_3_ (1 gr, 3.8 mmol), after purification by Sephadex LH 20 firstly and the product was then purified by RP chromatography eluting with (MeOH: H_2_O from 7: 1 to 0: 1). Finally, fractions containing the amino derivate were concentrated to afford compound **8** (184 mg, 72%) as a brown oil. Overall yield for **8** from RES (**1**) was 57%. ^1^H NMR (300 MHz, CDCl_3_) δ_H_ 7.8 (d, 2 H, J = 7.0 Hz, Harom), 7.02 (d, 2H, Harom), 6.92(d, 1H, J = 16.2 Hz), 6.84 (m, 3 H, Harom, CH), 6.36 (s, 1 H, Harom), 4.04 (t, 6H, J = 6.0 Hz, CH_2_O), 2.7 (t, 6H, J = 6.0 Hz, CH_2_NH_2_), 1.98 (t, 6H, J = 6.0 Hz, -CH_2_-); δ_C_ (75 MHz, CDCl_3_) 158.8, 144.5, 139.7, 129.8, 129.1, 127.4, 114.3, 1,4.9, 99.8, 72.5, 49.8, 31.0. ESI-HRMS calcd for C_23_H_36_N_3_O_3_ (M^+^) 402.2757, found 400.2732.

### Bacterial strains, culture conditions, and reagents

Gram-negative aerobic bacteria *Acinetobacter baumannii* LMG 0104, *Klebsiella aerogenes* LMG 02094, *K. pneumoniae* LMG 20218, *E. cloacae* LMG 02783, *Escherichia coli* LMG 8224, *E. coli* NCTC 13846, *Pseudomonas aeruginosa* PAO1, and *Salmonella enterica* LMG 07233 were grown on Mueller-Hinton broth (MHB, BD Difco, Franklin Lakes, NJ, USA) at 37 °C with shacking (250 rpm) while the Gram-positive bacteria *Bacillus cereus* ATCC 10987, *B. cereus* ATCC 14574, *Enterococcus faecalis* V583^63^, *E. faecalis* LMG 8222, *E. faecalis* LMG 16716, *E. faecium* LMG 11423, *E. faecium* LMG 16003, *S. aureus* LMG 15975, *S. aureus* LMG 8223, *S. aureus* LMG 10147 were grown in the same conditions, but statically. Anaerobic Gram-negative *Bacteroides ovatus* 3_8_47FAA, *B. fragilis* NCTC9343, *B. salyersiae* DSM18765, *B. xylanisolvens* DSM1883, and *Parabacteroides merdae* CL03T12C32, as well as the Gram-positive *Clostridium botulinum* CECT 551, *C. perfringens* CECT 376, *C. tetani* CECT 462 and *Clostridioides difficile* CECT 531, were grown in Gifu Anaerobic Broth (GAM, HiMedia Laboratories, Thane West, Maharashtra, India) and Reinforced Clostridial Medium (RCM, Sigma-Aldrich, St Louis, MO, USA) respectively at 37 °C in anaerobiosis using a Coy Lab’s Vinyl Anaerobic Chamber. Agar at 1.5% was added when necessary for solid medium. LMG strains were obtained from the Belgium Coordinated Collections of Microorganisms. ATCC strains from the American Type Culture Collection, CECT from the Spanish Type Culture Collection, NCTC strains from the National Collection of Type Cultures (UK), and DSM from the German Collection of Microorganisms and Cell Cultures. *Bacteroides ovatus* 3_8_47FAA and *Parabacteroides merdae* CL03T12C32 were obtained from BEI Resources (https://www.beiresources.org/).

The antibiotic tested in the synergy assay, amikacin, ampicillin, azithromycin, aztreonam, bacitracin, cefepime, chloramphenicol, ciproflocinetobacthromycin, fosfomycin, fusidic acid, gentamicin, kanamycin, meropenem, minocycline, nalidixic acid, novobiocin, oxacillin, pentamidine, polymyxin B, rifampicin, streptomycin, tetracycline, trimethoprim, and vancomycin were purchased from Sigma-Aldrich (St Louis, MO, USA) and prepared at a store concentration of 3.2 mM according to suppliers.

### Determination of the minimal inhibitory concentration test (MIC) and synergistic testing

The different amino RES derivatives were prepared at 10 mm as a stock solution in DMSO. MIC determinations for the newly designed RES derivatives (from 128 to 1 µM) and antibiotics (from 32 to 0.031 µM) were performed in MHB by the broth microdilution method according to the Clinical and Laboratory Standards Institute (CLSI) guideline for aerobic bacteria^64^. In the case of anaerobic bacteria, a similar protocol was performed but using GAM and RCM as culture mediums. Polymyxin B and daptomycin were used as positive controls for Gram-negative and Gram-positive bacteria respectively. For daptomycin MIC tests, the culture mediums were supplemented with 50 µg/mL of CaCl_2_ to get the antimicrobial activity^11^.

For the synergistic test, a broad test was initially performed ^53^. Briefly, after the first MIC test for the antibiotics and considering these data, a new MIC test was carried out in the presence of a sub-MIC concentration (0.25x MIC) of amino RES **5** to ensure positive results in the case of synergism. The Gram-negative bacteria *A. baumannii* LMG 0104, *K. aerogenes* LMG 02094, *K. pneumoniae* LMG 20218, *E. cloacae* LMG 02783, *E. coli* LMG 8224, *P. aeruginosa* PAO1, *S. enterica* LMG 07233 and the Gram-positive *E. faecalis* V583, *E. faecium* LMG 16003 and *S. aureus* LMG 8223. Those antibiotics that showed a lower MIC in the presence of amino RES **5** were selected for a checkerboard test, determining the MIC for these antibiotics at different sub-MIC of amino RES **5** (8, 4, 2, 1, 0.5 µM) and the Fractional Inhibitory Concentration Index (FICI) was calculated and interpreted according to the European Committee on Antimicrobial Susceptibility Testing (EUCAST) ^53^^,^^59^. The test was performed in triplicate and the results were analysed using GraphPad Prism software (GraphPad Software Inc., La Jolla, CA, USA).

### Activity of the amino RES in the bacterial membranes

The integrity of the membranes (outer or/and inner) was performed as previously reported^41^. Outer membrane permeabilization by the amino RES was analysed using the fluorescent probe 1-N-phenylnaphthylamine (NPN, Sigma-Aldrich). For that, *E. coli* LMG 8224 was cultured overnight in MHB, diluted 1: 100 into fresh MHB, and cultured at 37 °C with shacking (250 rpm) to the late log phase (OD_600_ = 1). The cells were washed 3 times with 5 mM HEPES buffer containing 5 mM glucose (GHEPES) and the bacterial suspension standardised to an OD_600_ = 0.5 in the same buffer. NPN was added at a final concentration of 30 μM and the amino RES at 16, 32, 64, and 128 µM, and the cells were incubated for 1 h at room temperature in dark. The fluorescence was measured at excitation/emission of 350/420 nm with a luminometer (Varioskan Flash; Thermo Scientific). Polymyxin B at 4 µm treatment was used as a positive control. The effect of LPS (0–256 µM, the major component of the outer membrane), Mg^2+,^ and Ca^2+^ (0–32 mM, divalent cations are involved in an increase of the stability of the outer membrane and they are essential for its integrity) on the activity of amino RES **5** was tested. For that, a MIC test was performed in the presence of different concentrations of these compounds^40^^,^^41^.

In the case of inner membrane permeabilization once the cells of *E. coli* LMG 8224 and *S. aureus* LMG 8223 were prepared as before, and after that, they were washed three times in PBS and adjusted to an OD_600_ =0.5. Next, propidium iodide (Sigma-Aldrich) at 1 µM was added in the presence of 16, 32, 64, and 128 µM of amino RES. The cells were incubated for 1 h in dark and the fluorescence was monitored at an excitation/emission wavelength of 535/615 nm with a luminometer (Varioskan Flash; Thermo Scientific). polymyxin B and gramicidin S at 4 µM were used as positive controls for Gram-negative and Gram-positive bacteria respectively. All the tests were performed in triplicate.

For the membrane potential assay, bacteria were cultured at 37 °C in CA-MHB medium to an OD_600_ of 1. After that, the cells were washed three times with 5 mM GHEPES buffer and bacterial suspensions to an OD_600_ = 0.5 were prepared. 3,3-Dipropylthiadicarbocyanine iodide DiSC_3_(5) was added to the cells at a final concentration of 2 μM. Amino RES 5 was added into the bacterial suspension at 4, 8, 16, and 32 µM using RES at 32 µM and polymyxin B and gramicidin S at 4 µM as controls. The fluorescence was monitored at excitation/emission of 622/670 nm every 5 min for 1 h with a luminometer (Varioskan Flash; Thermo Scientific). SDS (1%). All the tests were performed in triplicate.

Finally, for the determination of the bactericidal or bacteriostatic mode of action a regular MIC test from 128 to 1 µM using amino RES **5** was performed with *E. coli* LMG 8224 and *B. cereus* ATCC 10987, and after that, the remaining cells were used for reinoculated at 10% new fresh MHB medium. the cells were incubated for 24 h at 37 °C. The absence of growth after this time at the MIC concentration or close was indicative of a bactericidal effect ^65^.

### Haemolytic activity and cytotoxicity

For the haemolytic test, human blood of healthy individuals was obtained from Sanquin (certified Dutch organisation responsible for meeting the need in healthcare for blood and blood products, https://www.sanquin.nl/) and the erythrocytes were prepared as described ^40^^,^^66^. 5 ml of red cells were centrifugated at 1000 x g for 5 min at room temperature. The supernatant was discarded and the pellets were rinsed three times in saline solution (0.9% NaCl). Finally, the cells were resuspended in 5 mL of saline solution. Erythrocytes were diluted 10 fold and to this suspension were added different concentrations (1, 2, 4, 8, 16, 32, 64, and 128 µM) of the amino RES samples. Mixtures were maintained at 37 °C for 1 h with slow stirring. Next, the mixtures were centrifuged in the same conditions, and the haemoglobin release was quantitated by measuring optical density at 540 nm. Positive control (100% haemolysis) was obtained by adding 2% of triton X-100. The percentage of haemolysis (% H) was calculated as follows: % H = 100 × (A − A0)/Atot, with A being the absorbance of the sample with added AS-48; A0, the absorbance of the negative control (0.9% NaCl); and Atot, the absorbance of the positive control. 10% and 50% haemolysis parameters (HC_10_ and HC_50_) were extrapolated from the haemolysis data using GraphPad Prism 7. All the experiments were performed in triplicate.

The colon cell line HTC-166 was used in the cytotoxicity assay. Cells were cultivated in Dulbecco’s Modified Eagle Medium high glucose (DMEM) supplemented with 10% heat-inactivated foetal bovine serum, 1 mM sodium pyruvate, 1x MEM non-essential amino acids, 2 mM glutamine, 100 u/mL penicillin and 100 mg/mL streptomycin and maintained at 37 °C, 5% of CO_2_ and 100% of humidity in 75 cm^2^ cell culture flasks. For the cytotoxicity test, the cells were harvested by trypsinization (0.25%) and seeded in 96 well plates (10.000 cells/well), and incubated as before for 24 h for the cell adhesion to the plate. After this time the supernatant was removed and a new medium was added. Amino RES were also added at concentrations ranging from 1 to 128 µM and the cells were incubated for 72 h. After this time, cytotoxicity was determined using the colourimetric MTT-based assay ^67^. The concentration of compounds that reduced the cell growth by 50% with respect to the negative control (EC_50_) was calculated using GraphPad Prism 7 program.

## Supplementary Material

Supplemental Material

## References

[1] Inagaki K, Lucar J, Blackshear C, Hobbs CV. Methicillin-susceptible and methicillin-resistant staphylococcus aureus bacteremia: nationwide estimates of 30-day readmission, in-hospital mortality, length of stay, and cost in the United States. Clin Infect Dis. 2019;69 (12):2112–2118. 30753447 10.1093/cid/ciz123

[2] Theuretzbacher U, Bush K, Harbarth S, Paul M, Rex JH, Tacconelli E, Thwaites GE. Critical analysis of antibacterial agents in clinical development. Nat Rev Microbiol. 2020;18(5):286–298. 32152509 10.1038/s41579-020-0340-0

[3] Theuretzbacher U, Outterson K, Engel A, Karlén A. The global preclinical antibacterial pipeline. Nat Rev Microbiol. 2020;18(5):275–285. 31745331 10.1038/s41579-019-0288-0 PMC7223541

[4] May KL, Grabowicz M. The bacterial outer membrane is an evolving antibiotic barrier. Proc Natl Acad Sci U S A. 2018;115(36):8852–8854. 30139916 10.1073/pnas.1812779115 PMC6130387

[5] Global Priority List of Antibiotic-Resistant Bacteria to Guide Research, Discovery, and Development of New Antibiotics. WHO 2017.

[6] Goff DA, Kullar R, Goldstein EJC, Gilchrist M, Nathwani D, Cheng AC, Cairns KA, Escandón-Vargas K, Villegas MV, Brink A, et al. A global call from five countries to collaborate in antibiotic stewardship: united we succeed, divided we might fail. Lancet Infect Dis. 2017;17(2):e56–e63. (16)30386-3. 27866945 10.1016/S1473-3099(16)30386-3

[7] Ciumac D, Gong H, Hu X, Lu JR. Membrane targeting cationic antimicrobial peptides. J Colloid Interface Sci. 2019;537:163–185. 30439615 10.1016/j.jcis.2018.10.103

[8] Vaara M. Polymyxins and their potential next generation as therapeutic antibiotics. Front Microbiol. 2019;10:1689. 31404242 10.3389/fmicb.2019.01689 PMC6671869

[9] Mollica A, Macedonio G, Stefanucci A, Costante R, Carradori S, Cataldi V, Giulio M, Cellini L, Silvestri R, Giordano C, et al. Arginine- and lysine-rich peptides: synthesis, characterization and antimicrobial activity. Lett. Drug Des. Discov. 2017;14:1–7.

[10] Ledger EVK, Sabnis A, Edwards AM. Polymyxin and lipopeptide antibiotics: membrane-targeting drugs of last resort. Microbiology (Reading). 2022;168 (2):001136. 35118938 10.1099/mic.0.001136 PMC8941995

[11] Grein F, Müller A, Scherer KM, Liu X, Ludwig KC, Klöckner A, Strach M, Sahl H-G, Kubitscheck U, Schneider T. Ca2+-daptomycin targets cell wall biosynthesis by forming a tripartite complex with undecaprenyl-coupled intermediates and membrane lipids. Nat Commun. 2020;11(1):1455. 32193379 10.1038/s41467-020-15257-1 PMC7081307

[12] Falagas ME, Kasiakou SK, Saravolatz LD. Colistin: the revival of polymyxins for the management of multidrug-resistant Gram-negative bacterial infections. Clin Infect Dis. 2005;40(9):1333–1341. 15825037 10.1086/429323

[13] Müller A, Wenzel M, Strahl H, Grein F, Saaki TNV, Kohl B, Siersma T, Bandow JE, Sahl H-G, Schneider T, et al. Daptomycin inhibits cell envelope synthesis by interfering with fluid membrane microdomains. Proc Natl Acad Sci U S A. 2016;113(45):E7077–E7086. 27791134 10.1073/pnas.1611173113 PMC5111643

[14] Lin S, Wade JD, Liu S. De novo design of flavonoid-based mimetics of cationic antimicrobial peptides: discovery, development, and applications. Acc Chem Res. 2021;54(1):104–119. 33346639 10.1021/acs.accounts.0c00550

[15] Spaeth A, Graeler A, Maisch T, Plaetzer K. CureCuma-cationic curcuminoids with improved properties and enhanced antimicrobial photodynamic activity. Eur J Med Chem. 2018;159:423–440. 29331487 10.1016/j.ejmech.2017.09.072

[16] Xu G-L, Liu F, Zhao Y, Ao G-Z, Xi L, Ju M, Xue T. Biological evaluation of 2-(4-amino-phenyl)-3-(3,5-dihydroxylphenyl) propenoic acid. Basic Clin Pharmacol Toxicol. 2009; 105 (5):350–356. 19744157 10.1111/j.1742-7843.2009.00463.x

[17] Fujita Y, Islam R, Sakai K, Kaneda H, Kudo K, Tamura D, Aomatsu K, Nagai T, Kimura H, Matsumoto K, et al. Aza-derivatives of resveratrol are potent macrophage migration inhibitory factor inhibitors. Invest New Drugs. 2012;30(5):1878–1886. 21912888 10.1007/s10637-011-9749-7

[18] Hernández-Valdepeña MA, Hernández-Valencia CG, Labra-Vázquez P, Wacher C, Díaz-Ruiz G, Vázquez A, Pedraza-Chaverri J, Shirai K, Rosas-Aburto A, Vivaldo-Lima E, et al. Antioxidant and antimicrobial material by grafting of l-arginine onto enzymatic poly(gallic acid). Mater Sci Eng C Mater Biol Appl. 2021;121:111650. 33579431 10.1016/j.msec.2020.111650

[19] Biscussi B, Richmond V, Baier CJ, Mañez PA, Murray AP. Design and microwave-assisted synthesis of aza-resveratrol analogs with potent cholinesterase inhibition. CNS Neurol Disord Drug Targets. 2020;19(8):630–641. 32888280 10.2174/1871527319666200905121536

[20] Roberti M, Pizzirani D, Simoni D, Rondanin R, Baruchello R, Bonora C, Buscemi F, Grimaudo S, Tolomeo M. Synthesis and biological evaluation of resveratrol and analogues as apoptosis-inducing agents. J Med Chem. 2003;46(16):3546–3554. 12877593 10.1021/jm030785u

[21] Chillemi R, Sciuto S, Spatafora C, Tringali C. Anti-tumor properties of stilbene-based resveratrol analogues: recent results. Nat Prod Commun . 2007;2(4):1934578X0700200.

[22] Paul S, Mizuno CS, Lee HJ, Zheng X, Chajkowisk S, Rimoldi JM, Conney A, Suh N, Rimando AM. In vitro and in vivo studies on stilbene analogs as potential treatment agents for colon cancer. Eur J Med Chem. 2010;45(9):3702–3708. 20627379 10.1016/j.ejmech.2010.05.019 PMC2918699

[23] Siddiqui A, Dandawate P, Rub R, Padhye S, Aphale S, Moghe A, Jagyasi A, Venkateswara Swamy K, Singh B, Chatterjee A, et al. Novel aza-resveratrol analogs: synthesis, characterization and anticancer activity against breast cancer cell lines. Bioorg Med Chem Lett. 2013;23(3):635–640. 23273518 10.1016/j.bmcl.2012.12.002

[24] Wang S, Willenberg I, Krohn M, Hecker T, Meckelmann S, Li C, Pan Y, Schebb NH, Steinberg P, Empl MT. Growth-inhibiting activity of resveratrol imine analogs on tumor cells in vitro. Plos One. 2017;12(1):e0170502. 28114318 10.1371/journal.pone.0170502 PMC5256997

[25] Lizard G, Latruffe N, Vervandier-Fasseur D. Aza- and azo-stilbenes: bio-isosteric analogs of resveratrol. Molecules. 2020;25(3):605. 32019195 10.3390/molecules25030605 PMC7037676

[26] Tolomeo M, Roberti M, Scapozza L, Tarantelli C, Giacomini E, Titone L, Saporito L, Di Carlo P, Colomba C. TTAS a new stilbene derivative that induces apoptosis in leishmania infantum. Exp Parasitol. 2013;133(1):37–43. 23103597 10.1016/j.exppara.2012.10.006

[27] Belmonte-Reche E, Peñalver P, Caro-Moreno M, Mateos-Martín ML, Adán N, Delgado M, González-Rey E, Morales JC. Silyl resveratrol derivatives as potential therapeutic agents for neurodegenerative and neurological diseases. Eur J Med Chem. 2021;223:113655. 34175536 10.1016/j.ejmech.2021.113655

[28] Mattarei A, Biasutto L, Romio M, Zoratti M, Paradisi C. Synthesis of resveratrol sulfates: turning a nightmare into a dream. Tetrahedron. 2015; 71 (20):3100–3106.

[29] Zhang Z, Yu B, Schmidt RR. Synthesis of mono- and di-o-β-d-glucopyra©ide conjugates of (e)-resveratrol. Synthesis. 2006;2006(8):1301–1306.

[30] Ensch C, Hesse M. Total syntheses of the spermine alkaloids (−)-(r,r)-hopromine and (±)-homaline. HCA. 2002;85 (6):1659–1677.

[31] Peñalver P, Belmonte-Reche E, Adán N, Caro M, Mateos-Martín ML, Delgado M, González-Rey E, Morales JC. Alkylated resveratrol prodrugs and metabolites as potential therapeutics for neurodegenerative diseases. Eur J Med Chem. 2018;146:123–138. 29407944 10.1016/j.ejmech.2018.01.037

[32] Mungall WS, Greene GL, Heavner GA, Letsinger RL. Use of azido group in the synthesis of 5’ terminal aminodeoxythymidine oligonucleotides. J Org Chem. 1975; 40 (11):1569–1562. 1142038 10.1021/jo00899a038

[33] Butler MS, Paterson DL. Antibiotics in the clinical pipeline in October 2019. J Antibiot (Tokyo)). 2020;73(6):329–364. 32152527 10.1038/s41429-020-0291-8 PMC7223789

[34] Falagas ME, Rafailidis PI, Matthaiou DK. Resistance to polymyxins: mechanisms, frequency and treatment options. Drug Resist Updat. 2010;13(4-5):132–138. 20843473 10.1016/j.drup.2010.05.002

[35] Tan J, Zhao Y, Hedrick JL, Yang YY. Effects of hydrophobicity on antimicrobial activity, selectivity, and functional mechanism of guanidinium-functionalized polymers. Adv Healthcare Materials. 2022;11(7):2100482. 10.1002/adhm.202100482 33987953

[36] Phuong PT, Oliver S, He J, Wong EHH, Mathers RT, Boyer C. Effect of hydrophobic groups on antimicrobial and hemolytic activity: developing a predictive tool for ternary antimicrobial polymers. Biomacromolecules. 2020;21(12):5241–5255. 33186496 10.1021/acs.biomac.0c01320

[37] *Molinspiration Cheminformatics*. 2022. [Accessed 2022 Jun 21]. https://www.molinspiration.com/

[38] Raheem N, Straus SK. Mechanisms of action for antimicrobial peptides with antibacterial and antibiofilm functions. Front Microbiol. 2019;10:2866. 31921046 10.3389/fmicb.2019.02866 PMC6927293

[39] Gogry FA, Siddiqui MT, Sultan I, Haq Q, Mohd R. Current update on intrinsic and acquired colistin resistance mechanisms in bacteria. Front Med. 2021;8:677720. 10.3389/fmed.2021.677720 PMC8406936 34476235

[40] Li Q, Cebrián R, Montalbán-López M, Ren H, Wu W, Kuipers OP. Outer-membrane-acting peptides and lipid ii-targeting antibiotics cooperatively kill Gram-negative pathogens. Commun Biol. 2021;4(1):31. 33398076 10.1038/s42003-020-01511-1 PMC7782785

[41] Xia Y, Cebrián R, Xu C, Jong A. d, Wu W, Kuipers OP. Elucidating the mechanism by which synthetic helper peptides sensitize pseudomonas aeruginosa to multiple antibiotics. PLoS Pathog. 2021;17(9):e1009909. 34478485 10.1371/journal.ppat.1009909 PMC8445441

[42] Vaara M. Agents that increase the permeability of the outer membrane. Microbiol Rev. 1992;56(3):395–411. 1406489 10.1128/mr.56.3.395-411.1992 PMC372877

[43] Liu Y, Ding S, Shen J, Zhu K. Nonribosomal antibacterial peptides that target multidrug-resistant bacteria. Nat Prod Rep. 2019;36(4):573–592. 30324212 10.1039/c8np00031j

[44] Song M, Liu Y, Huang X, Ding S, Wang Y, Shen J, Zhu K. A broad-spectrum antibiotic adjuvant reverses multidrug-resistant gram-negative pathogens. Nat Microbiol. 2020;5(8):1040–1050. 32424338 10.1038/s41564-020-0723-z

[45] Foged C, Nielsen HM. Cell-penetrating peptides for drug delivery across membrane barriers. Expert Opin Drug Deliv. 2008;5(1):105–117. 18095931 10.1517/17425247.5.1.105

[46] Bouarab-Chibane L, Forquet V, Lantéri P, Clément Y, Léonard-Akkari L, Oulahal N, Degraeve P, Bordes C. Antibacterial properties of polyphenols: characterization and qsar (quantitative structure–activity relationship) models. Front Microbiol. 2019;10:829. 31057527 10.3389/fmicb.2019.00829 PMC6482321

[47] Te Winkel JD, Gray DA, Seistrup KH, Hamoen LW, Strahl H. Analysis of antimicrobial-triggered membrane depolarization using voltage sensitive dyes. Front Cell Dev Biol. 2016;4:29. 27148531 10.3389/fcell.2016.00029 PMC4829611

[48] Yamamura H, Hagiwara T, Hayashi Y, Osawa K, Kato H, Katsu T, Masuda K, Sumino A, Yamashita H, Jinno R, et al. Antibacterial activity of membrane-permeabilizing bactericidal cyclodextrin derivatives. ACS Omega. 2021;6(47):31831–31842. 34870006 10.1021/acsomega.1c04541 PMC8638021

[49] Vaara M. New polymyxin derivatives that display improved efficacy in animal infection models as compared to polymyxin B and colistin. Med Res Rev. 2018;38(5):1661–1673. 29485690 10.1002/med.21494

[50] Kanafani ZA, Corey GR. Daptomycin: a rapidly bactericidal lipopeptide for the treatment of gram-positive infections. Expert Rev anti Infect Ther. 2007;5(2):177–184. 17402833 10.1586/14787210.5.2.177

[51] Egan AJF. Bacterial outer membrane constriction. Mol Microbiol. 2018;107(6):676–687. 29315884 10.1111/mmi.13908

[52] Wexler HM. Outer-membrane pore-forming proteins in Gram-negative anaerobic bacteria. Clin Infect Dis. 2002;35(Suppl 1):S65–S71. 12173111 10.1086/341923

[53] Cebrián R, Xu C, Xia Y, Wu W, Kuipers OP. The cathelicidin-derived close-to-nature peptide d-11 sensitises klebsiella pneumoniae to a range of antibiotics in vitro, ex vivo and in vivo. Int J Antimicrob Agents. 2021;58(5):106434. 34525402 10.1016/j.ijantimicag.2021.106434

[54] Corbett D, Wise A, Langley T, Skinner K, Trimby E, Birchall S, Dorali A, Sandiford S, Williams J, Warn P, et al. Potentiation of antibiotic activity by a novel cationic peptide: potency and spectrum of activity of SPR741. Antimicrob Agents Chemother. 2017;61(8):e00200–17. 28533232 10.1128/AAC.00200-17 PMC5527571

[55] Zurawski DV, Reinhart AA, Alamneh YA, Pucci MJ, Si Y, Abu-Taleb R, Shearer JP, Demons ST, Tyner SD, Lister T. SPR741, an antibiotic adjuvant, potentiates the in vitro and in vivo activity of rifampin against clinically relevant extensively drug-resistant acinetobacter baumannii. Antimicrob Agents Chemother. 2017;61(12):e01239–17. 28947471 10.1128/AAC.01239-17 PMC5700309

[56] She P, Liu Y, Xu L, Li Y, Li Z, Liu S, Hussain Z, Wu Y. SPR741, double- or triple-combined with erythromycin and clarithromycin, combats drug-resistant klebsiella pneumoniae, its biofilms, and persister cells. Front Cell Infect Microbiol. 2022;12:858606. 35372124 10.3389/fcimb.2022.858606 PMC8971605

[57] Lenhard JR, Nation RL, Tsuji BT. Synergistic combinations of polymyxins. Int J Antimicrob Agents. 2016;48(6):607–613. 27865626 10.1016/j.ijantimicag.2016.09.014 PMC5237374

[58] MacNair CR, Brown ED. Outer membrane disruption overcomes intrinsic, acquired, and spontaneous antibiotic resistance. mBio. 2020;11 (5):e01615–20. 32963002 10.1128/mBio.01615-20 PMC7512548

[59] Stokes JM, MacNair CR, Ilyas B, French S, Côté J-P, Bouwman C, Farha MA, Sieron AO, Whitfield C, Coombes BK, et al. Pentamidine sensitizes Gram-negative pathogens to antibiotics and overcomes acquired colistin resistance. Nat Microbiol. 2017;2:17028. 28263303 10.1038/nmicrobiol.2017.28 PMC5360458

[60] Kuroda K, Caputo GA, DeGrado WF. The role of hydrophobicity in the antimicrobial and hemolytic activities of polymethacrylate derivatives. Chemistry. 2009;15(5):1123–1133. 19072946 10.1002/chem.200801523 PMC3814040

[61] Hollmann A, Martínez M, Noguera ME, Augusto MT, Disalvo A, Santos NC, Semorile L, Maffía PC. Role of amphipathicity and hydrophobicity in the balance between hemolysis and peptide–membrane interactions of three related antimicrobial peptides. Colloids Surf B Biointerfaces. 2016;141:528–536. 26896660 10.1016/j.colsurfb.2016.02.003

[62] Jiang Y, Chen Y, Song Z, Tan Z, Cheng J. Recent advances in design of antimicrobial peptides and polypeptides toward clinical translation. Adv Drug Deliv Rev. 2021;170:261–280. 33400958 10.1016/j.addr.2020.12.016

[63] Sahm DF, Kissinger J, Gilmore MS, Murray PR, Mulder R, Solliday J, Clarke B. In vitro susceptibility studies of vancomycin-resistant enterococcus faecalis. Antimicrob Agents Chemother. 1989;33(9):1588–1591. 2554802 10.1128/aac.33.9.1588 PMC172707

[64] Clinical and Laboratory Standards Institute. Methods for dilution antimicrobial susceptibility tests for bacteria that grow aerobically: M07-A10 . Approved Standard, 10. ed. Wayne(PA): Documents/Clinical and Laboratory Standards Institute; Committee for Clinical Laboratory Standards; 2015.

[65] Cebrián R, Belmonte-Reche E, Pirota V, de Jong A, Morales JC, Freccero M, Doria F, Kuipers OP. G-Quadruplex DNA as a target in pathogenic bacteria: efficacy of an extended naphthalene diimide ligand and its mode of action. J Med Chem. 2022;65(6):4752–4766. 34928608 10.1021/acs.jmedchem.1c01905 PMC8958502

[66] Cebrián R, Rodríguez-Cabezas ME, Martín-Escolano R, Rubiño S, Garrido-Barros M, Montalbán-López M, Rosales MJ, Sánchez-Moreno M, Valdivia E, Martínez-Bueno M, et al. Preclinical studies of toxicity and safety of the AS-48 bacteriocin. J Adv Res. 2019;20:129–139. 31360546 10.1016/j.jare.2019.06.003 PMC6637140

[67] Mosmann T. Rapid colorimetric assay for cellular growth and survival: application to proliferation and cytotoxicity assays. J Immunol Methods. 1983;65(1-2):55–63. 6606682 10.1016/0022-1759(83)90303-4

