# Supplementary material for: Synthesis and antimicrobial activity of aminoalkyl resveratrol derivatives inspired by cationic peptides
Source: J Enzyme Inhib Med Chem. 2023 Jan 4;38(1):267–81. doi: 10.1080/14756366.2022.2146685 (PMC9828810; doi:10.1080/14756366.2022.2146685)
Supplement: Supplemental Material [file IENZ_A_2146685_SM2136.pdf]

Synthesis and antimicrobial activity of aminoalkyl resveratrol derivatives inspired by cationic peptides.

Rubén Cebrián,[a,b]* Ricardo Lucas,[c] María Victoria Fernández-Cantos,[a] Koen Slot,[a] Pablo Peñalver, [d] Marta Martínez-García,[a] Antonio Párraga-Leo,[c] María Violante de Paz,[c] Federico García,[b] Oscar P. Kuipers[a,]* and Juan Carlos Morales[d,]*

Affiliations

[a] Department of Molecular Genetics, Groningen Biomolecular Sciences and Biotechnology Institute, University of Groningen, Nijenborgh 7, 9747AG, Groningen, The Netherlands.

[b]Department of Clinical Microbiology, Instituto de Investigación Biosanitaria ibs. GRANADA, University Hospital Clínico San Cecilio, Av. de la Innovación s/n, 18061, Granada, Spain.

[c] Department of Organic and Pharmaceutical Chemistry, School of Pharmacy, University of Seville, 41012, Seville, Spain.

[d] Department of Biochemistry and Molecular Pharmacology and Department of Cellular Biology and Immunology. Instituto de Parasitología y Biomedicina López Neyra, CSIC, PTS Granada, Avda. del Conocimiento, 17, 18016 Armilla, Granada, Spain.

Corresponding Authors

* R.C.: phone, +34-958021781 ; e-mail: rcebrian@ugr.es

* O.P.K.: phone, +31-503632093; email: o.p.kuipers@rug.nl

* J.C.M.: phone, +34-958181644; e-mail: jcmorales@ipb.csic.es

Table of Contents.

Supplementary Figure 1. *E. coli* LMG 8224 outer membrane permeabilization by the different amino RES designed. Page S2

Supplementary Figure 2. *E. coli* LMG 8224 inner membrane permeabilization by the different amino RES designed. Page S3

Supplementary Figure 3. Haemolytic activity of the different amino RES derivatives Page S4

Supplementary Table 1. Selective index HC_{50}/MIC Page S5

^{1}H and ^{13}C-NMR spectra of intermediates 10, 13-20 Pages S6-S14

^{1}H and ^{13}C-NMR spectra of final compounds 2-8 Pages S15-S21

High resolution mass spectra of final compounds 2-8 Pages S22-S25

HPLC chromatograms of final compounds 2-8 Pages S26-S28

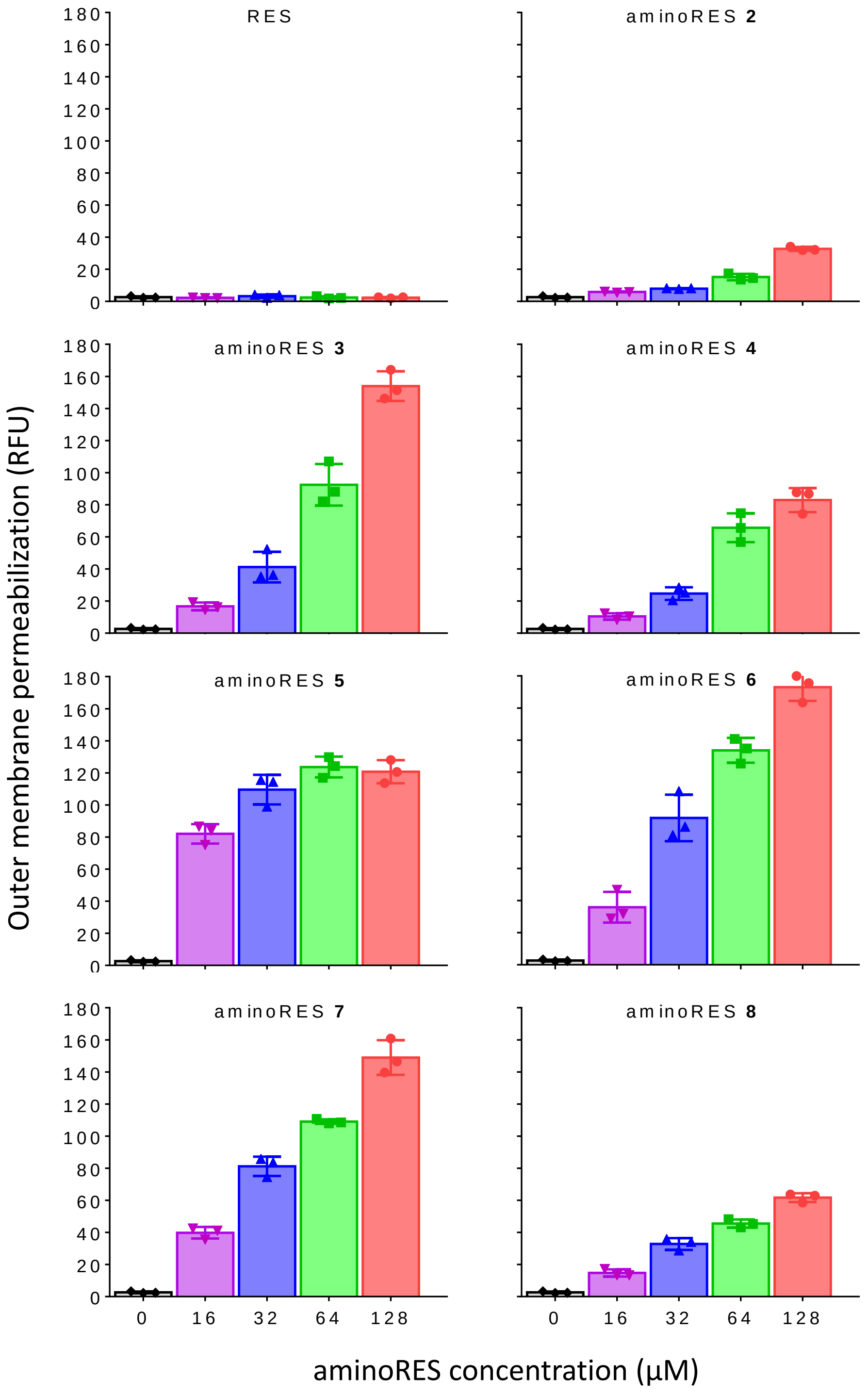

Supplementary figure 1: *E. coli* LMG 8224 outer membrane permeabilization by the different amino RES designed.

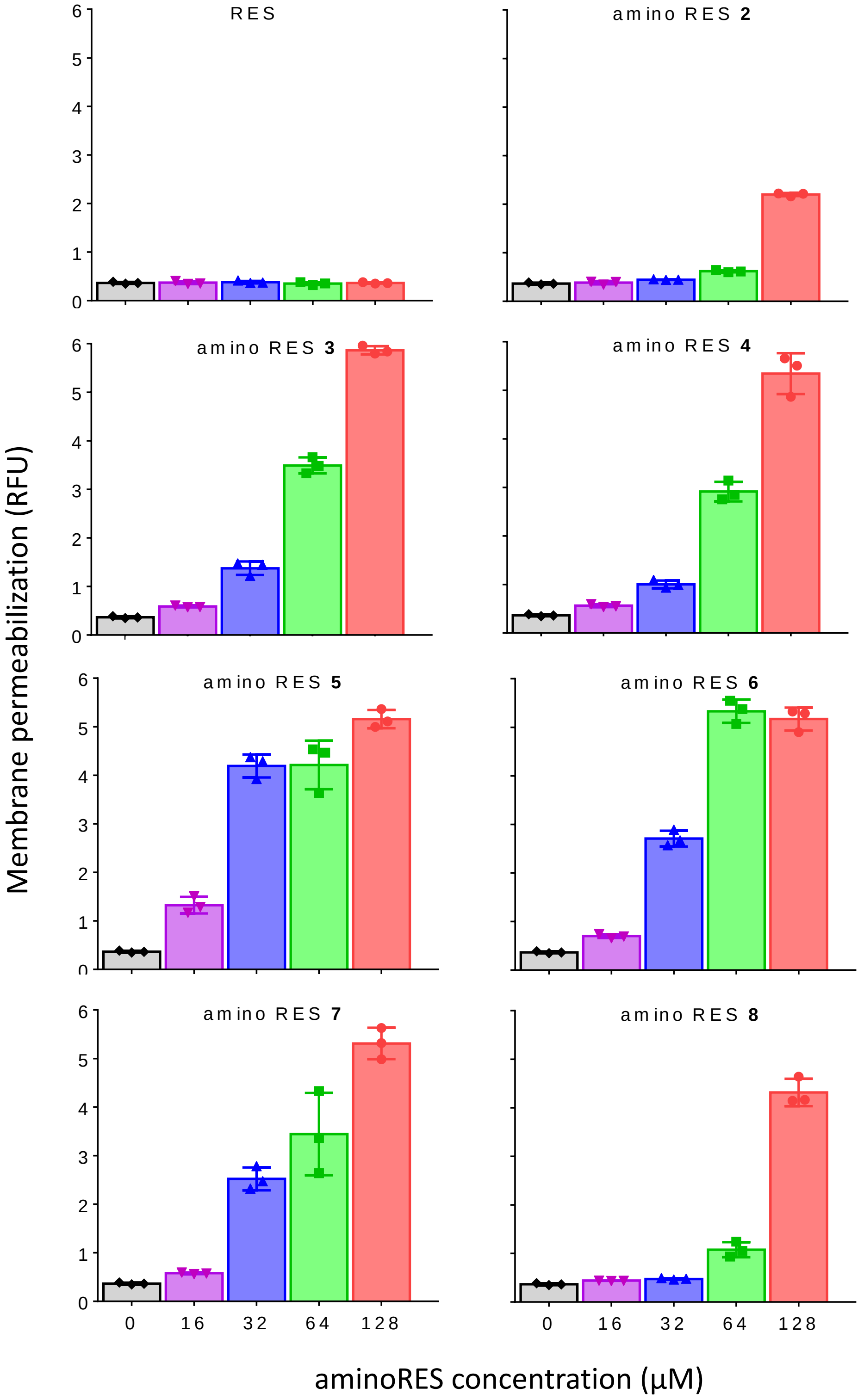

Supplementary figure 2: *E. coli* LMG 8224 inner membrane permeabilization by the different amino RES designed.

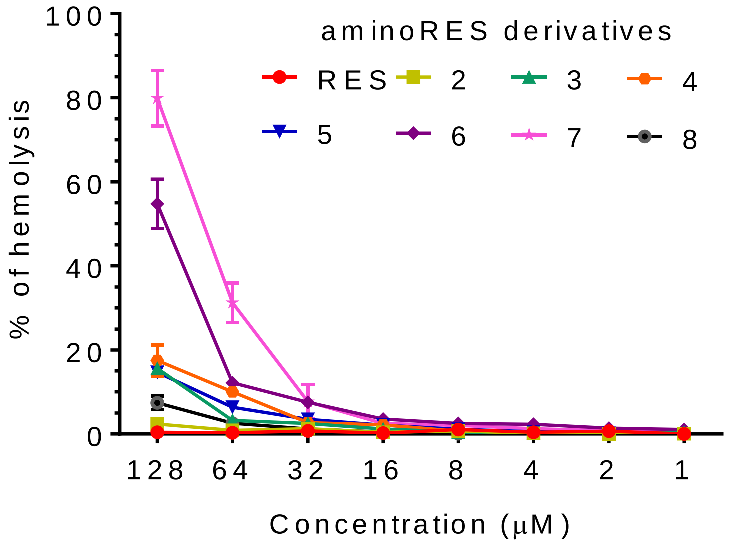

Supplementary Figure 3: Haemolytic activity of the different amino RES derivatives.

	RES	2	3	4	5	6	7	8
HC_{50}	>128	>128	>128	>128	>128	124.43	79.90	>128
A. baumannii LMG 01041	-	-	2	2	8	-	-	-
K. aerogenes LMG 02094	-	-	-	-	4	-	-	-
E. cloacae LMG 02783	-	-	2	2	8	-	-	-
E. coli LMG 8224	-	-	2	2	16	0.97	-	2
E. coli NCTC 13846	-	-	-	-	19.20	-	-	2
K. pneumoniae LMG 20218	-	-	-	-	4	-	-	-
P. aeruginosa PAO1	-	-	-	-	4	-	-	-
S. enterica LMG 07233	-	-	-	-	8	-	-	2
B. cereus LMG 10987	-	-	2	3	9.60	1.46	0.62	-
B. cereus LMG 14574	-	-	2.4	4	8	3.89	0.62	2
E. faecalis V583	-	-	-	-	9.60	-	-	-
E. faecalis LMG 8222	-	-	4.80	6	16	7.78	-	2
E. faecalis LMG 16716	-	-	4	4	19.20	7.78	-	-
E. faecium LMG 11423	-	-	4	8	16	15.55	-	2
E. faecium LMG 16003	-	-	2	2	16	-	-	2
S. aureus LMG 15975	-	-	4.80	8	76.80	7.78	0.62	2
S. aureus LMG 8223	-	2	6	6	32	15.55	1.25	2
S. aureus LMG 10147	-	-	-	-	19.20	0.97	-	2
Bacteroides ovatus 3_8_47FAA	-	-	12.02	8	16	15.55	2.50	-
B. fragilis NCTC 9343	-	-	8	8	16	6.69	1.25	-
B. salyersiae DMS 18765	-	-	8	8	16	9.36	1.50	-
B. xylanisolvens DMS 18836	-	2	16	16	32	15.55	2.50	2
Parabacteroides merdae CL03T12C32	-	-	-	-	4	-	-	-
C. botulinum CECT 551	2	2	32	16	128	124.43	9.99	2
C. difficile CECT 531	-	2	16	16	64	31.11	4.99	-
C. perfringens CECT 376	-	2	16	16	12	62.22	-	-
C. tetani CECT 462	-	-	19.20	16	32	124.43	3.75	-

Supplementary table 1: Selective index HC_{50}/MIC. For the compounds in which HC_{50} was not detected in the assay 2X the higher tested concentration was used in the calculations.

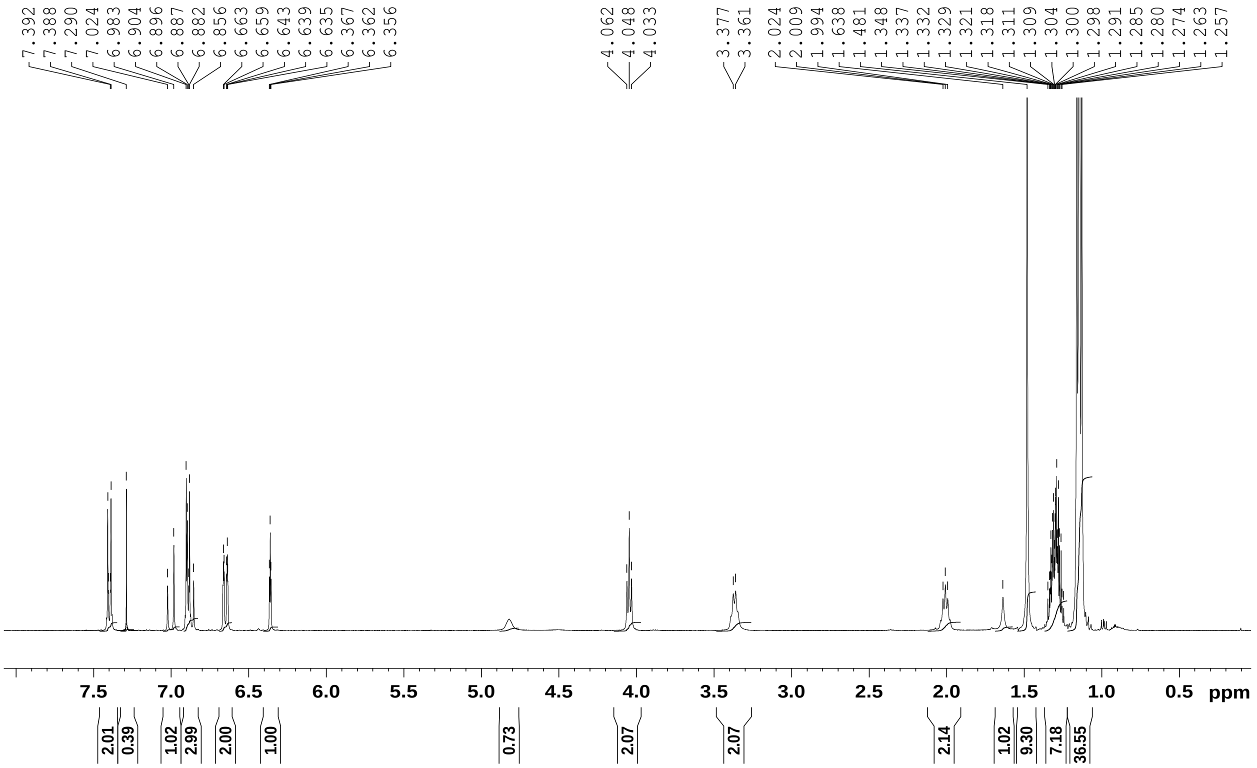
7.392
7.388
7.290
7.024
6.983
6.904
6.896
6.887
6.882
6.856
6.663
6.659
6.643
6.639
6.635
6.367
6.362
6.356
4.062
4.048
4.033
3.377
3.361
2.024
2.009
1.994
1.638
1.481
1.348
1.337
1.332
1.329
1.321
1.318
1.311
1.309
1.304
1.300
1.298
1.291
1.285
1.280
1.274
1.263
1.257
7.5 7.0 6.5 6.0 5.5 5.0 4.5 4.0 3.5 3.0 2.5 2.0 1.5 1.0 0.5 ppm
2.01
0.39
1.02
2.99
2.00
1.00
0.73
2.07
2.07
2.14
1.02
9.30
7.18
36.55

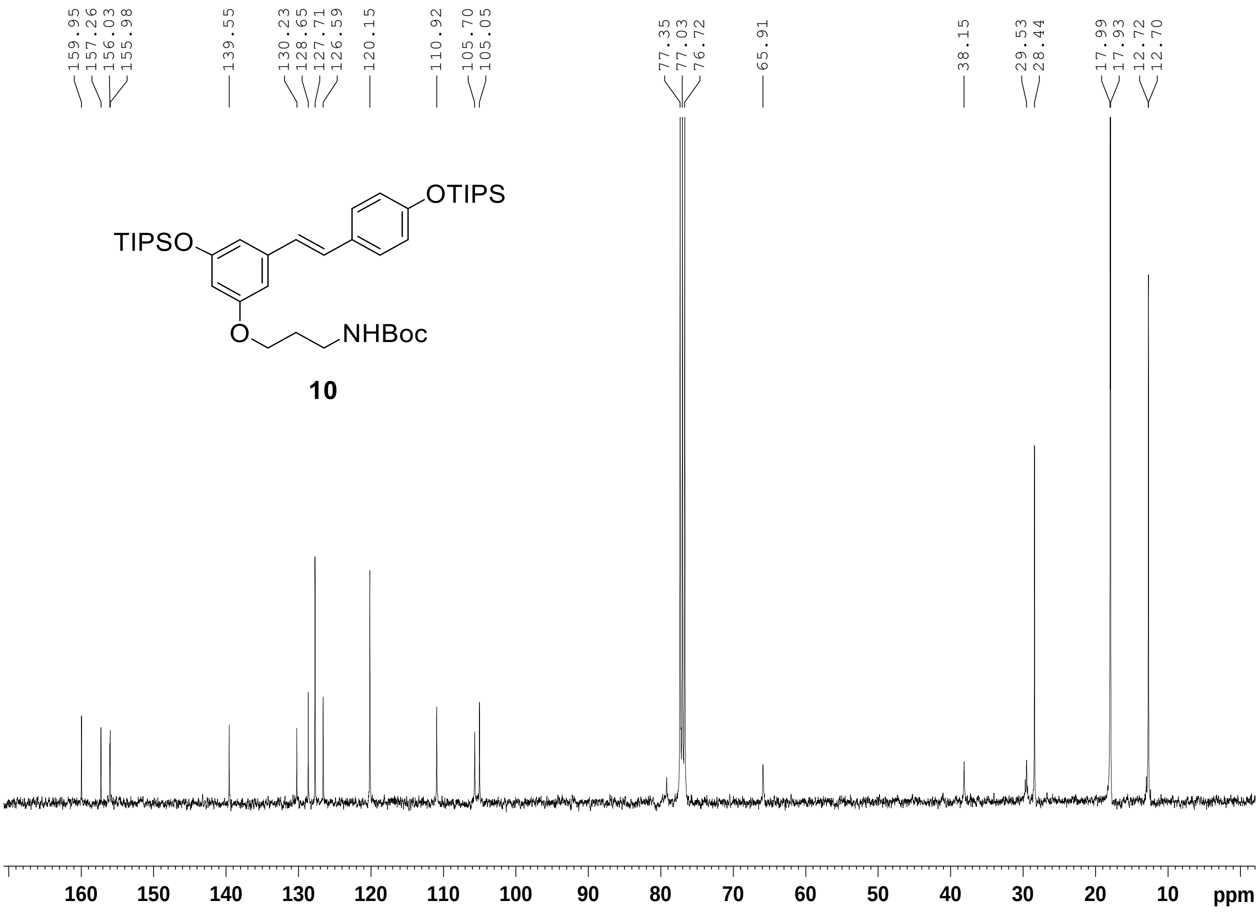
TIPSO
OTIPS
O
NHBoc
10
159.95
157.26
156.03
155.98
139.55
130.23
128.65
127.71
126.59
120.15
110.92
105.70
105.05
77.35
77.03
76.72
65.91
38.15
29.53
28.44
17.99
17.93
12.72
12.70
160 150 140 130 120 110 100 90 80 70 60 50 40 30 20 10 ppm

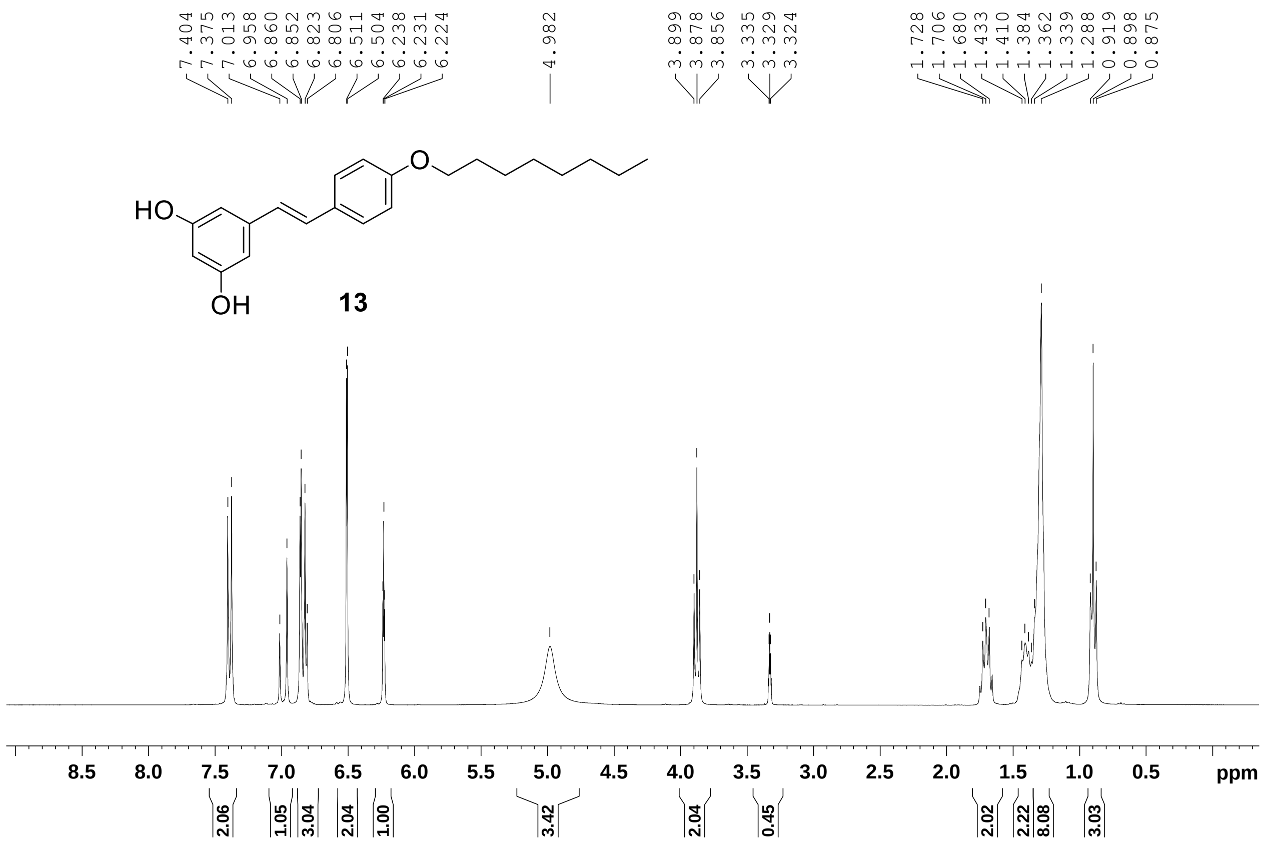
7.404 7.375 7.013 6.958 6.860 6.852 6.823 6.806 6.511 6.504 6.238 6.231 6.224
4.982
3.899 3.878 3.856 3.335 3.329 3.324
1.728 1.706 1.680 1.433 1.410 1.384 1.362 1.339 1.288 0.919 0.898 0.875
HO
O
OH
13
8.5 8.0 7.5 7.0 6.5 6.0 5.5 5.0 4.5 4.0 3.5 3.0 2.5 2.0 1.5 1.0 0.5 ppm
2.06 1.05 3.04 2.04 1.00 3.42 2.04 0.45 2.02 2.22 8.08 3.03

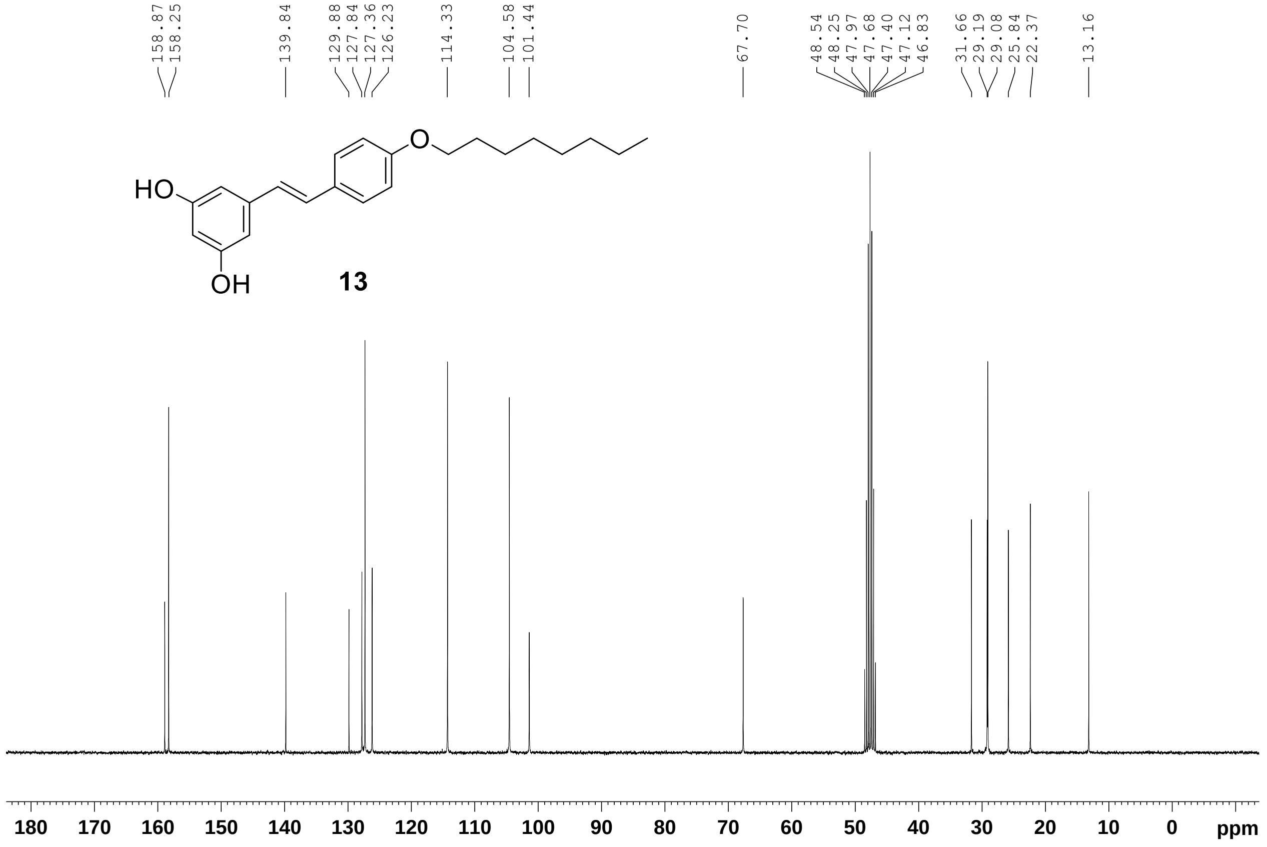
158.87 158.25 139.84 129.88 127.84 127.36 126.23 114.33 104.58 101.44 67.70 48.54 48.25 47.97 47.68 47.40 47.12 46.83 31.66 29.19 29.08 25.84 22.37 13.16
HO
O
OH
13
180 170 160 150 140 130 120 110 100 90 80 70 60 50 40 30 20 10 0 ppm

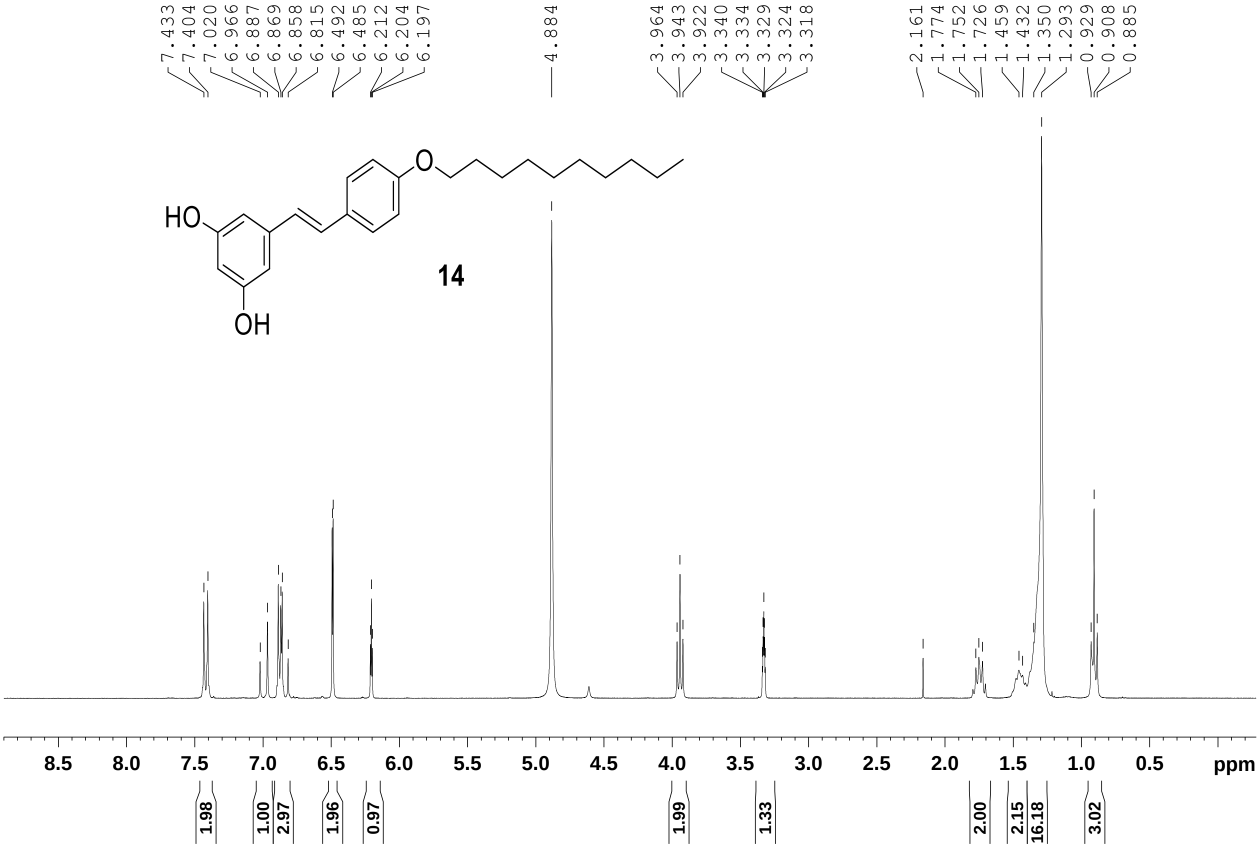
7.433
7.404
7.020
6.966
6.887
6.869
6.858
6.815
6.492
6.485
6.212
6.204
6.197
4.884
3.964
3.943
3.922
3.340
3.334
3.329
3.324
3.318
2.161
1.774
1.752
1.726
1.459
1.432
1.350
1.293
0.929
0.908
0.885
HO
OH
O
14
8.5
8.0
7.5
7.0
6.5
6.0
5.5
5.0
4.5
4.0
3.5
3.0
2.5
2.0
1.5
1.0
0.5
ppm
1.98
1.00
2.97
1.96
0.97
1.99
1.33
2.00
2.15
16.18
3.02

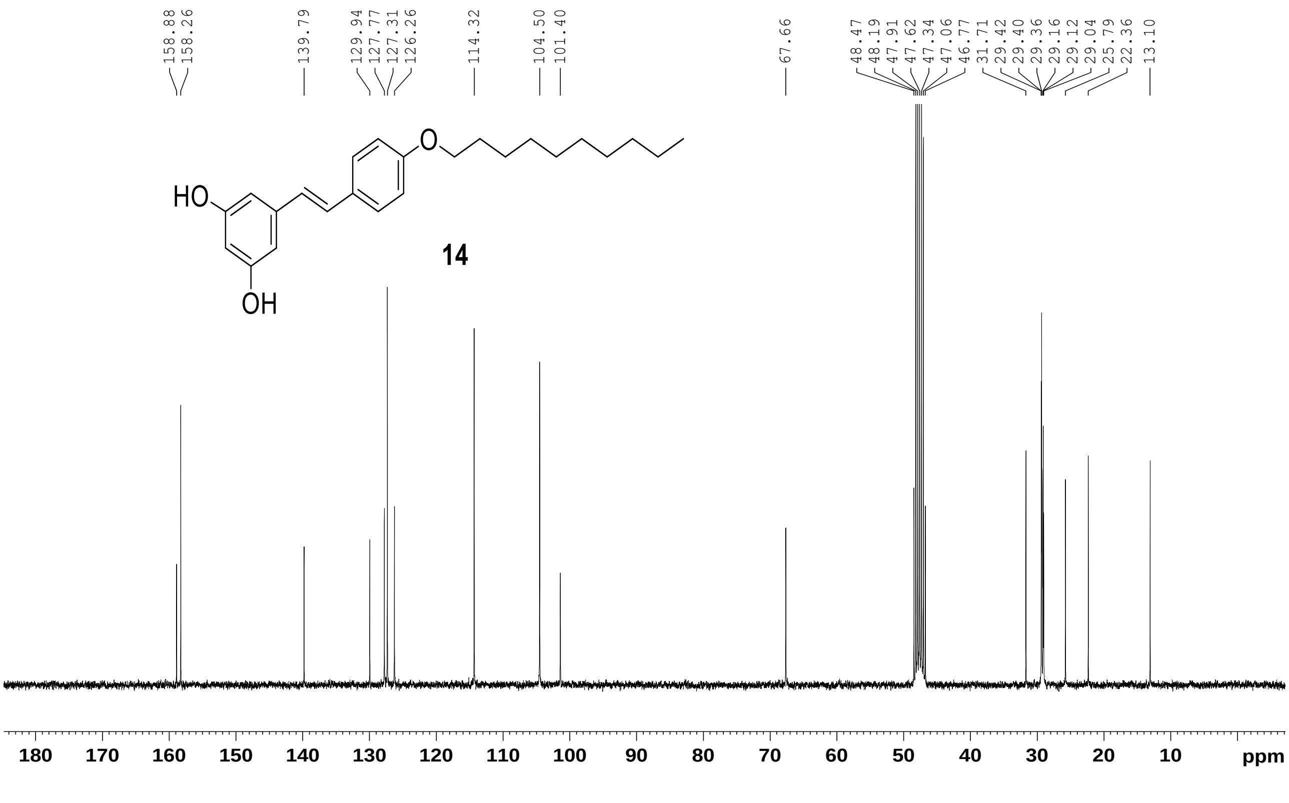
158.88
158.26
139.79
129.94
127.77
127.31
126.26
114.32
104.50
101.40
67.66
48.47
48.19
47.91
47.62
47.34
47.06
46.77
31.71
29.42
29.40
29.36
29.16
29.12
29.04
25.79
22.36
13.10
HO
OH
O
14
180
170
160
150
140
130
120
110
100
90
80
70
60
50
40
30
20
10
ppm

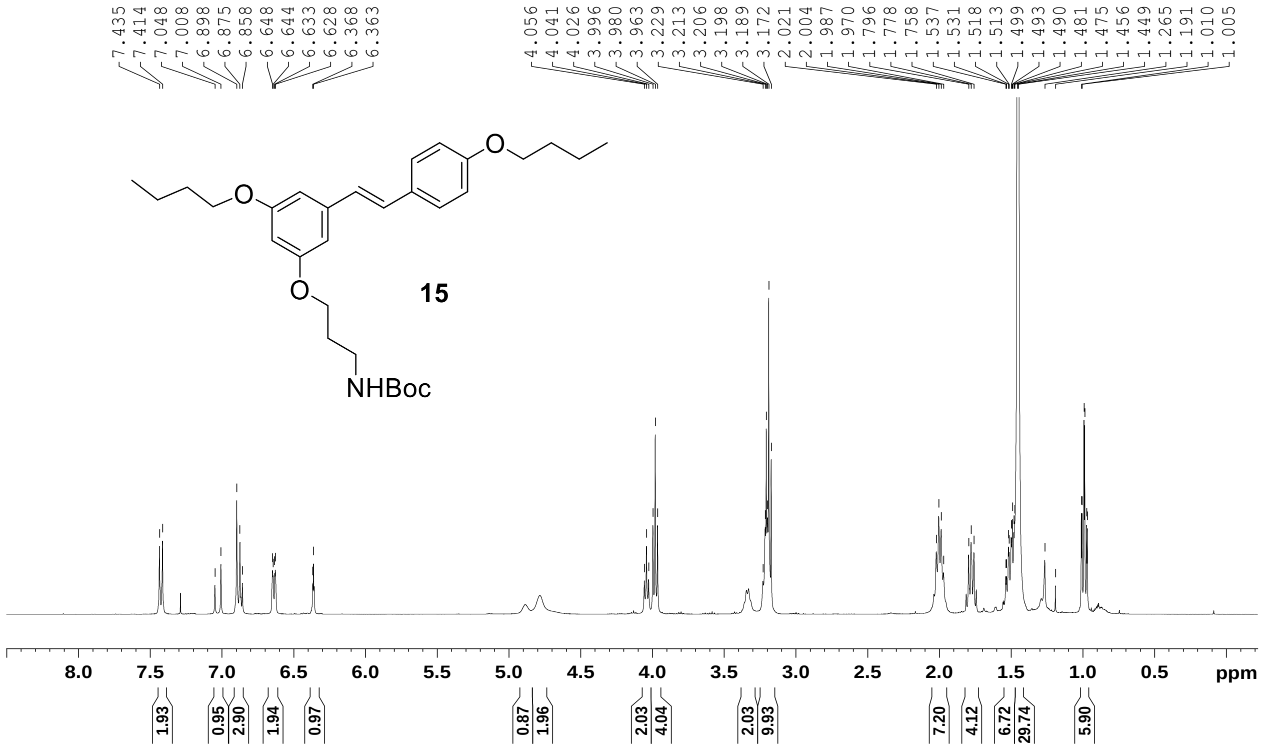

7.435
7.414
7.048
7.008
6.898
6.875
6.858
6.648
6.644
6.633
6.628
6.368
6.363
4.056
4.041
4.026
3.996
3.980
3.963
3.229
3.213
3.206
3.198
3.189
3.172
2.021
2.004
1.987
1.970
1.796
1.778
1.758
1.537
1.531
1.518
1.513
1.499
1.493
1.490
1.481
1.475
1.456
1.449
1.265
1.191
1.010
1.005
O
O
O
NHBoc
15
8.0
7.5
7.0
6.5
6.0
5.5
5.0
4.5
4.0
3.5
3.0
2.5
2.0
1.5
1.0
0.5
ppm
1.93
0.95
2.90
1.94
0.97
0.87
1.96
2.03
4.04
2.03
9.93
7.20
4.12
6.72
29.74
5.90

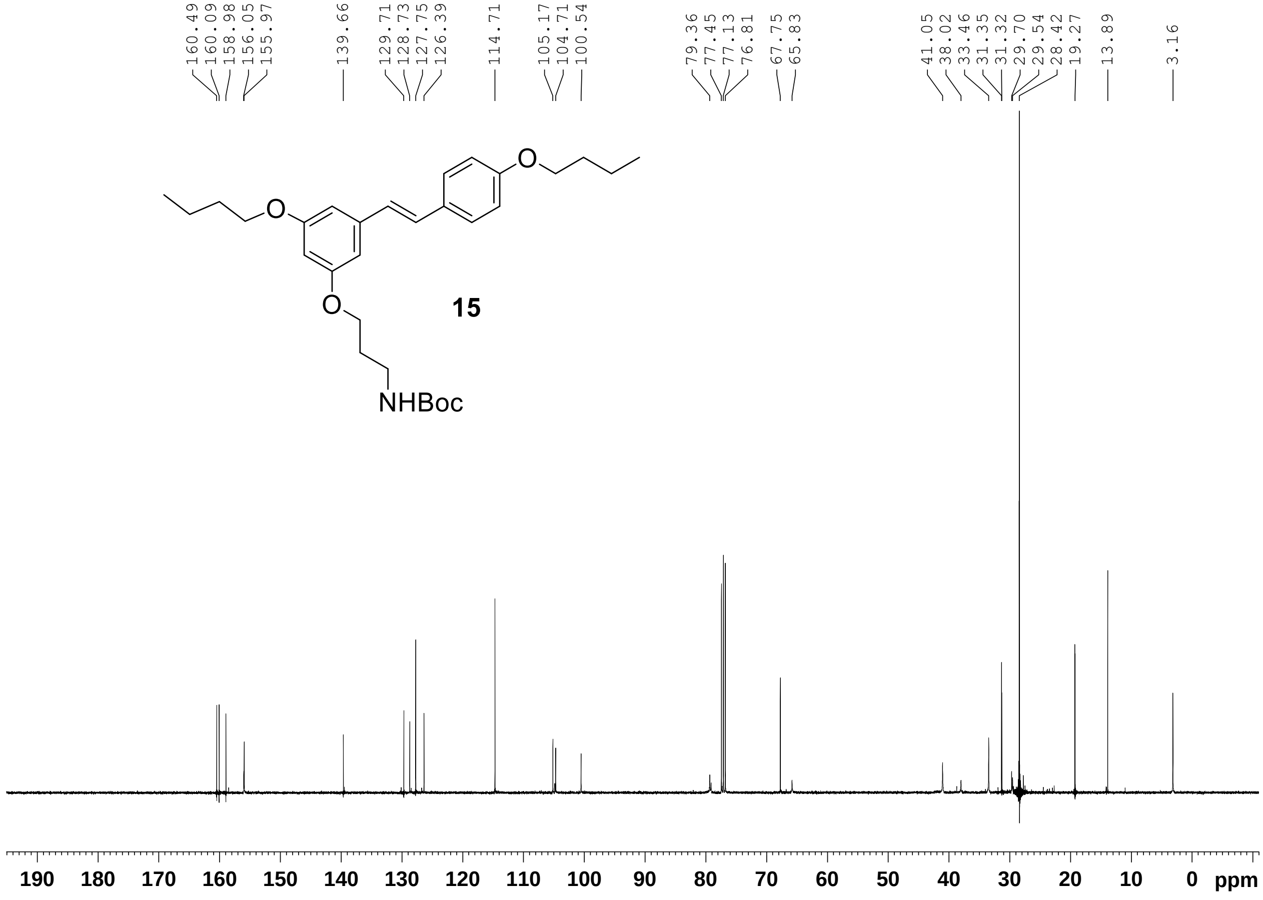

160.49
160.09
158.98
156.05
155.97
139.66
129.71
128.73
127.75
126.39
114.71
105.17
104.71
100.54
79.36
77.45
77.13
76.81
67.75
65.83
41.05
38.02
33.46
31.35
31.32
29.70
29.54
28.42
19.27
13.89
3.16
O
O
O
NHBoc
15
190
180
170
160
150
140
130
120
110
100
90
80
70
60
50
40
30
20
10
0
ppm

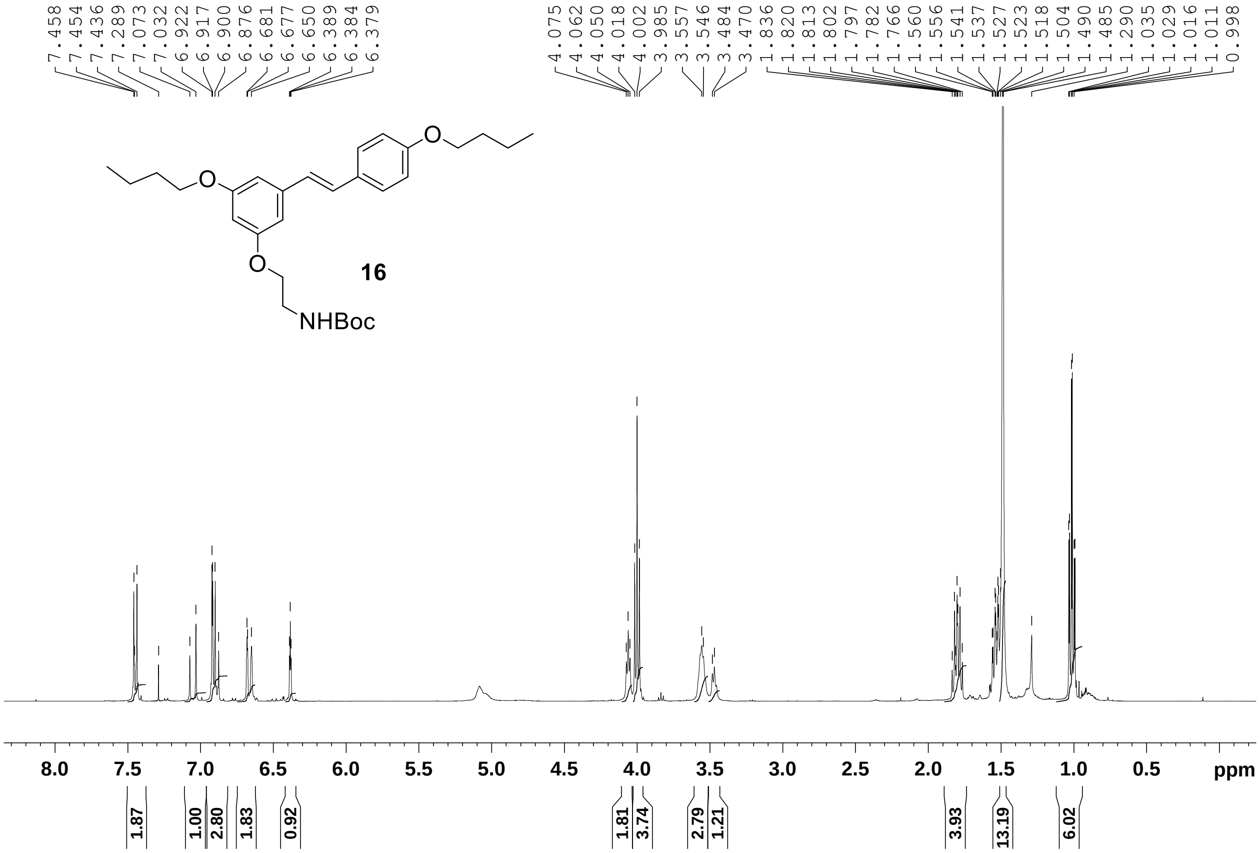
7.458 7.454 7.436 7.289 7.073 7.032 6.922 6.917 6.900 6.876 6.681 6.677 6.650 6.389 6.384 6.379
4.075 4.062 4.050 4.018 4.002 3.985 3.557 3.546 3.484 3.470 1.836 1.820 1.813 1.802 1.797 1.782 1.766 1.560 1.556 1.541 1.537 1.527 1.523 1.518 1.504 1.490 1.485 1.290 1.035 1.029 1.016 1.011 0.998
O
O
O
NHBoc
16
8.0 7.5 7.0 6.5 6.0 5.5 5.0 4.5 4.0 3.5 3.0 2.5 2.0 1.5 1.0 0.5 ppm
1.87 1.00 2.80 1.83 0.92 1.81 3.74 2.79 1.21 3.93 13.19 6.02

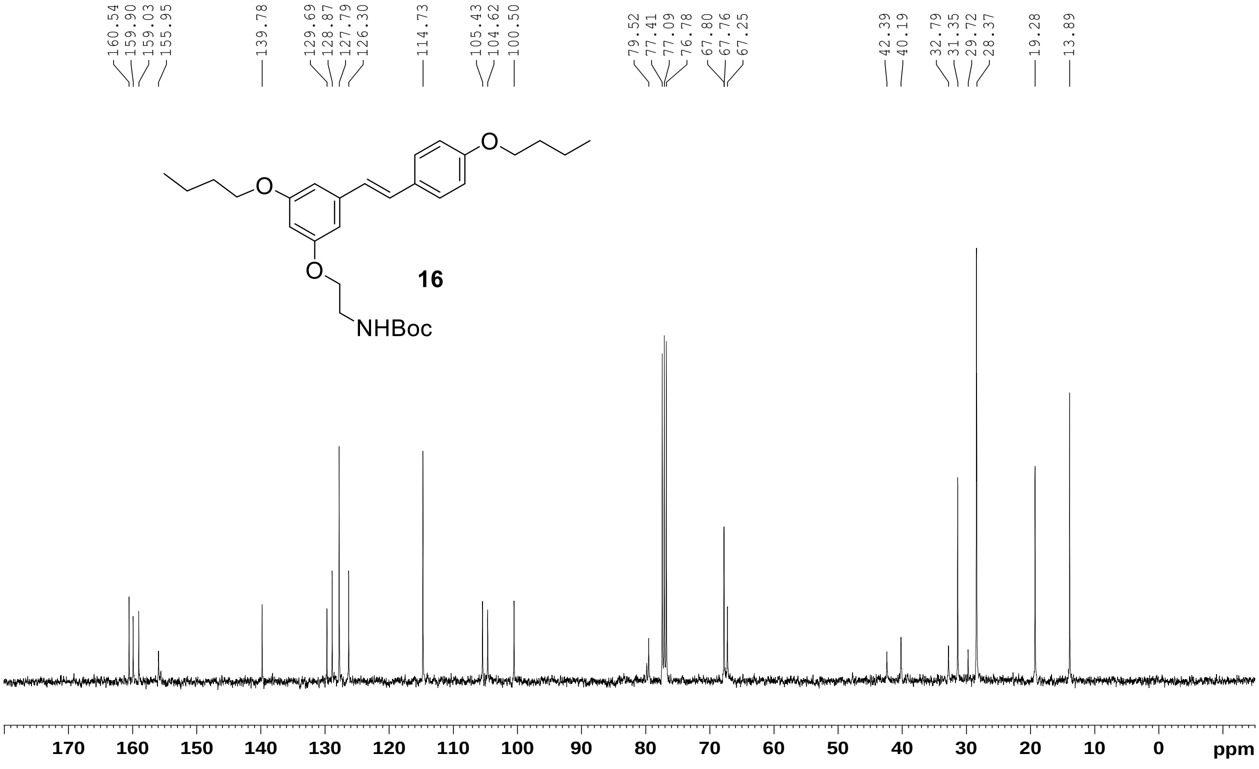
160.54 159.90 159.03 155.95 139.78 129.69 128.87 127.79 126.30 114.73 105.43 104.62 100.50 79.52 77.41 77.09 76.78 67.80 67.76 67.25 42.39 40.19 32.79 31.35 29.72 28.37 19.28 13.89
O
O
O
NHBoc
16
170 160 150 140 130 120 110 100 90 80 70 60 50 40 30 20 10 0 ppm

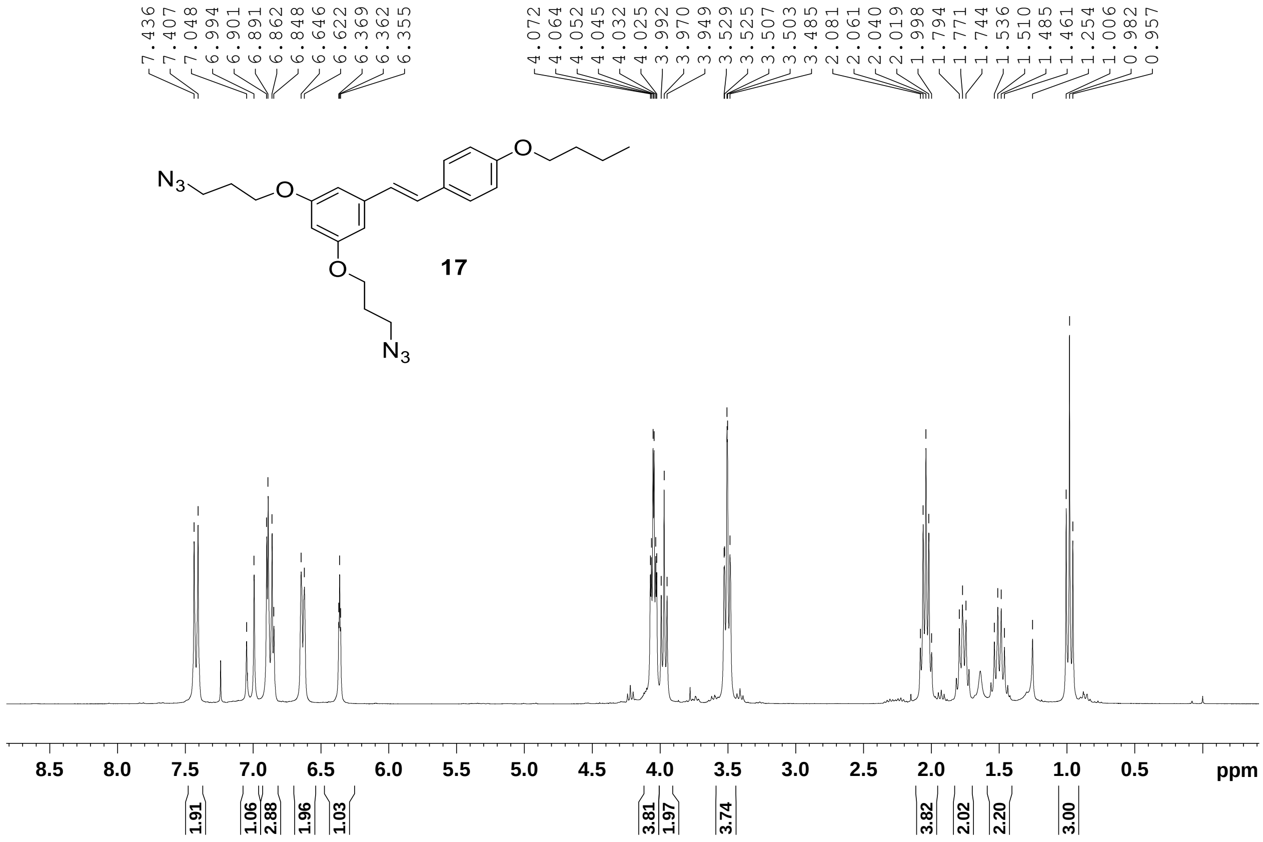
7.436 7.407 7.048 6.994 6.901 6.891 6.862 6.848 6.646 6.622 6.369 6.362 6.355
4.072 4.064 4.052 4.045 4.032 4.025 3.992 3.970 3.949 3.529 3.525 3.507 3.503 3.485 2.081 2.061 2.040 2.019 1.998 1.794 1.771 1.744 1.536 1.510 1.485 1.461 1.254 1.006 0.982 0.957
N3
O
O
N3
O
17
8.5 8.0 7.5 7.0 6.5 6.0 5.5 5.0 4.5 4.0 3.5 3.0 2.5 2.0 1.5 1.0 0.5 ppm
1.91 1.06 2.88 1.96 1.03 3.81 1.97 3.74 3.82 2.02 2.20 3.00

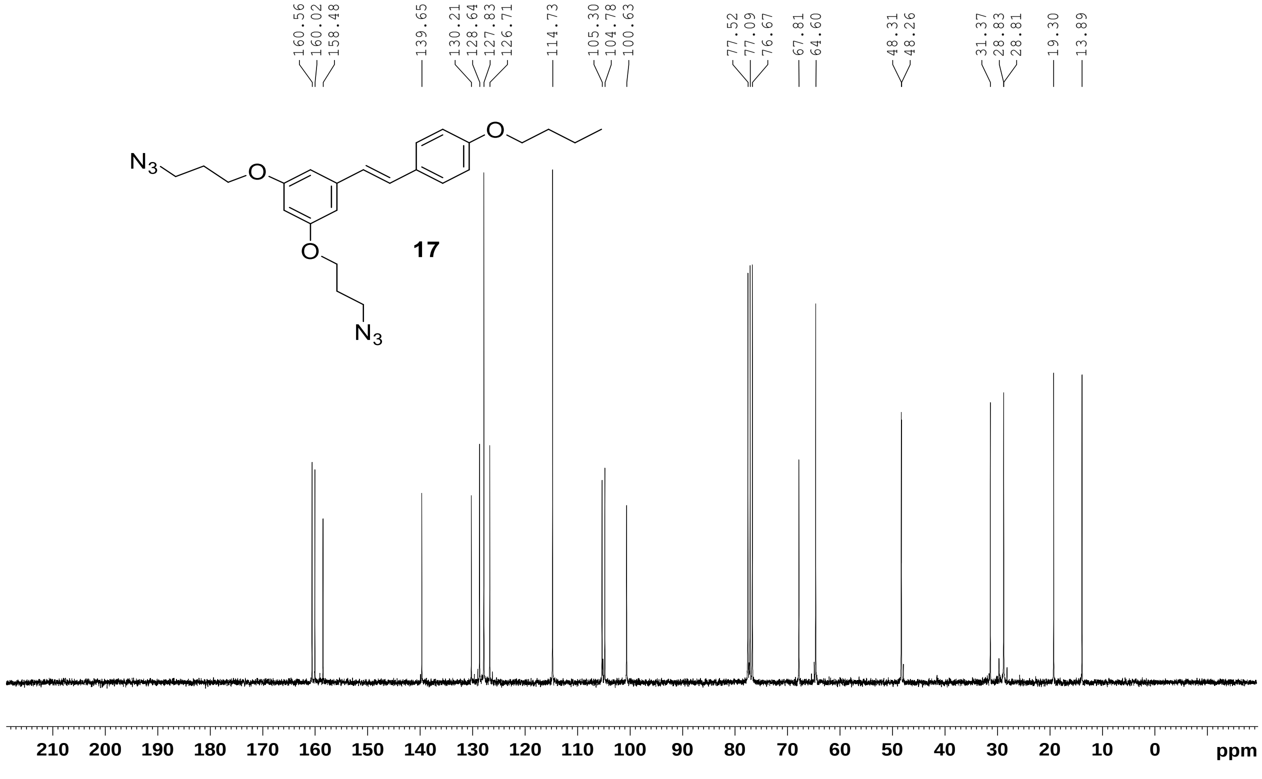
160.56 160.02 158.48 139.65 130.21 128.64 127.83 126.71 114.73 105.30 104.78 100.63 77.52 77.09 76.67 67.81 64.60 48.31 48.26 31.37 28.83 28.81 19.30 13.89
N3
O
O
N3
O
17
210 200 190 180 170 160 150 140 130 120 110 100 90 80 70 60 50 40 30 20 10 0 ppm

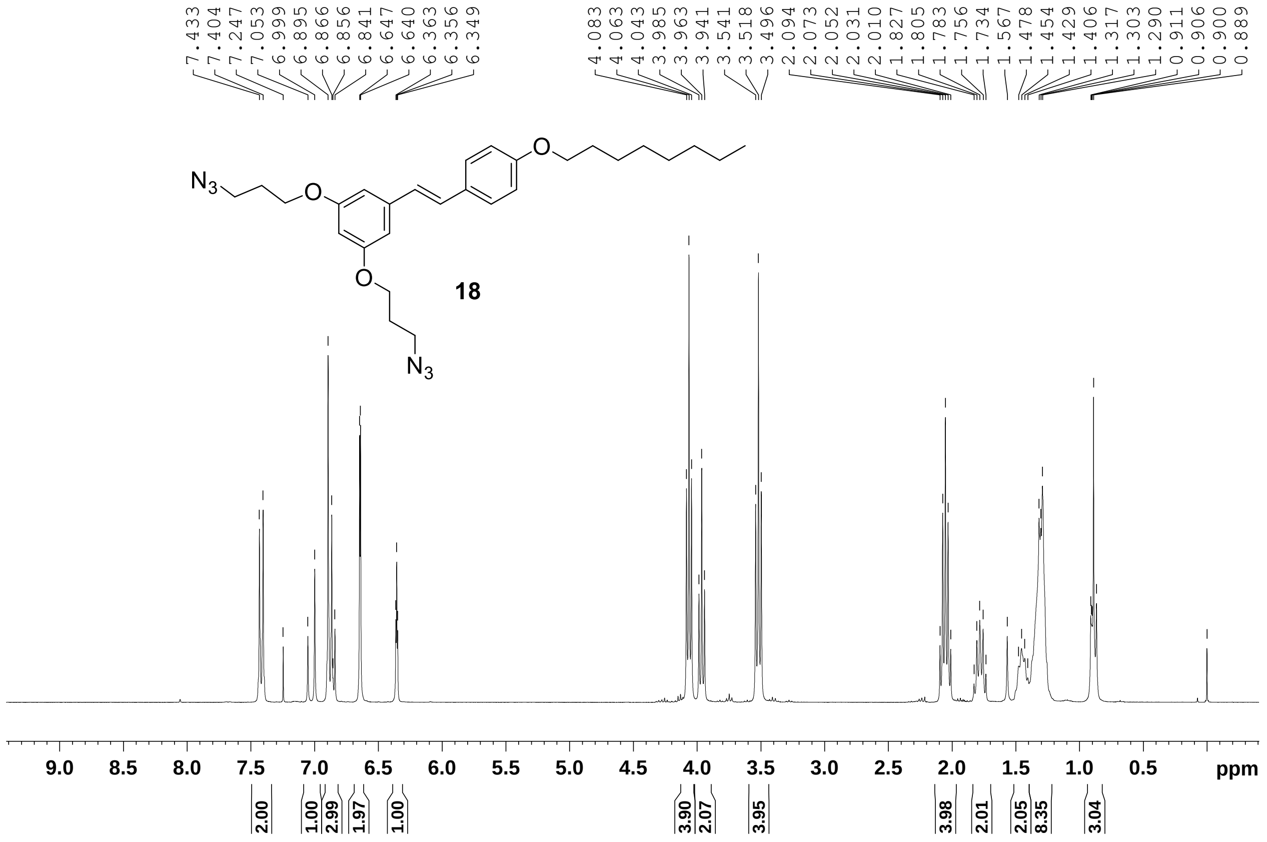

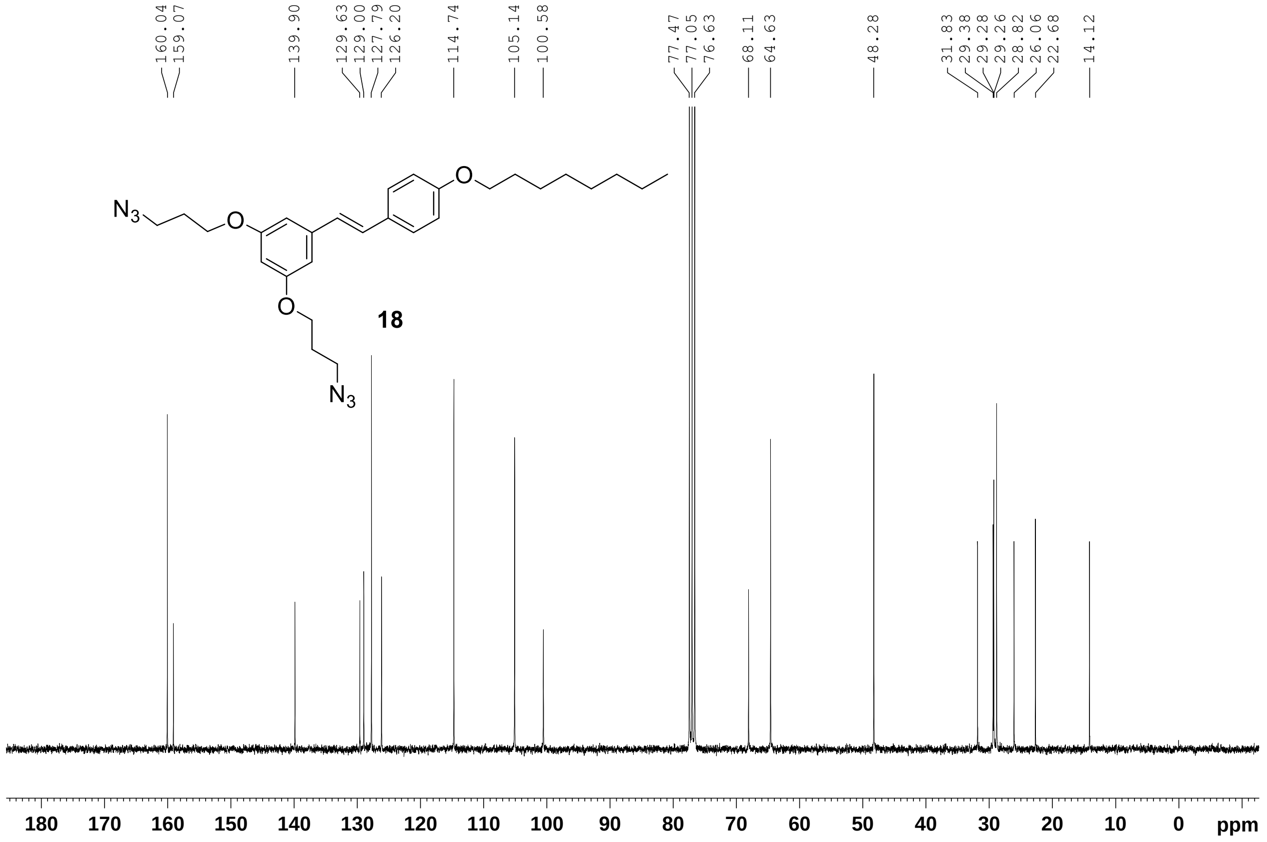

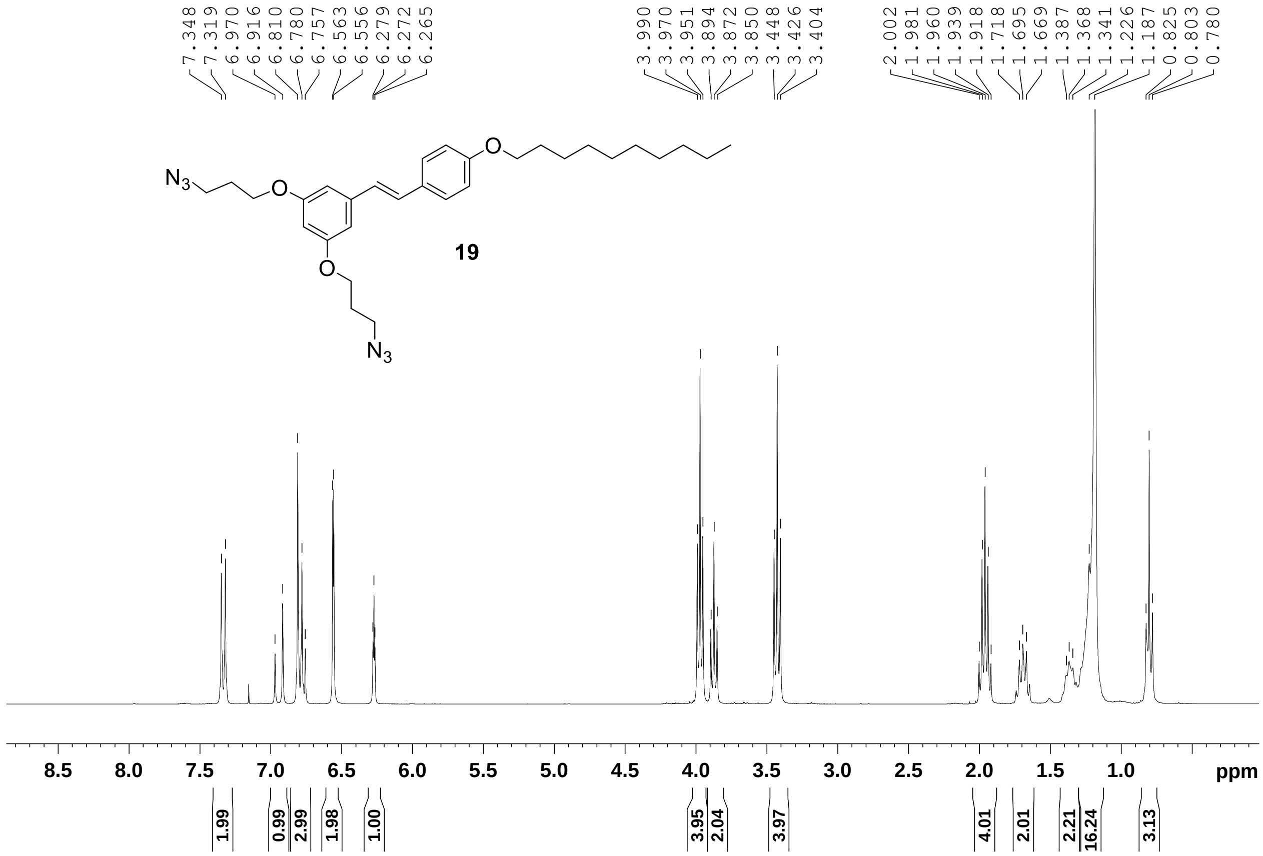
7.348 7.319 6.970 6.916 6.810 6.780 6.757 6.563 6.556 6.279 6.272 6.265
3.990 3.970 3.951 3.894 3.872 3.850 3.448 3.426 3.404
2.002 1.981 1.960 1.939 1.918 1.718 1.695 1.669 1.387 1.368 1.341 1.226 1.187 0.825 0.803 0.780
N3
O
19
ppm
1.99 0.99 2.99 1.98 1.00 3.95 2.04 3.97 4.01 2.01 2.21 16.24 3.13

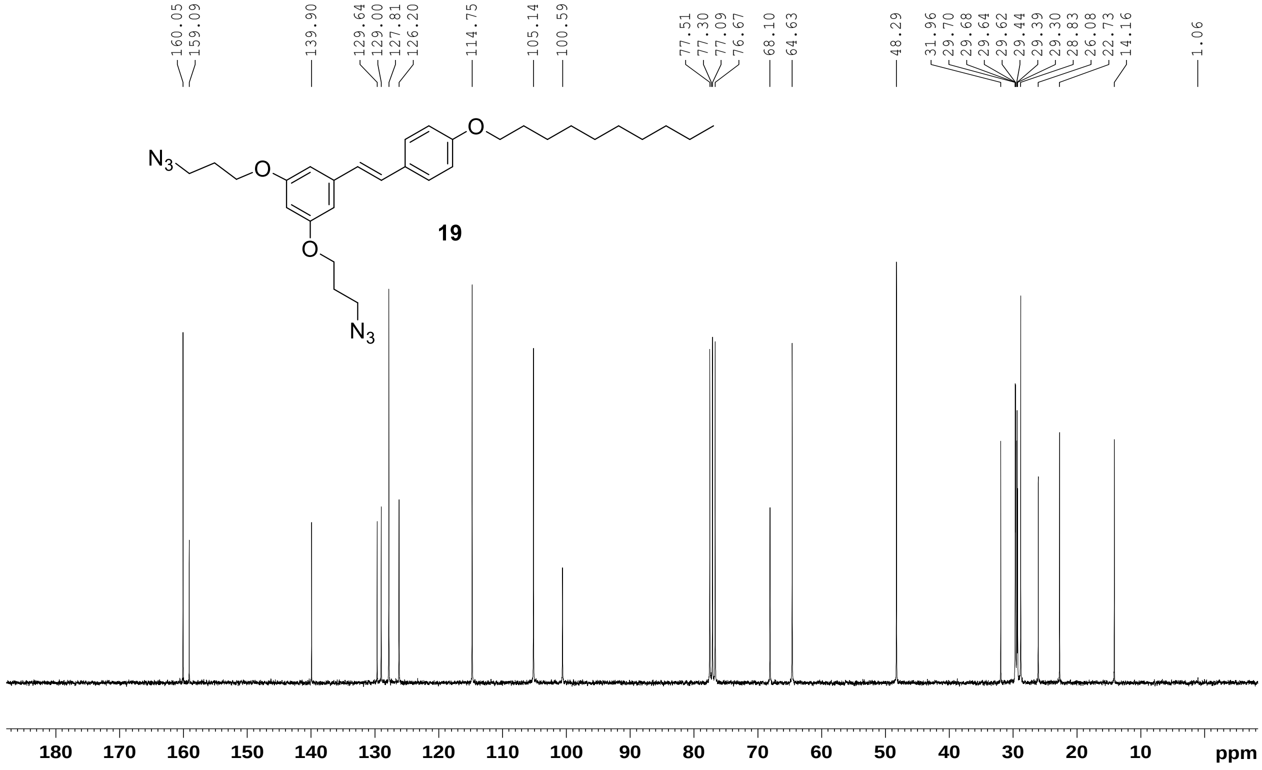
160.05 159.09 139.90 129.64 129.00 127.81 126.20 114.75 105.14 100.59
77.51 77.30 77.09 76.67 68.10 64.63 48.29
31.96 29.70 29.68 29.64 29.62 29.44 29.39 29.30 28.83 26.08 22.73 14.16 1.06
N3
O
19
ppm

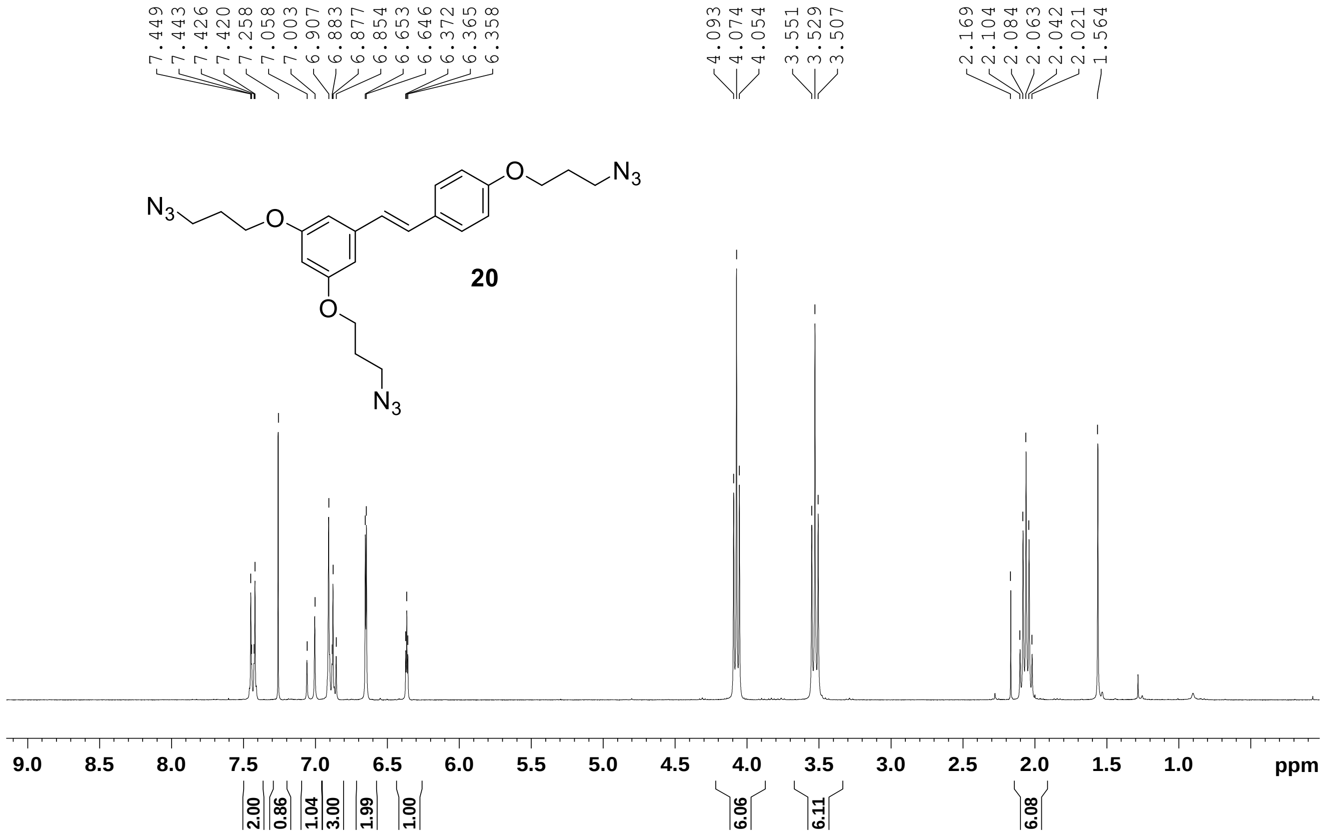
7.449
7.443
7.426
7.420
7.258
7.058
7.003
6.907
6.883
6.877
6.854
6.653
6.646
6.372
6.365
6.358
4.093
4.074
4.054
3.551
3.529
3.507
2.169
2.104
2.084
2.063
2.042
2.021
1.564
N_3
O
20
9.0 8.5 8.0 7.5 7.0 6.5 6.0 5.5 5.0 4.5 4.0 3.5 3.0 2.5 2.0 1.5 1.0 ppm
2.00
0.86
1.04
3.00
1.99
1.00
6.06
6.11
6.08

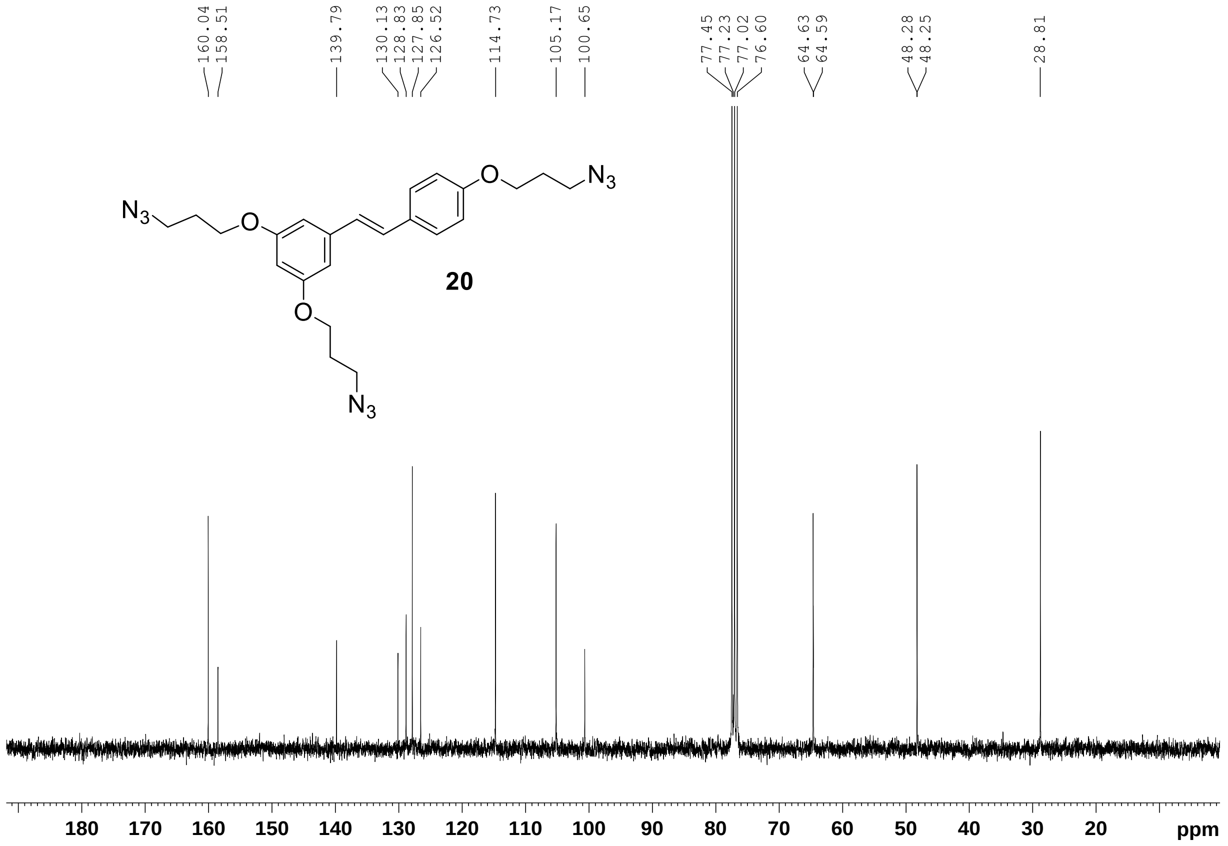
160.04
158.51
139.79
130.13
128.83
127.85
126.52
114.73
105.17
100.65
77.45
77.23
77.02
76.60
64.63
64.59
48.28
48.25
28.81
N_3
O
20
180 170 160 150 140 130 120 110 100 90 80 70 60 50 40 30 20 ppm

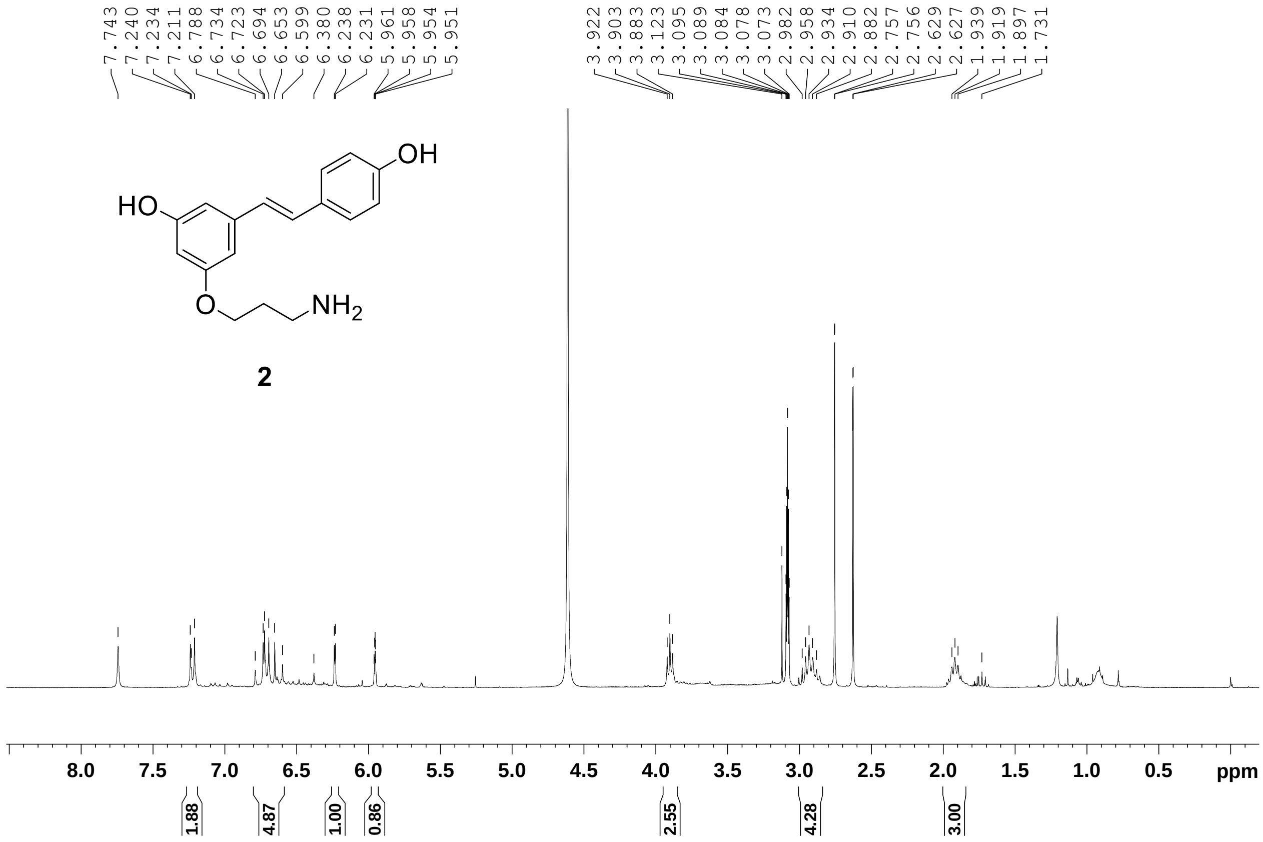

7.743
7.240
7.234
7.211
6.788
6.734
6.723
6.694
6.653
6.599
6.380
6.238
6.231
5.961
5.958
5.954
5.951
3.922
3.903
3.883
3.123
3.095
3.089
3.084
3.078
3.073
2.982
2.958
2.934
2.910
2.882
2.757
2.756
2.629
2.627
1.939
1.919
1.897
1.731
OH
HO
O
NH2
2
8.0 7.5 7.0 6.5 6.0 5.5 5.0 4.5 4.0 3.5 3.0 2.5 2.0 1.5 1.0 0.5 ppm
1.88
4.87
1.00
0.86
2.55
4.28
3.00

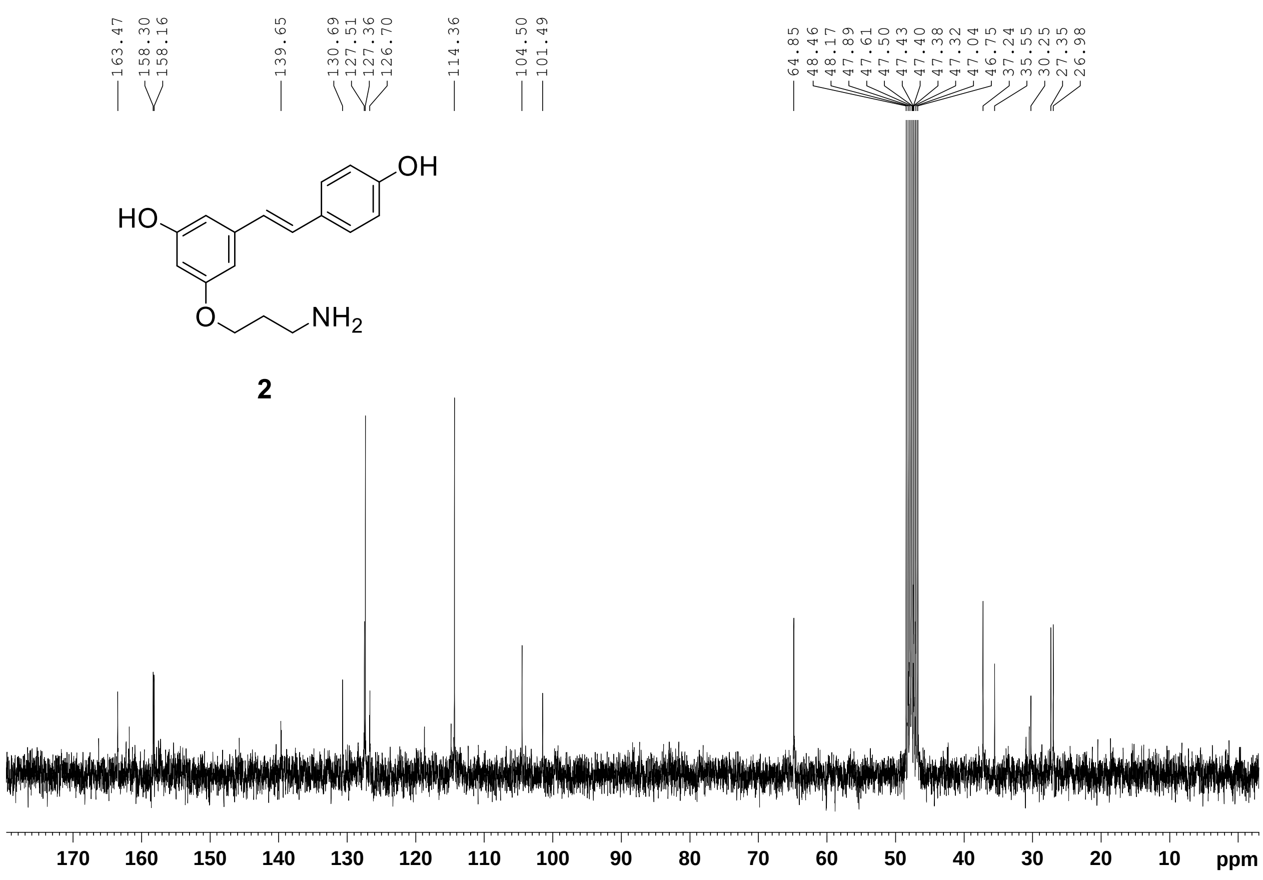

163.47
158.30
158.16
139.65
130.69
127.51
127.36
126.70
114.36
104.50
101.49
64.85
48.46
48.17
47.89
47.61
47.50
47.43
47.40
47.38
47.32
47.04
46.75
37.24
35.55
30.25
27.35
26.98
OH
HO
O
NH2
2
170 160 150 140 130 120 110 100 90 80 70 60 50 40 30 20 10 ppm

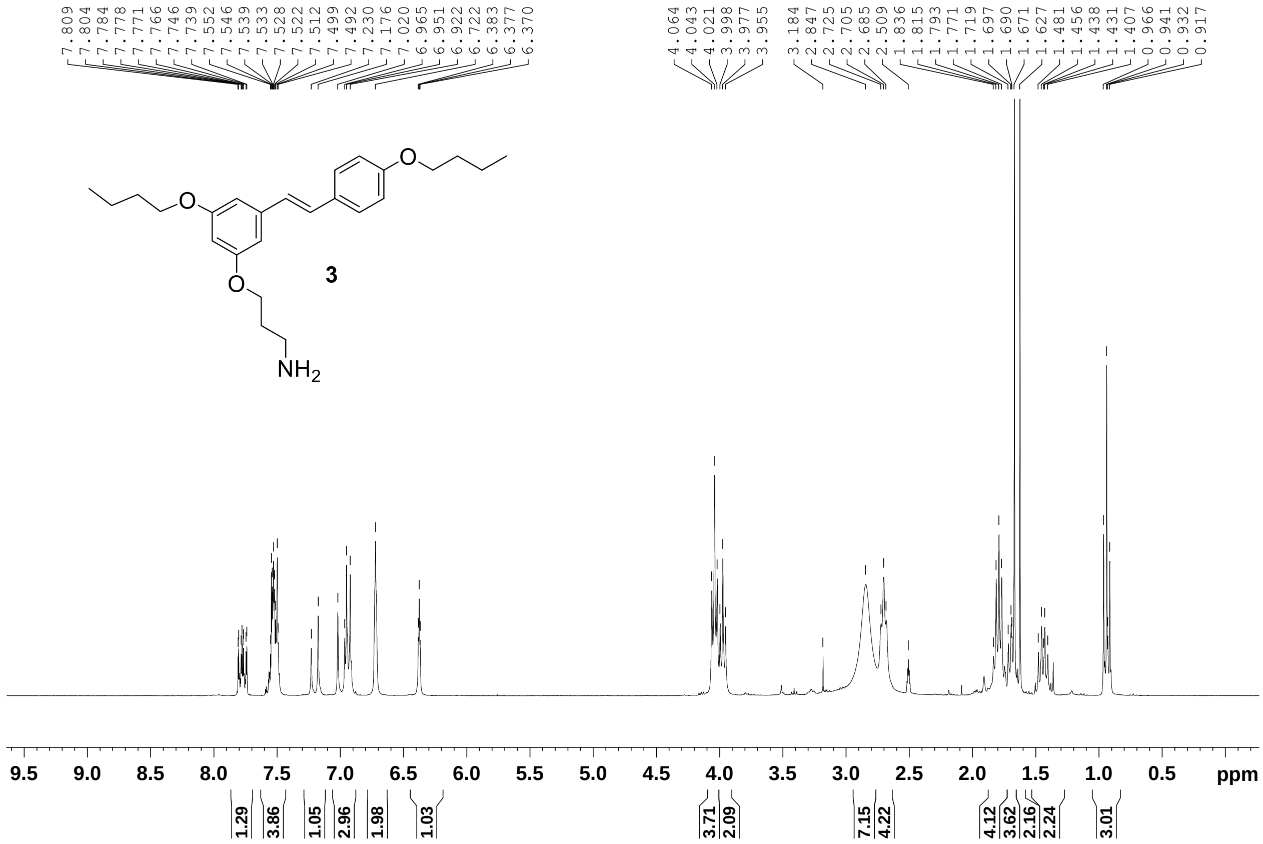

7.809 7.804 7.784 7.778 7.771 7.766 7.746 7.739 7.552 7.546 7.539 7.533 7.528 7.522 7.512 7.499 7.492 7.230 7.176 7.020 6.965 6.951 6.922 6.722 6.383 6.377 6.370
4.064 4.043 4.021 3.998 3.977 3.955 3.184 2.847 2.725 2.705 2.685 2.509 1.836 1.815 1.793 1.771 1.719 1.697 1.690 1.671 1.627 1.481 1.456 1.438 1.431 1.407 0.966 0.941 0.932 0.917
O
O
O
NH2
3
9.5 9.0 8.5 8.0 7.5 7.0 6.5 6.0 5.5 5.0 4.5 4.0 3.5 3.0 2.5 2.0 1.5 1.0 0.5 ppm
1.29 3.86 1.05 2.96 1.98 1.03 3.71 2.09 7.15 4.22 4.12 3.62 2.16 2.24 3.01

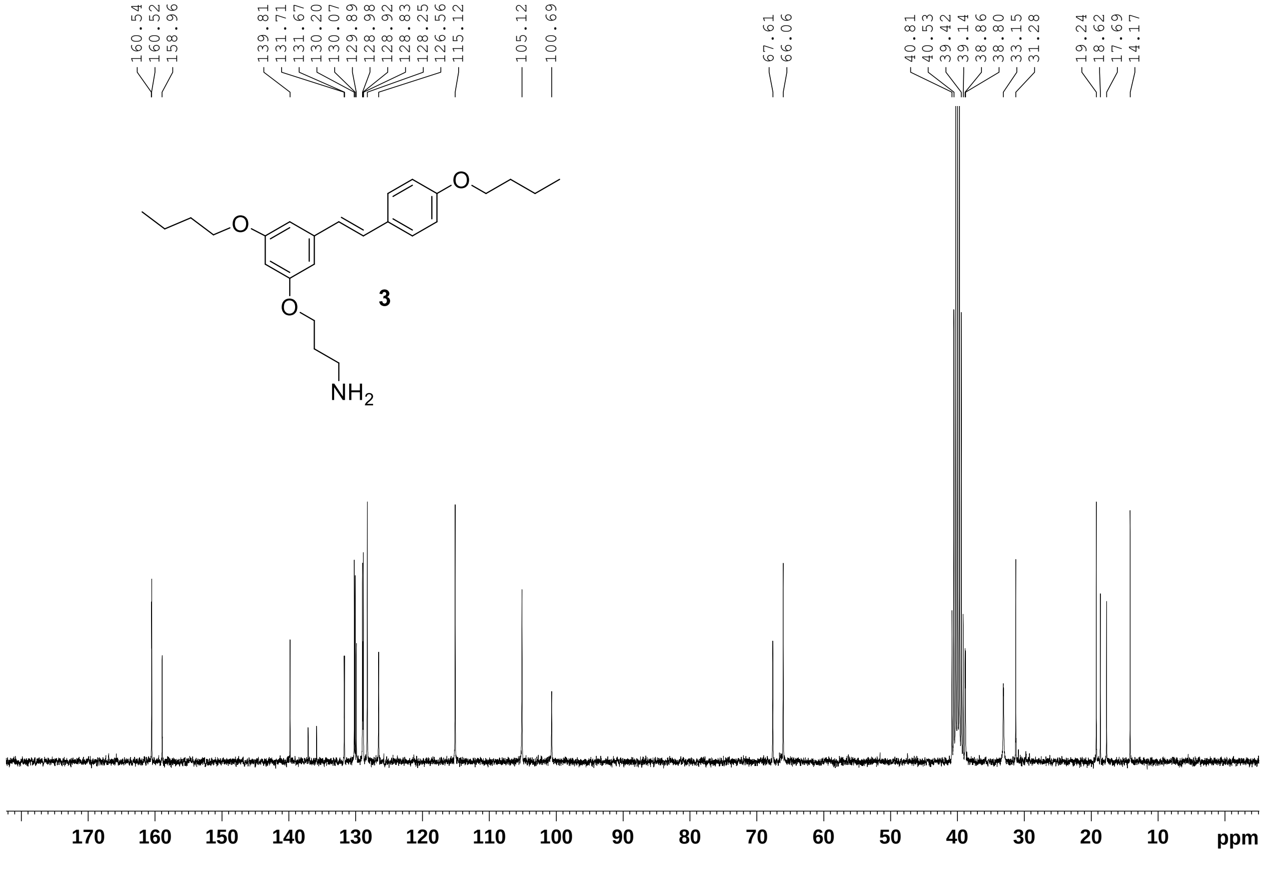

160.54 160.52 158.96 139.81 131.71 131.67 130.20 130.07 129.89 128.98 128.92 128.83 128.25 126.56 115.12 105.12 100.69
67.61 66.06 40.81 40.53 39.42 39.14 38.86 38.80 33.15 31.28 19.24 18.62 17.69 14.17
O
O
O
NH2
3
170 160 150 140 130 120 110 100 90 80 70 60 50 40 30 20 10 ppm

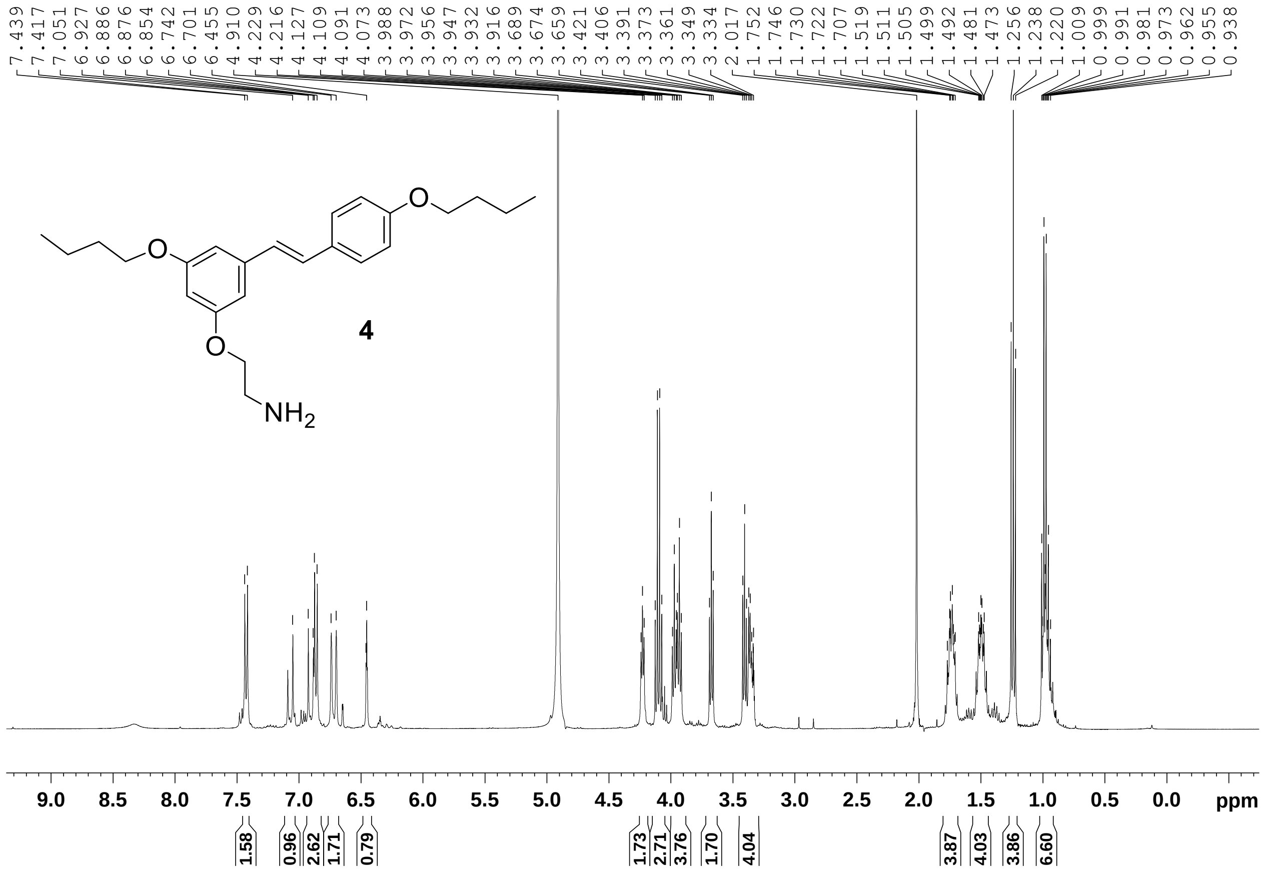

7.439 7.417 7.051 6.927 6.886 6.876 6.854 6.742 6.701 6.455 4.910 4.229 4.216 4.127 4.109 4.091 4.073 3.988 3.972 3.956 3.947 3.932 3.916 3.689 3.674 3.659 3.421 3.406 3.391 3.373 3.361 3.349 3.334 2.017 1.752 1.746 1.730 1.722 1.707 1.519 1.511 1.505 1.499 1.492 1.481 1.473 1.256 1.238 1.220 1.009 0.999 0.991 0.981 0.973 0.962 0.955 0.938
O
O
O
NH2
4
9.0 8.5 8.0 7.5 7.0 6.5 6.0 5.5 5.0 4.5 4.0 3.5 3.0 2.5 2.0 1.5 1.0 0.5 0.0 ppm
1.58 0.96 2.62 1.71 0.79 1.73 2.71 3.76 1.70 4.04 3.87 4.03 3.86 6.60

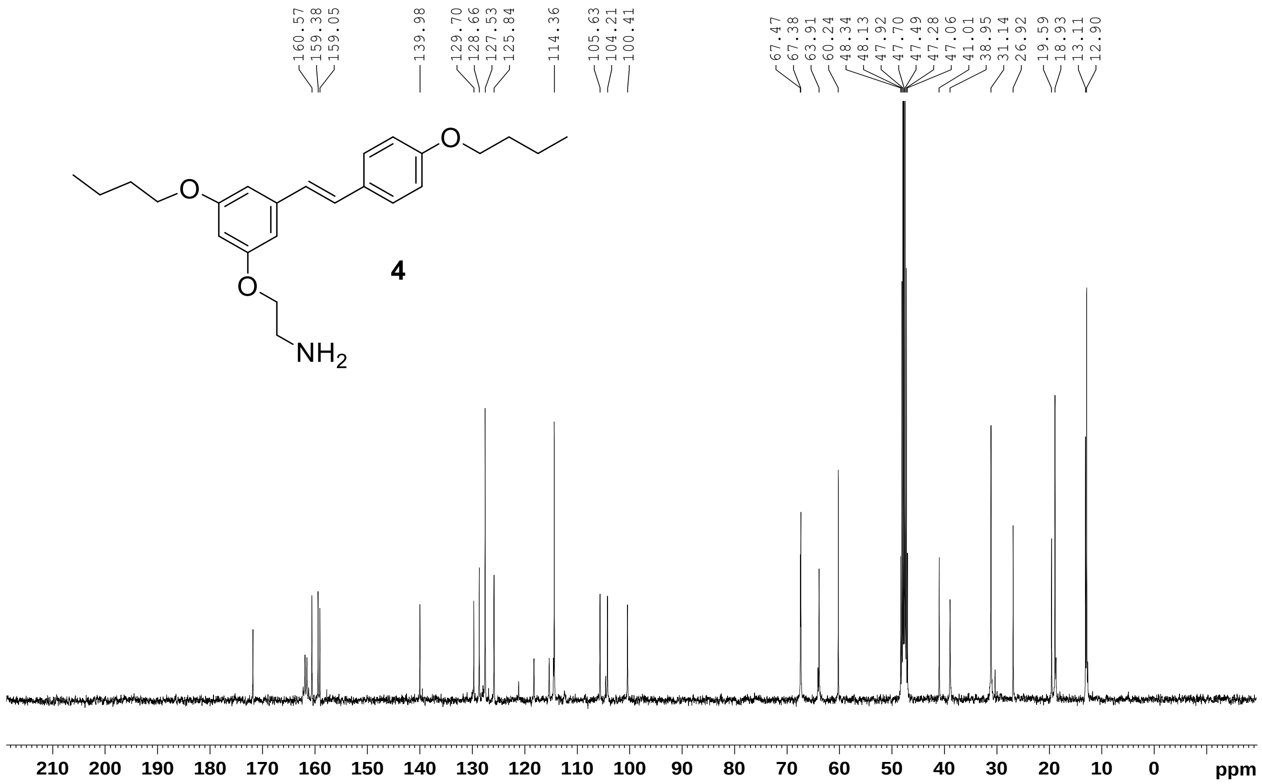

160.57 159.38 159.05 139.98 129.70 128.66 127.53 125.84 114.36 105.63 104.21 100.41 67.47 67.38 63.91 60.24 48.34 48.13 47.92 47.70 47.49 47.28 47.06 41.01 38.95 31.14 26.92 19.59 18.93 13.11 12.90
O
O
O
NH2
4
210 200 190 180 170 160 150 140 130 120 110 100 90 80 70 60 50 40 30 20 10 0 ppm

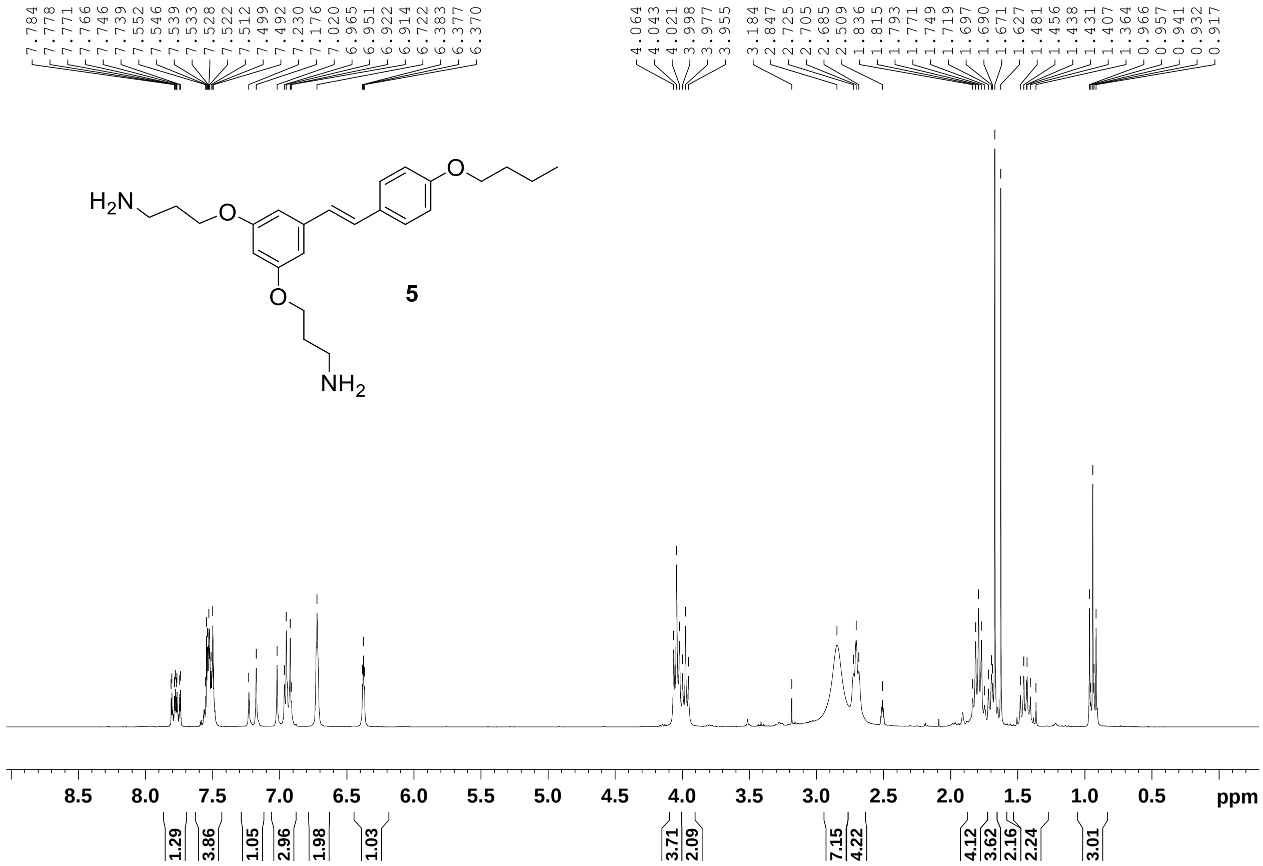
7.784
7.778
7.771
7.766
7.746
7.739
7.552
7.546
7.539
7.533
7.528
7.522
7.512
7.499
7.492
7.230
7.176
7.020
6.965
6.951
6.922
6.914
6.722
6.383
6.377
6.370
4.064
4.043
4.021
3.998
3.977
3.955
3.184
2.847
2.725
2.705
2.685
2.509
1.836
1.815
1.793
1.771
1.749
1.719
1.697
1.690
1.671
1.627
1.481
1.456
1.438
1.431
1.407
1.364
0.966
0.957
0.941
0.932
0.917
H2N
O
O
O
NH2
5
8.5
8.0
7.5
7.0
6.5
6.0
5.5
5.0
4.5
4.0
3.5
3.0
2.5
2.0
1.5
1.0
0.5
ppm
1.29
3.86
1.05
2.96
1.98
1.03
3.71
2.09
7.15
4.22
4.12
3.62
2.16
2.24
3.01

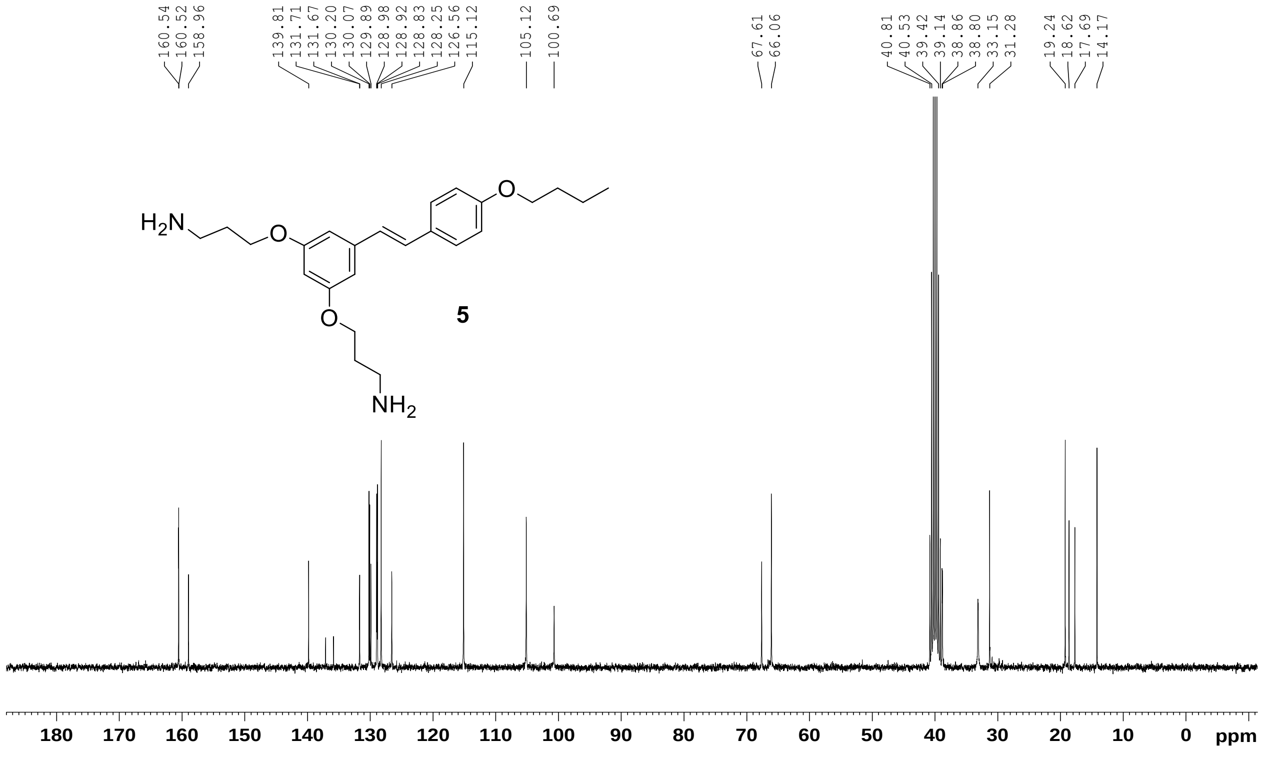
160.54
160.52
158.96
139.81
131.71
131.67
130.20
130.07
129.89
128.98
128.92
128.83
128.25
126.56
115.12
105.12
100.69
67.61
66.06
40.81
40.53
39.42
39.14
38.86
38.80
33.15
31.28
19.24
18.62
17.69
14.17
H2N
O
O
O
NH2
5
180
170
160
150
140
130
120
110
100
90
80
70
60
50
40
30
20
10
0
ppm

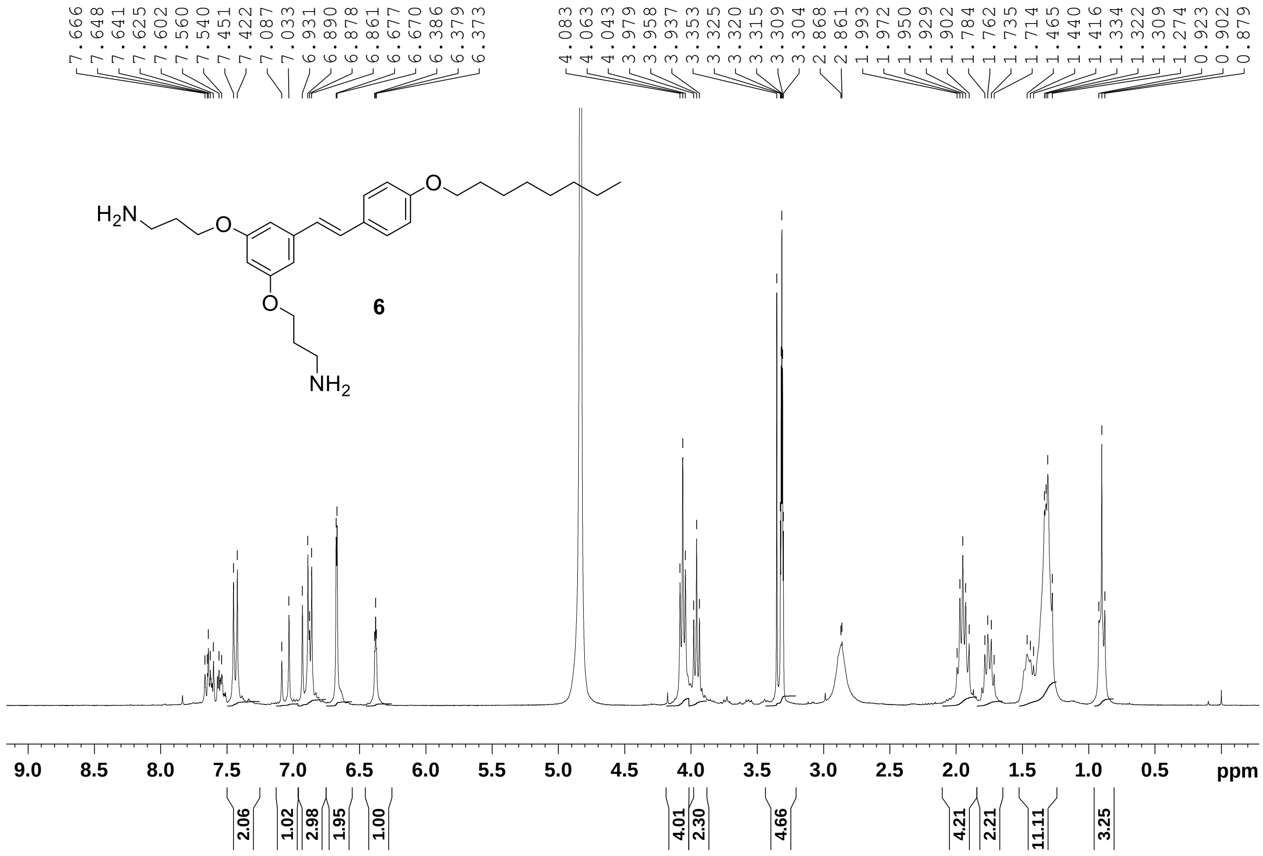
7.666
7.648
7.641
7.625
7.602
7.560
7.540
7.451
7.422
7.087
7.033
6.931
6.890
6.878
6.861
6.677
6.670
6.386
6.379
6.373
4.083
4.063
4.043
3.979
3.958
3.937
3.353
3.325
3.320
3.315
3.309
3.304
2.868
2.861
1.993
1.972
1.950
1.929
1.902
1.784
1.762
1.735
1.714
1.465
1.440
1.416
1.334
1.322
1.309
1.274
0.923
0.902
0.879
H2N
O
O
O
NH2
6
2.06
1.02
2.98
1.95
1.00
4.01
2.30
4.66
4.21
2.21
11.11
3.25
ppm

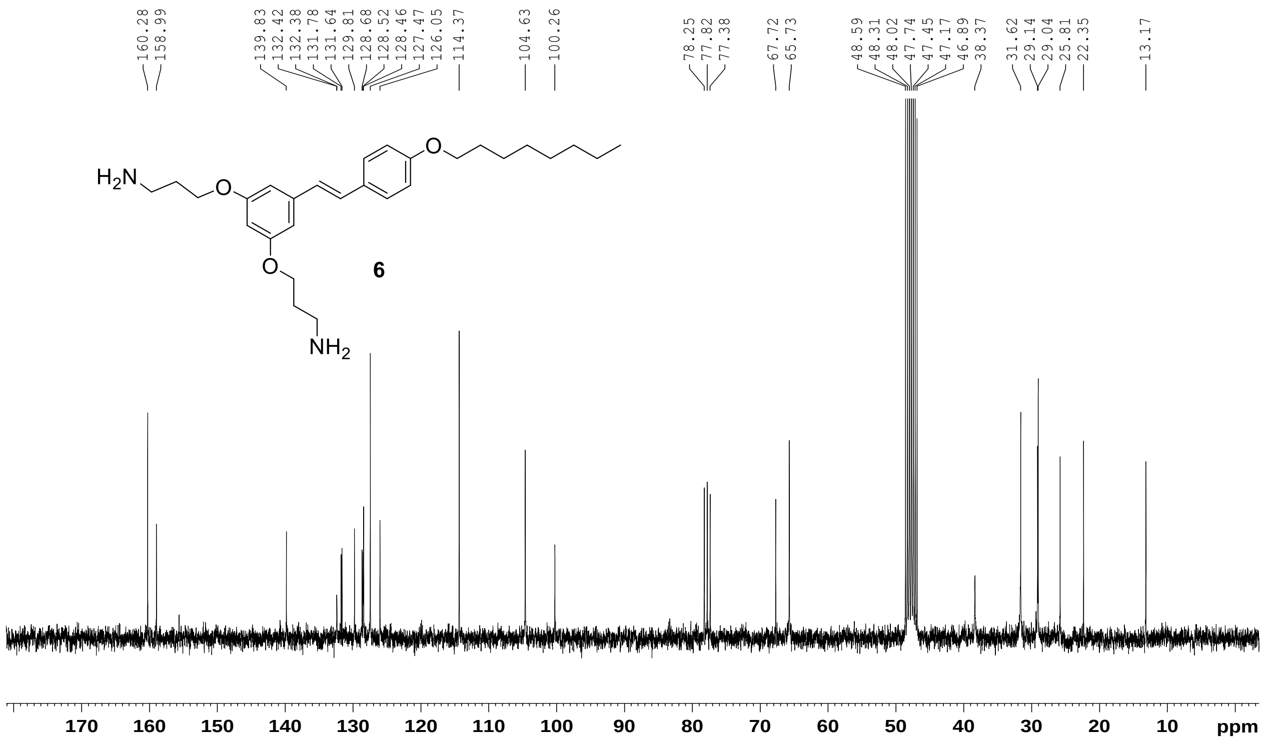
160.28
158.99
139.83
132.42
132.38
131.78
131.64
129.81
128.68
128.52
128.46
127.47
126.05
114.37
104.63
100.26
78.25
77.82
77.38
67.72
65.73
48.59
48.31
48.02
47.74
47.45
47.17
46.89
38.37
31.62
29.14
29.04
25.81
22.35
13.17
H2N
O
O
O
NH2
6
ppm

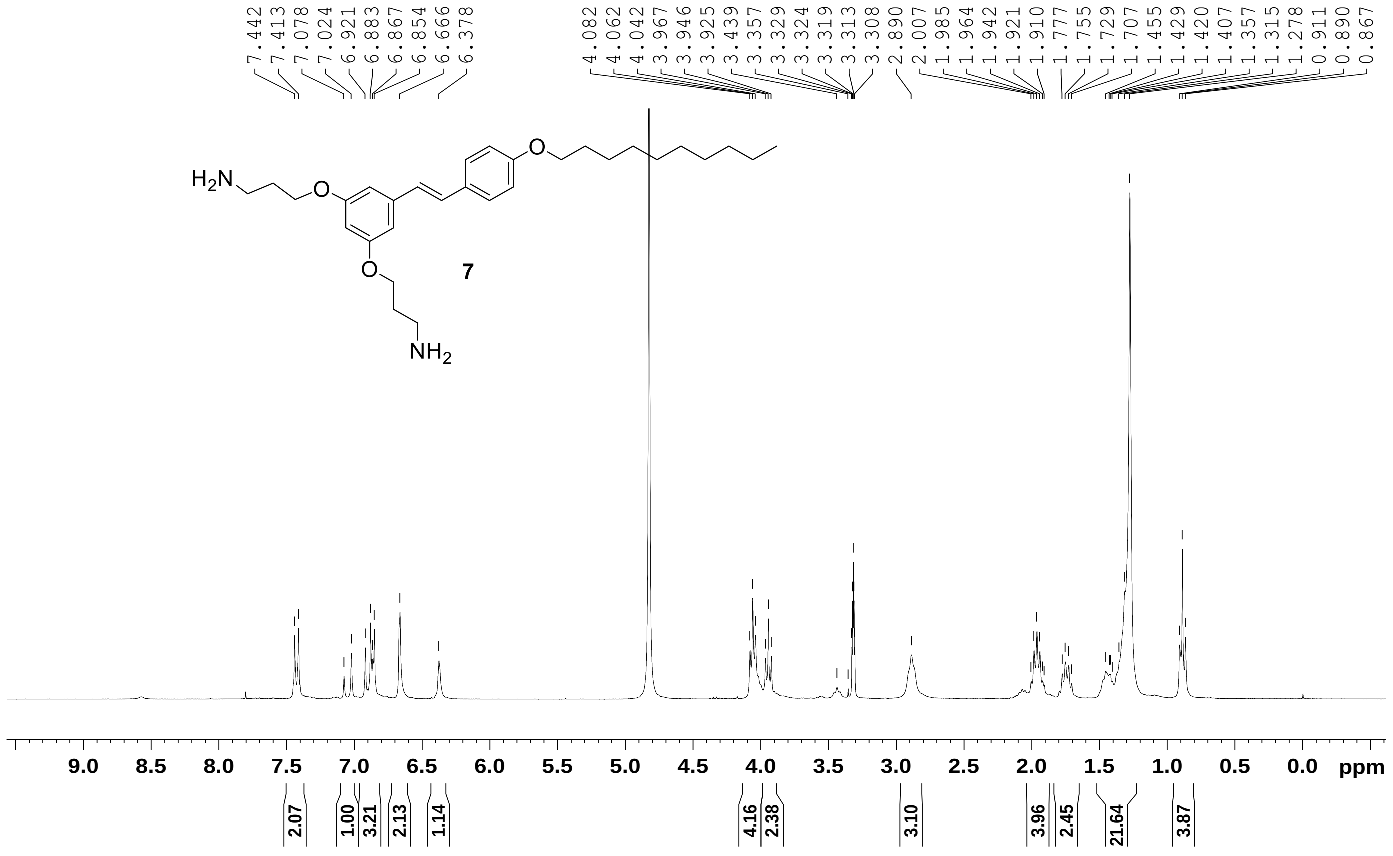

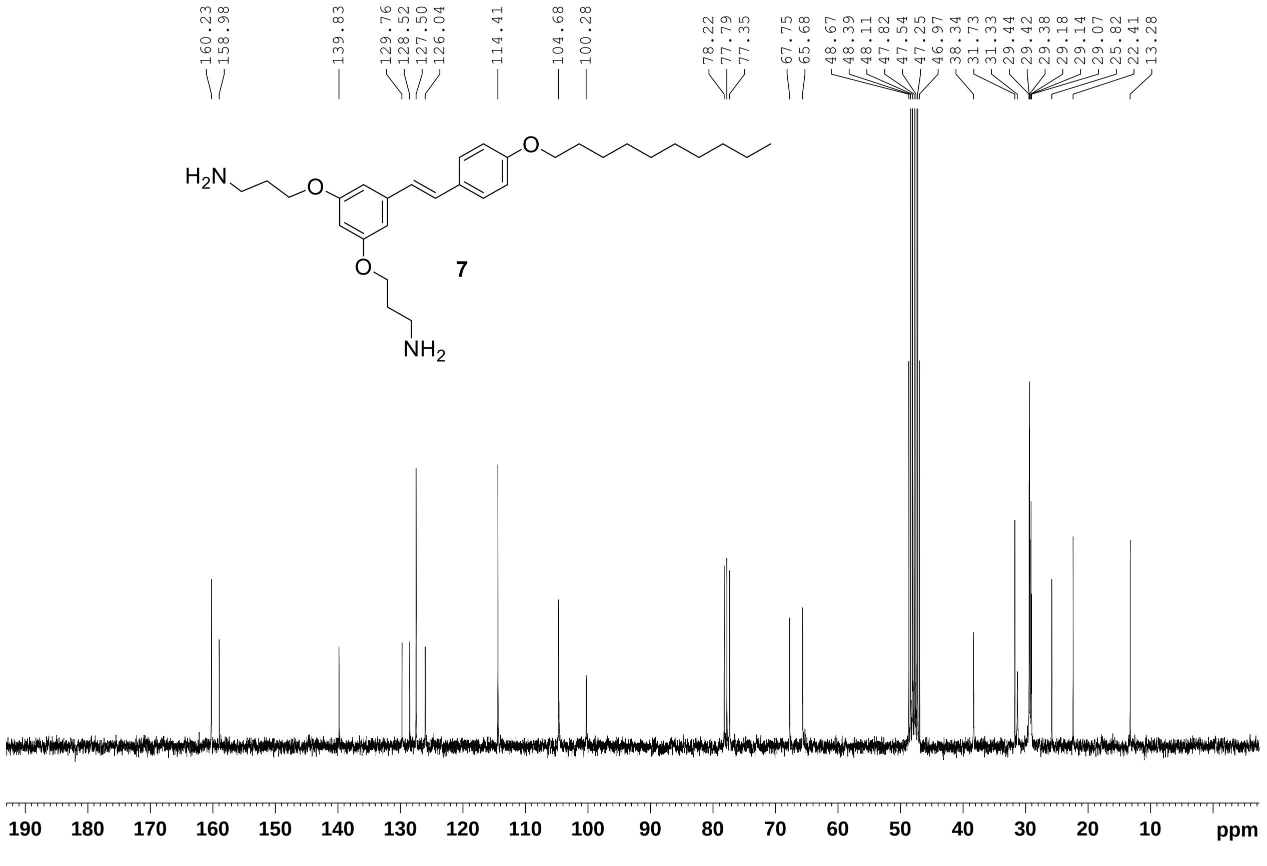

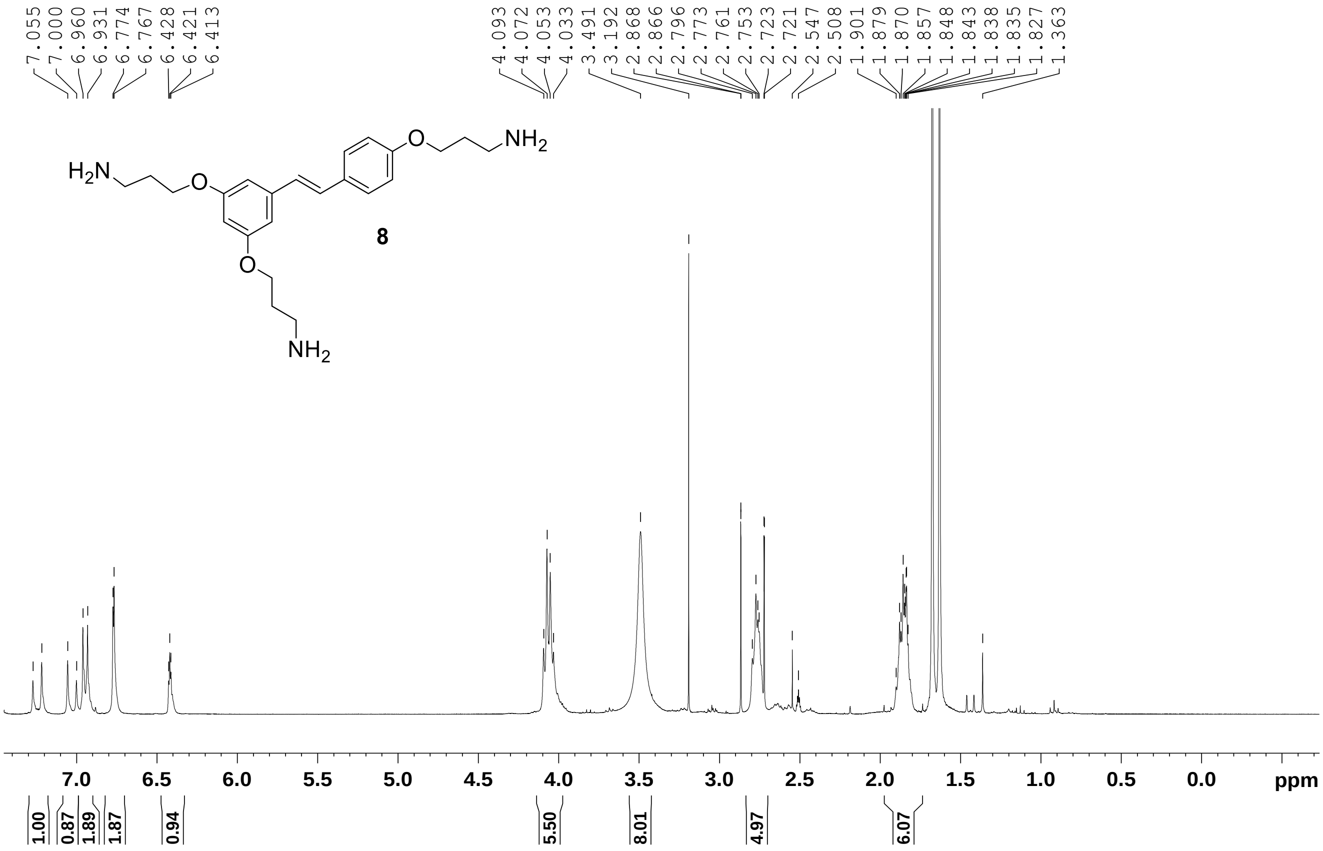
7.055
7.000
6.960
6.931
6.774
6.767
6.428
6.421
6.413
4.093
4.072
4.053
4.033
3.491
3.192
2.868
2.866
2.796
2.773
2.761
2.753
2.723
2.721
2.547
2.508
1.901
1.879
1.870
1.857
1.848
1.843
1.838
1.835
1.827
1.363
H_2N
NH_2
O
8
7.0 6.5 6.0 5.5 5.0 4.5 4.0 3.5 3.0 2.5 2.0 1.5 1.0 0.5 0.0 ppm
1.00
0.87
1.89
1.87
0.94
5.50
8.01
4.97
6.07

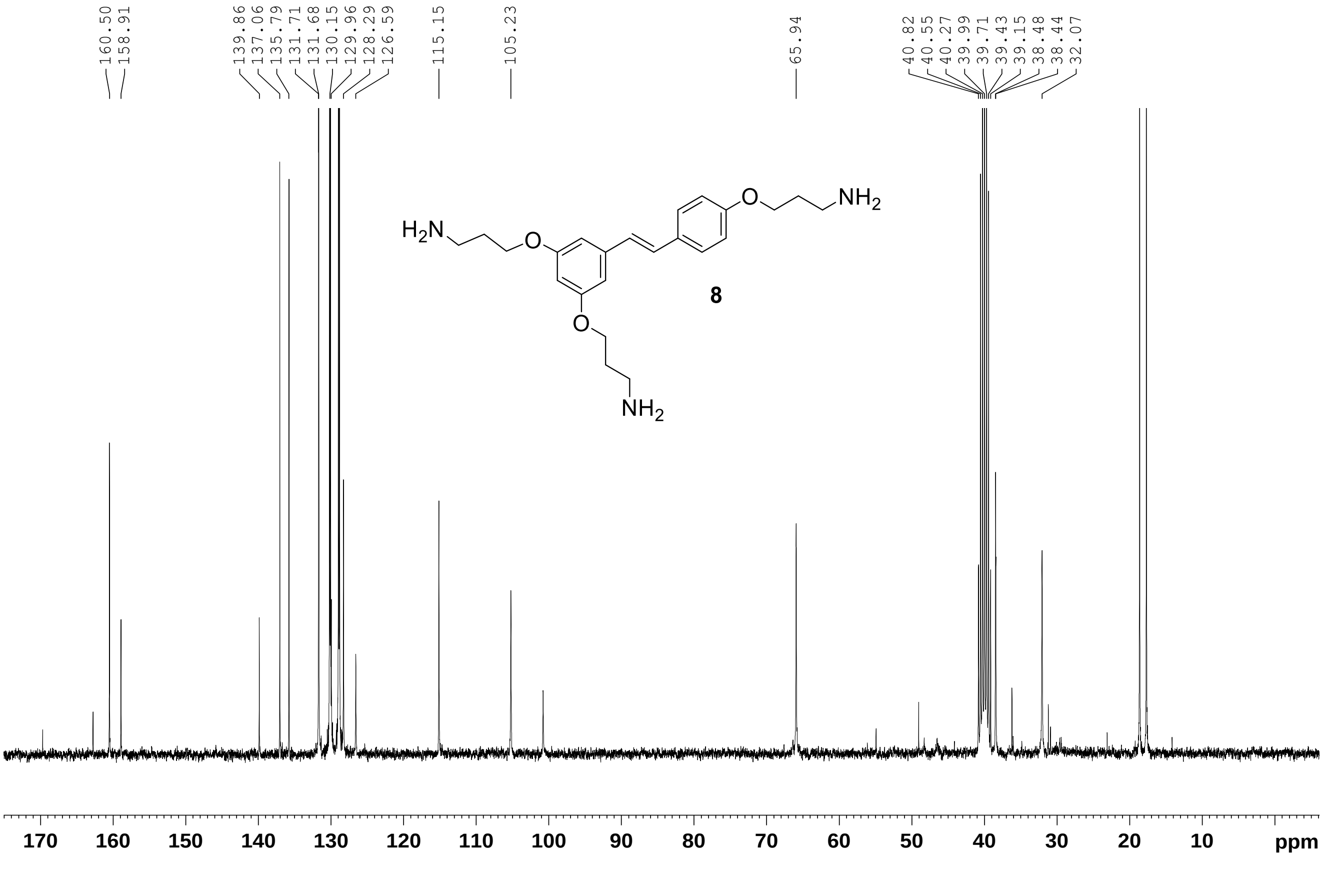
160.50
158.91
139.86
137.06
135.79
131.71
131.68
130.15
129.96
128.29
126.59
115.15
105.23
65.94
40.82
40.55
40.27
39.99
39.71
39.43
39.15
38.48
38.44
32.07
H_2N
NH_2
O
8
170 160 150 140 130 120 110 100 90 80 70 60 50 40 30 20 10 ppm

High resolution MS of final products

Compound **2** ($C_{17}H_{20}N0_3$ $[M+H]^+$)

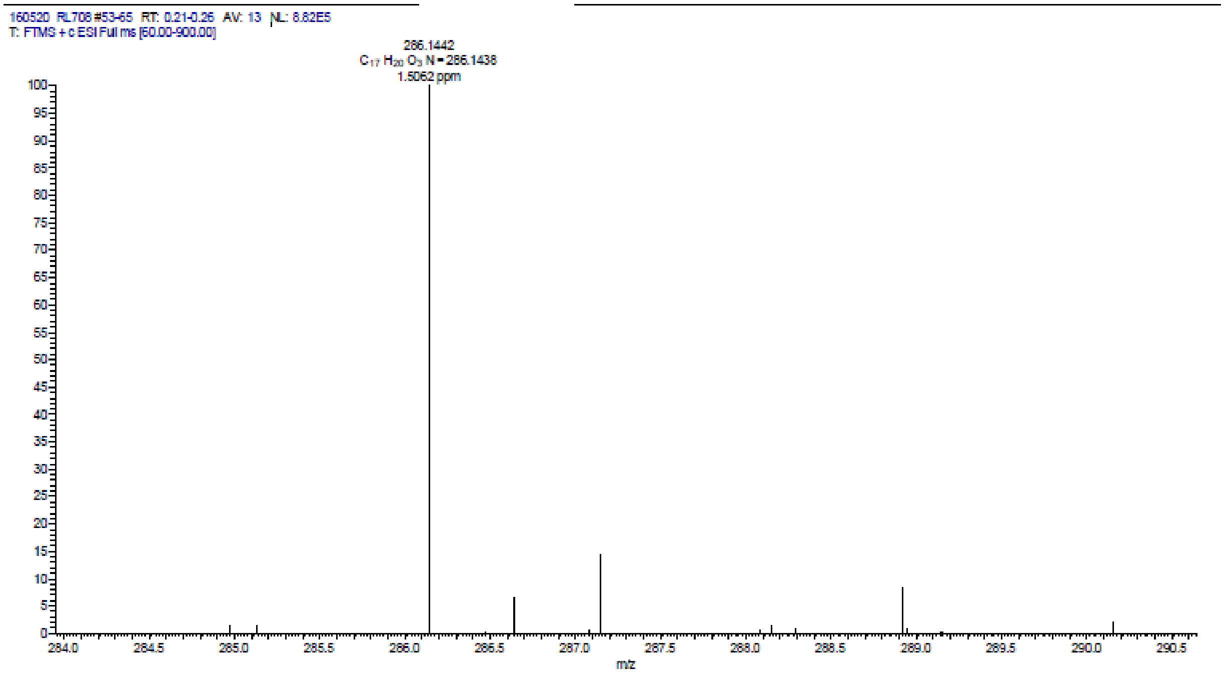

Compound **3** ($C_{25}H_{36}N0_3$ $[M+H]^+$):

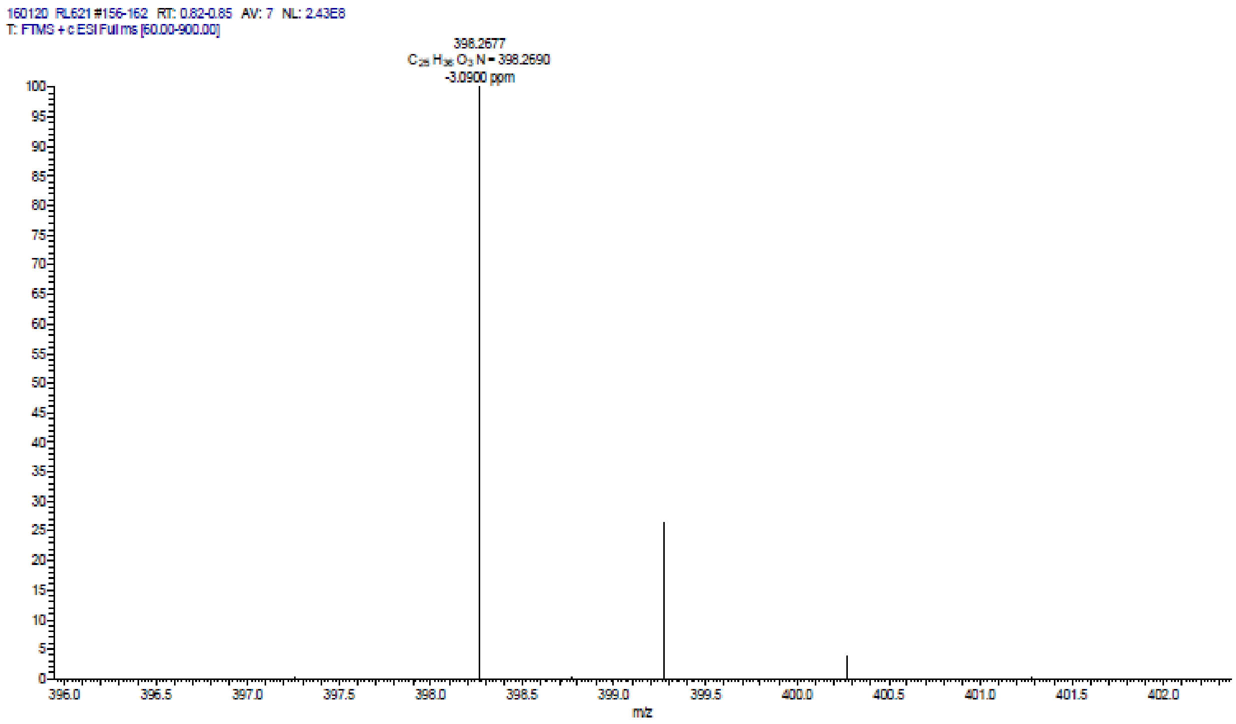

Compound **4** ($C_{24}H_{34}N0_3$ $[M+H]^+$):

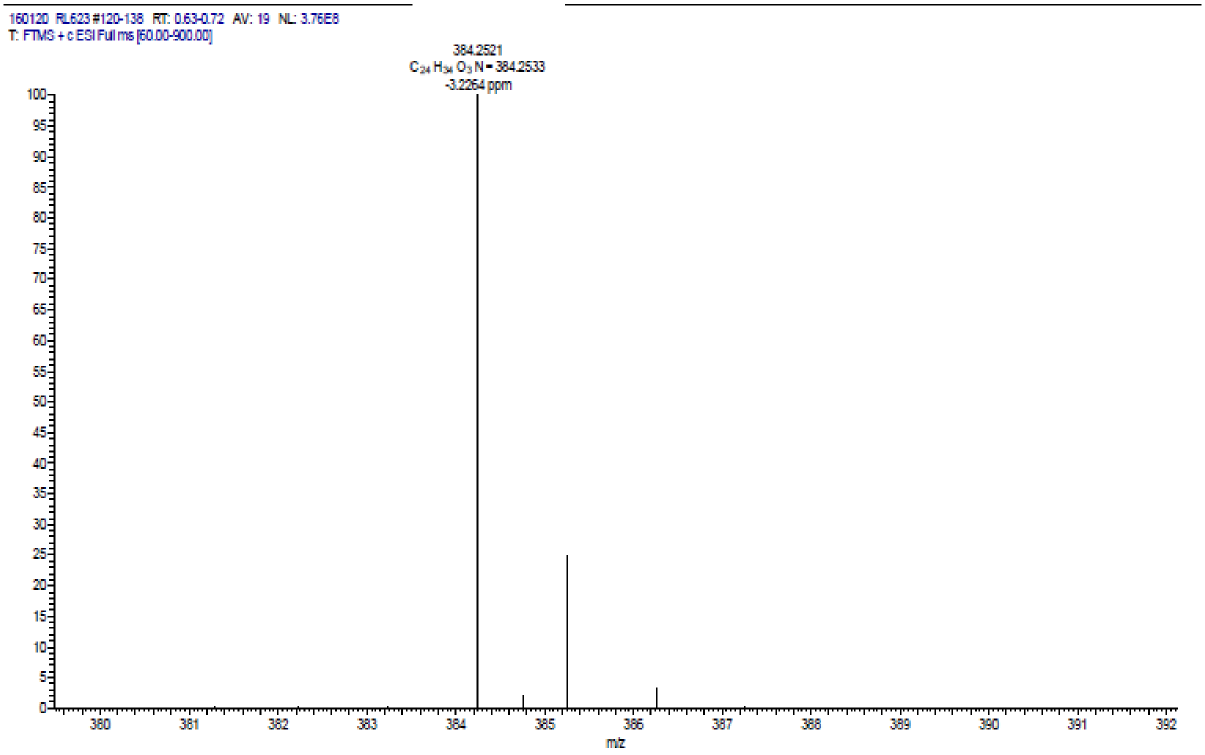

Compound **5** ($C_{24}H_{35}N_2O_3$ $[M+H]^+$):

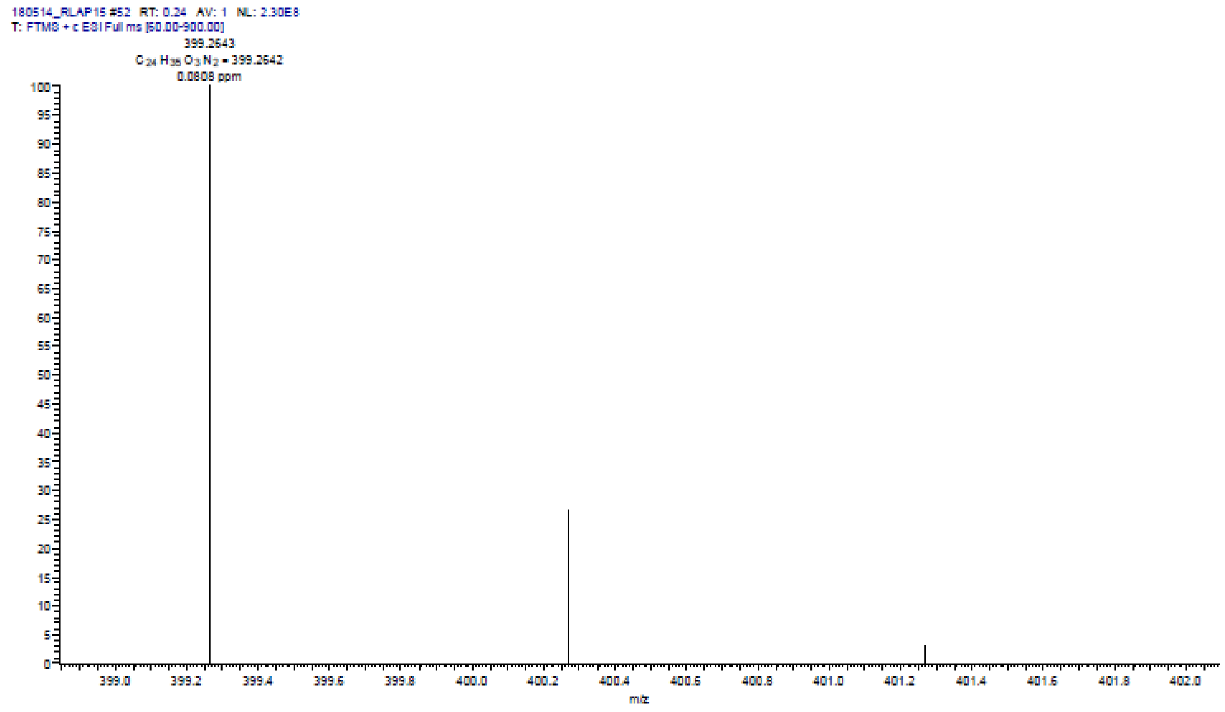

Compound **6** ($C_{28}H_{43}N_2O_3$ $[M+H]^+$):

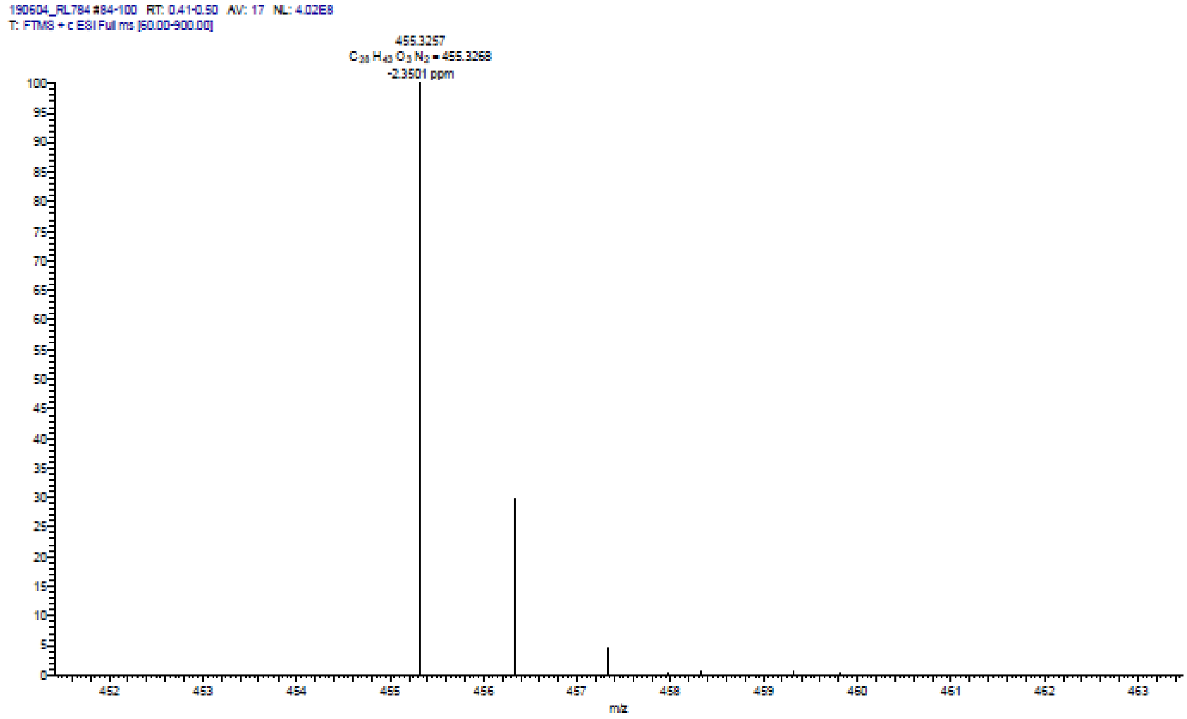

Compound **8** ($C_{23}H_{36}N_3O_3$ $[M+H]^+$):

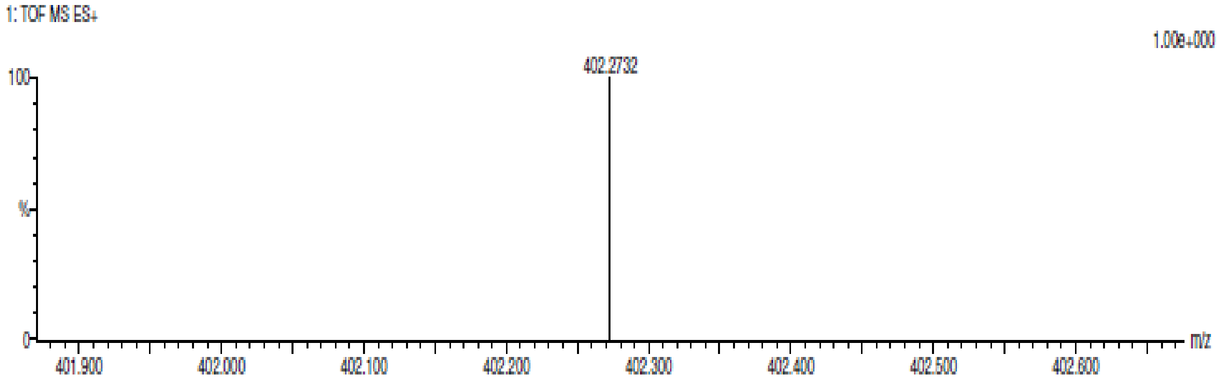

HPLC chromatograms of final compounds 2-8

Compound **2**

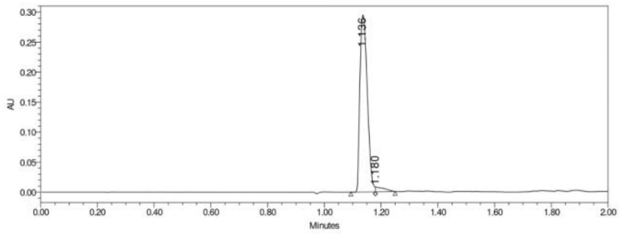

Peak Name:

	Injection	RT	Area	% Area	Height
1	1	1.180	17104	3.10	8009
2	1	1.136	534821	96.90	294818
Mean		1.158			
Std. Dev.		0.031			
% RSD		2.68			

Compound **3**

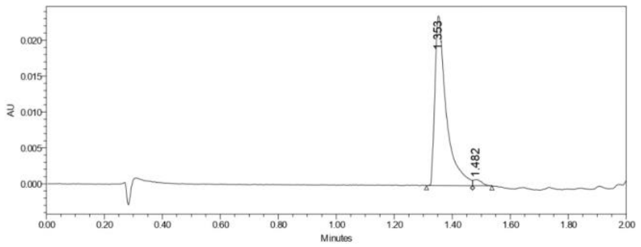

Peak Name:

	Injection	RT	Area	% Area	Height
1	1	1.482	1717	2.58	856
2	1	1.353	64818	97.42	23638
Mean		1.417			
Std. Dev.		0.091			
% RSD		6.44			

Compound **4**

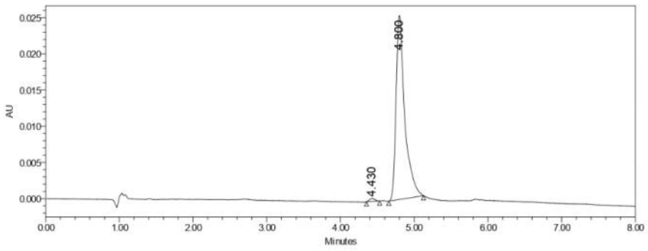

Peak Name:

	Injection	RT	Area	% Area	Height
1	1	4.800	207639	99.00	25433
2	1	4.430	2094	1.00	401
Mean		4.615			
Std. Dev.		0.262			
% RSD		5.68			

Compound **5**

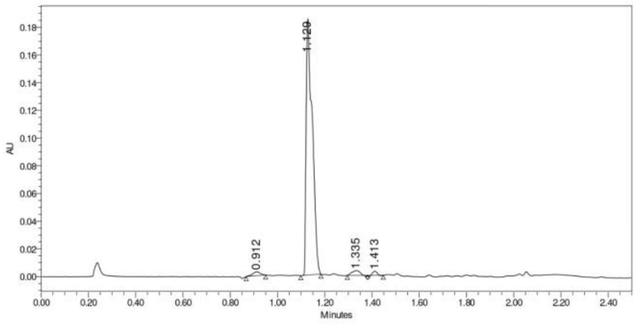

Processed Channel: PDA 280.0 nm

	SampleName	Processed Channel	Retention Time (min)	Area	% Area	Height
1	220517_AP15_Puro	PDA 280.0 nm	0.912	6537	1.84	2879
2	220517_AP15_Puro	PDA 280.0 nm	1.129	334751	94.45	185138
3	220517_AP15_Puro	PDA 280.0 nm	1.335	8716	2.46	3441
4	220517_AP15_Puro	PDA 280.0 nm	1.413	4422	1.25	2911

Compound **6**

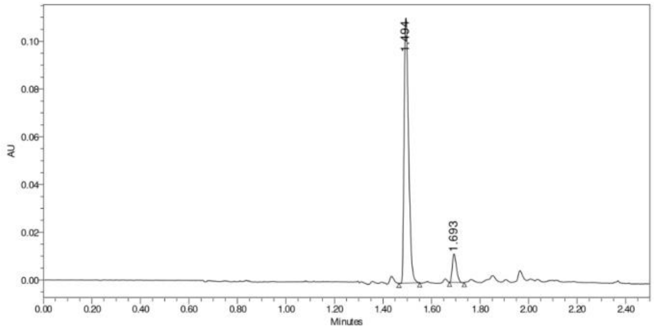

Processed Channel: PDA 280.0 nm

	SampleName	Processed Channel	Retention Time (min)	Area	% Area	Height
1	220517_RL784_C	PDA 280.0 nm	1.494	143085	90.98	11114
2	220517_RL784_C	PDA 280.0 nm	1.693	14194	9.02	11872

Compound **7**

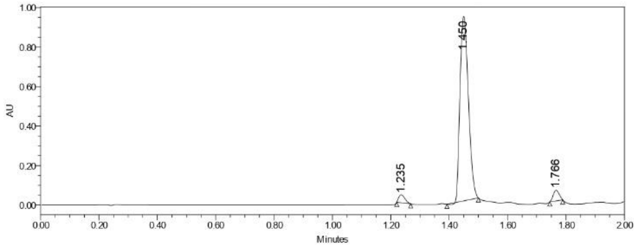

Peak Name:

	Injection	RT	Area	% Area	Height
1	1	1.450	1880293	93.29	934434
2	1	1.235	59416	2.95	40181
3	1	1.766	75826	3.76	53543
Mean		1.484			
Std. Dev.		0.267			
% RSD		18.00			

Compound **8**

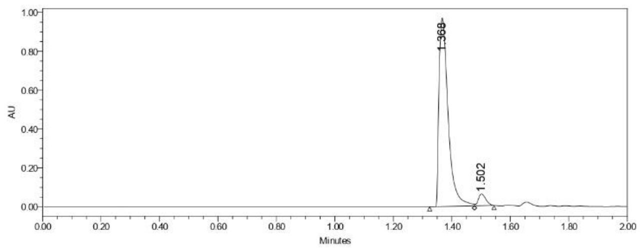

Peak Name:

	Injection	RT	Area	% Area	Height
1	1	1.502	106773	4.82	59711
2	1	1.368	2107288	95.18	970188
Mean		1.435			
Std. Dev.		0.095			
% RSD		6.61			
